# Leveraging the multivalent p53 peptide-MdmX interaction to guide the improvement of small molecule inhibitors

**DOI:** 10.1038/s41467-022-28721-x

**Published:** 2022-02-28

**Authors:** Xiyao Cheng, Rong Chen, Ting Zhou, Bailing Zhang, Zichun Li, Meng Gao, Yongqi Huang, Huili Liu, Zhengding Su

**Affiliations:** 1 Protein Engineering and Biopharmaceutical Sciences Laboratory, Hubei University of Technology, 430068 Wuhan, China; 2 National Center for Magnetic Resonance in Wuhan, State Key Laboratory of Magnetic Resonance and Atomic and Molecular Physics, Innovation Academy for Precision Measurement Science and Technology, CAS, 33 Hongshanche Road, 430071 Wuhan, Hubei China

**Keywords:** X-ray crystallography, Lead optimization, Oncogene proteins, Small molecules

## Abstract

Overexpressed Mdm2 and its 7homolog MdmX impair p53 activity in many cancers. Small molecules mimicking a p53 peptide can effectively inhibit Mdm2 but not MdmX. Here, we show a strategy for improving lead compounds for Mdm2 and MdmX inhibition based on the multivalency of the p53 peptide. Crystal structures of MdmX complexed with nutlin-3a, a strong Mdm2 inhibitor but a weak one for MdmX, reveal that nutlin-3a fits into the ligand binding pocket of MdmX mimicking the p53 peptide. However, due to distinct flexibility around the MdmX ligand binding pocket, the structures are missing many important intermolecular interactions that exist in the MdmX/p53 peptide and Mdm2/nultin-3a complexes. By targeting these flexible regions, we identify allosteric and additive fragments that enhance the binding affinity of nutlin-3a for MdmX, leading to potent Mdm2/MdmX inhibitors with anticancer activity. Our work provides a practical approach to drug design for signal transduction therapy.

## Abstract (web summary)

Peptide fragments derived from the interfaces of protein-protein interactions (PPIs) provide useful templates for designing small molecule PPI inhibitors. Here, the authors utilize the multivalency of an MdmX-binding p53 peptide to develop a weak inhibitor of MdmX into potent Mdm2/MdmX inhibitors.

## Introduction

Protein–protein interactions (PPIs) are fundamental mechanisms of cellular signaling pathways but present a challenge to drug design^1,2^. Many PPI modulators including easily accessible peptides and small molecules developed with more effort, have entered clinical studies, and some have been approved for marketing^3,4^, but the transformation of peptide modulators to small molecules is not yet sufficiently well-understood^5–8^.

The p53-Mdm2/MdmX signaling axis plays an important role in maintaining genome integrity to prevent cancers^9–11^. Mdm2/MdmX are overexpressed in many cancers to impair p53 activity via binding of their N-terminal domains (N-Mdm2 and N-MdmX) to the p53 transactivation domain (p53p)^9,12^. The discovery of effective small-molecule inhibitors of Mdm2 targeting N-Mdm2 is one of the most successful cases of the transformation of peptide modulators into small molecules^13,14^. N-Mdm2 and N-MdmX are homologous and exhibit submicromolar affinity for p53p^15,16^, but these two structurally homologous proteins exhibit different selectivity for Mdm2 small-molecule inhibitors^17–19^. How the conformation of N-MdmX differentially recognized Mdm2 small-molecule inhibitors remains elusive. For example, nutlin-3a is a first-in-class Mdm2 inhibitor with nanomolar binding affinity but a weak MdmX inhibitor^20^, providing an excellent model system for exploring mechanistic questions regarding how homologous proteins differentially recognize ligands and how the flexibility of ligand-binding pockets determine their interaction specificity^17,19,20^.

In this study, we determined crystal structures of MdmX in complex with nutlin-3a. The configuration of nutlin-3a molecule docked in the ligand-binding pocket of MdmX and its intramolecular and intermolecular interactions into MdmX were in detail examined in comparison with those of the MdmX/p53 peptide and Mdm2/nultin-3a complexes. We also determined a crystal structure of MdmX in complex with p53 analog to analyze the p53p multivalency. Furthermore, we identified allosteric and additive fragments that enhanced the binding affinity of nutlin-3a for MdmX, leading to potent Mdm2/MdmX inhibitors with anticancer activity. Thus, our work provides a practical strategy for drug design targeting aberrant protein–protein interactions, via transforming peptide templates and lead compounds into potent small-molecule inhibitors.

## Results

### Crystal structure of N-MdmX in complex with nutlin-3a

**Fig. 1:**
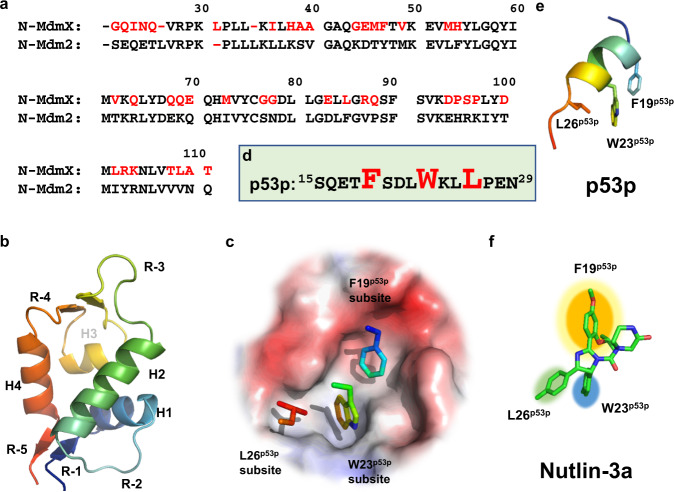
Three subsites on N-MdmX and N-Mdm2 are defined by three key binding residues of p53p. **a** Alignment of the amino acid sequence of N-MdmX with that of N-Mdm2. Non-identical residues on N-MdmX are shown in red. **b** A cartoon model representing N-MdmX and N-Mdm2 structures, based on their crystal structures in complex with p53p. **c** Each ligand-binding pocket on N-MdmX or N-Mdm2 is composed of three subsites, i.e., the F19^p53p^, W23^p53p^, and L26^p53p^ subsites, with reference to the three key binding residues of p53p. **d** The amino acid sequence of the p53p peptide. Three key residues, i.e., F19^p53p^, W23^p53p^ and L26^p53p^, are in red. **e** The structure of p53p with three key residues highlighted. **f** Nutlin-3a can be docked tightly into the three subsites on N-Mdm2, mimicking p53p.

The amino acid sequences and the three-dimensional structures of N-Mdm2 and N-MdmX are homologous (Fig. 1a, b). The structural models of N-MdmX and N-Mdm2 in complex with p53p^20,21^ reveal that the ligand-binding pockets on N-MdmX and N-Mdm2 are surrounded by four intrinsic flexible regions (i.e., R-1, R-3, R-4, and R-5) and two helixes^22–24^ (Fig. 1b). Each ligand-binding pocket can be further dissected into three subsites, i.e., the F19^p53p^ subsite, W23^p53p^ subsite and L26^p53p^ subsite (Fig. 1c), according to the three key binding residues from p53p (Fig. 1d, e). The p53p can bind tightly to both ligand-binding pockets, while Mdm2-only small-molecule inhibitors, such as nutlin-3a (the first discovered Mdm2 inhibitor, Fig. 1f) can effectively imitate p53p to bind tightly to N-Mdm2.

**Fig. 2:**
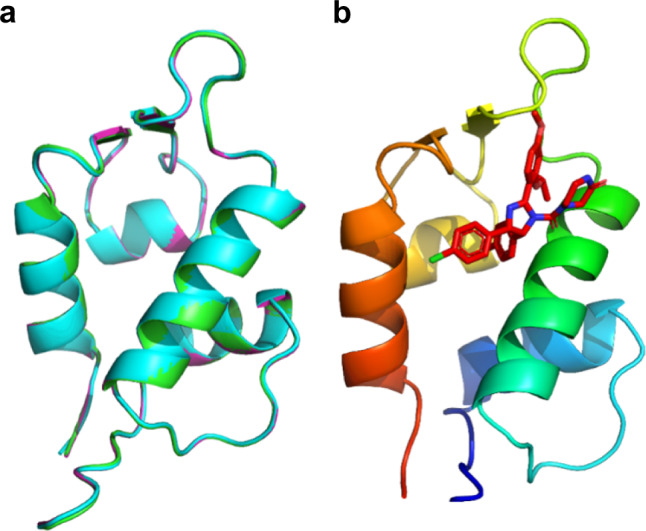
Crystal structures of N-MdmX in complex with nutlin-3a. **a** Comparison of three crystal structures of N-MdmX in complex with nutlin-3a. Blue, green, and magenta represent the crystal structures obtained from the sample Peak 1, the sample Peak 2 and the sample Peak 3 (Supplementary Fig. 1), respectively. **b** A representative crystal structure of N-MdmX in complex with nutlin-3a (red stick).

**Fig. 3:**
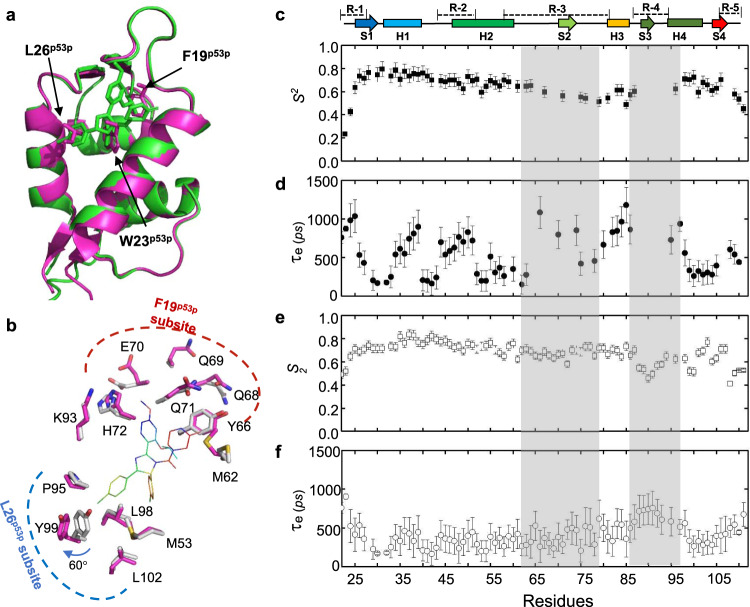
Conformational flexibility weakens the binding affinity of nutlin-3a to N-MdmX. **a** The structures of the N-MdmX/nutlin-3a complexes superimposed on those of the N-MdmX/p53p complexes. Green stick: nutlin-3a*;* purple stick: F19^p53p^, W23^p53p^, and L26^p53p^. **b** Major side-chain conformational deviations occurred at the residues in the F19^p53p^ subsite and L26^p53p^ subsite of N-MdmX in complex with nutlin-3a and p53p. **c**–**f** Fast backbone dynamics (*ps–ns*) of N-MdmX in complex with nutlin-3a (**c**, **d**) or p53p (**e**, **f**). The order parameter (*S*^*2*^) and timescale of motion (τ_e_) of N-MdmX in both complexes are plotted against the backbone amide residue. The secondary structure is shown on the top of the graph. R, S, and H denote the flexible region, β-sheet and α-helix, respectively. Regions with enhanced dynamics are shaded in gray. The data are represented as mean values ± SD and error bars for each parameter represent the propagated uncertainty determined from Monte Carlo simulations. Source data for (**c**–**f**) are provided as a Source Data file.

We determined three crystal structures of N-MdmX in complex with nutlin-3a, isolated at different NaCl concentrations (Supplementary Fig. 1). The protein crystals of the three N-MdmX/nutlin-3a complexes gave good diffraction patterns at 1.63, 1.65, and 1.8 Å (Supplementary Table 1), collected at SSRF^25^. The backbone conformations of the three structures folded in the same pattern (Fig. 2a and Supplementary Fig. 2), and nutlin-3a was located in the ligand-binding pocket of N-MdmX (Fig. 2b). When comparing these N-MdmX structures with those in complex with p53p (3DAB)^21^, we found that their backbone conformations also globally folded in the same pattern (Fig. 3a). Nutlin-3a which mimicked the three key residues of p53p, i.e., F19^p53p^, W23^p53p^, and L26^p53p^, was well docked in the ligand-binding pocket (Fig. 3a). Furthermore, we compared our N-MdmX/nutlin-3a structures with that of the N-MdmX in complex with WK298, a non-nutlin MdmX inhibitor^26^, indicating that nutlin-3a was specific for the ligand-binding pocket on N-MdmX-like WK298 molecule (Supplementary Fig. 3).

Compared with the N-MdmX/p53p complexes, we observed many significant alterations in the side chain configurations of N-MdmX in complex with nutlin-3a, except the W23^p53p^ subsite (Fig. 3b). At the F19^p53p^ subsite of N-MdmX, the side-chain conformations of a few polar residues, including M62, Q68, Q69, E70, Q71, H72, and K93, exhibited significant differences, making this subsite much opener than its counterpart in the N-MdmX/p53p complexes. At this subsite, the N-MdmX/nutlin-3a complexes lost many important intermolecular interactions that were present in the N-MdmX/p53p complexes. Particularly, E17^p53p^ formed an intermolecular salt bridge with K93 of N-MdmX. L22^p53p^ hydrophobically interacted with the aliphatic chain of K93 and the imidazole ring of H72 of N-MdmX (Supplementary Fig. 4).

We also observed significant side-chain shifts at the L26^p53p^ subsite of N-MdmX (Fig. 3b). In particular, the side chain of residue Y99 was shifted away from nutlin-3a by 3.1 Å and rotated by 60°, compared with its counterpart in the N-MdmX/p53p complexes. As a result, the L26^p53p^ subsite, formed by P95, L98, and Y99 in combination with L102 and M53, became much opener than its counterpart in the N-MdmX/p53p complexes.

### Flexibility of the N-MdmX F19^p53p^ subsite is correlated with ligand-binding affinity

To understand how backbone flexibility affects ligand binding, we compared the backbone dynamics of N-MdmX in complex with nutlin-3a or p53p (Fig. 3c–f). Longitudinal and transverse ^15^N-relaxation times and {^1^H}-^15^N NOEs were measured at two magnetic field strengths followed by model-free analysis to yield an order parameter (*S*^*2*^) and timescale of motion (τ_e_) for each observable backbone amide N-H bond vector. *S*^*2*^ can range from 0 (completely unrestricted) to 1 (completely rigid), indicating the degree of motional restriction. The *S*^*2*^ values for residues in N-MdmX in complex with nutlin-3a ranged from 0.20 to 0.82 with an average of 0.60 (Fig. 3c). A plot of *S*^*2*^ as a function of protein sequence revealed several regions with depressed *S*^*2*^ values, indicating enhanced flexibility. These regions included the R-1, R-2, R-3 (residues 54–76), and R-4 region (residues 86–97). Notably, many ^15^N-^1^H amide signals in the R-3 and R-4 regions were still invisible (Fig. 3c, d). On contrast, when complexed with p53p, all the ^15^N-^1^H amide signals from the R-3 and R-4 regions except for proline residues were detectable and became rigid (Fig. 3e, f). Thus, the backbone dynamics of these two regions on the *ps*- *ns* timescale are correlated with ligand-binding affinity, as our ITC experiments indicated that the binding affinity of nutlin-3a for N-MdmX was much weaker than that of p53p (Supplementary Fig. 5).

### Conformational comparison between N-MdmX and N-Mdm2 in complex with nutlin-3a

**Fig. 4:**
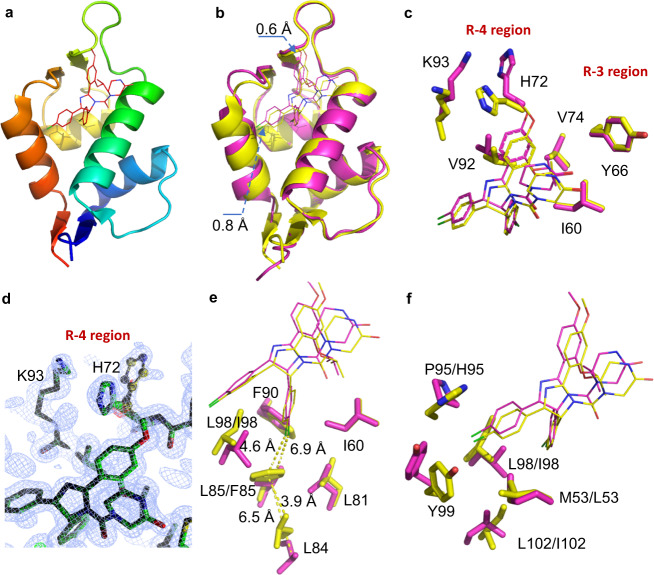
Comparison of N-MdmX and N-Mdm2 structures in complex with nutlin-3a. **a** A representative crystal structure of N-Mdm2 in complex with nutlin-3a (red line). **b** The structure N-MdmX/nutlin-3a complexes (in purple) are superimposed on those of the N-Mdm2/nutline-3a complexes (in yellow). **c** Comparison of major side-chain configurations between the F19^p53p^ subsites of N-MdmX and N-Mdm2. **d** A 2Fo-Fc map contoured at 1.5 σ of a section of the H72 side chain on N-Mdm2 revealing two configurations. **e**, **f** Comparison of major side chain configurations in the W23^p53p^ and L26^p53p^ subsites, respectively, between N-MdmX and N-Mdm2.

Based on our newly determined crystal structure of N-Mdm2/nutlin-3a complexes (1.25 Å, Fig. 4a, Supplementary Table 2, and Supplementary Fig. 6), significant similarities and differences were observed between N-MdmX and N-Mdm2 (Fig. 4b). Both N-MdmX and N-Mdm2 have compact ligand-binding pockets, and nutlin-3a docked in a similar orientation in their ligand pockets. However, nutlin-3a in N-MdmX was slightly shifted toward the F19^p53p^ subsite and L26^p53p^ subsite by 0.6 Å and 0.8 Å, respectively. In the R-3 region of the F19^p53p^ subsite (Fig. 4c), three hydrophobic residues, I60, Y66, and V74 on N-MdmX and N-Mdm2 interacted with nutlin-3a in the same configuration, while in the R-4 region of the F19^p53p^ subsite, the residues V92, H72, and K93 on N-MdmX shifted significantly away from nutlin-3a compared with their counterpart on N-Mdm2 (Fig. 4c). We observed that in both complexes, the K93 and H72 residues formed a cation–π pair interaction (Fig. 4c). This cation–π pair on N-Mdm2 was positioned close to nutlin-3a and interacted with nutlin-3a, while the cation–π pair in N-MdmX was distant from nutlin-3a. We found that in N-Mdm2 this cation–π pair existed in two distinct configurations (Fig. 4d). Based on electronic density, it was estimated that 60% of its H72 configuration was in the position closer to nutlin-3a (the bound state), while 40% of its configuration was in the position far away from nutlin-3a (the unbound state). We noticed that in the N-MdmX/p53p complexes, this cation–π pair was located in a half-bound state interacting with E17^p53p^ and L22^p53p^ (Supplementary Fig. 7a, b). However, in the N-MdmX/nutlin-3a complexes this pair was completely located in an unbound state (Fig. 4c). Furthermore, we conducted a molecular dynamics simulation on the dihedral angle of H72 on N-Mdm2 and N-MdmX, which indicated that this kind of bound–unbound transition likely existed in N-MdmX in complex with nutlin-3a (Supplementary Fig. 7c).

In both N-MdmX and N-Mdm2, the W23^p53p^ subsites consisted of hydrophobic residues including I60, L81, L84, L85 (F85), F90, and L98 (I98) (Fig. 4e). We observed that residue F85 of N-Mdm2 was mutated to L85 in N-MdmX, which could slightly affect the interaction with nutlin-3a. The L26^p53p^ subsite, in N-Mdm2, consisted of L53, H95, I98, and Y99 (Fig. 4f), forming a hydrophobic cavity to tightly interact with nutlin-3a. Conversely, this hydrophobic groove on N-MdmX was affected by the L53M mutation (Fig. 4f), which weakened the interaction of Y99 with L98.

**Fig. 5:**
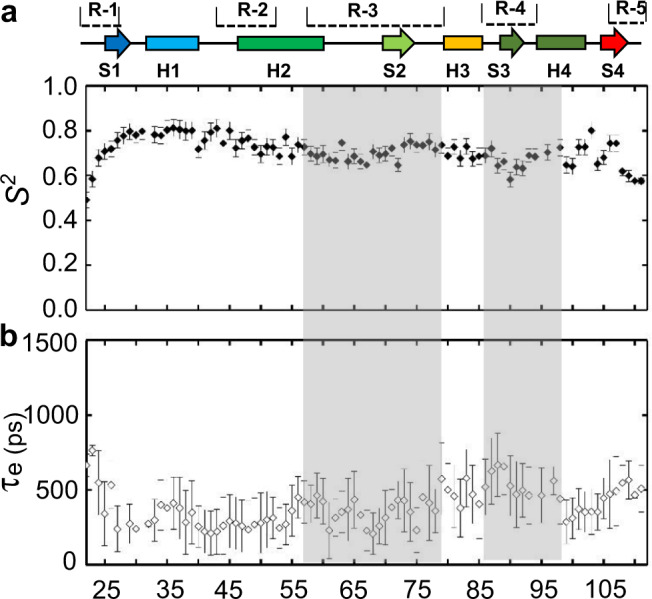
Fast backbone dynamics (*ps–ns*) of N-Mdm2 in complex with nutlin-3a. The order parameter (**a**, *S*^*2*^) and timescale of motion (**b**, τ_e_) of N-Mdm2 in complex with nutlin-3a are plotted against the backbone amide residue. The secondary structure is shown on the top of the graph. R, S, and H denote the flexible region, β-sheet, and α-helix, respectively. Regions with enhanced dynamics are shaded in gray. The data are represented as mean values ± SD and error bars for each parameter represent the propagated uncertainty determined from Monte Carlo simulations. Source data for (**a**, **b**) are provided as a Source Data file.

Furthermore, we characterized the backbone amide dynamics of N-Mdm2 in complex with nutline-3a on the *ps*~*ns* timescale (Fig. 5a, b). Compared with the conformational dynamics of N-MdmX in complex with nutlin-3a (Fig. 3c, d), the binding of nutlin-3a to N-Mdm2 made the residues in the R-3 and R-4 regions rigid (Fig. 5a, b). Taken together, the structural evidence indicated that the weak affinity of nutlin-3a for N-MdmX was caused not only by weak hydrophobic interactions with the three subsites but also by conformational flexibility around the R-3 and R-4 regions in its F19^p53p^ subsite.

### Identification of fragments rigidifying the N-MdmX F19^p53p^ subsite

**Fig. 6:**
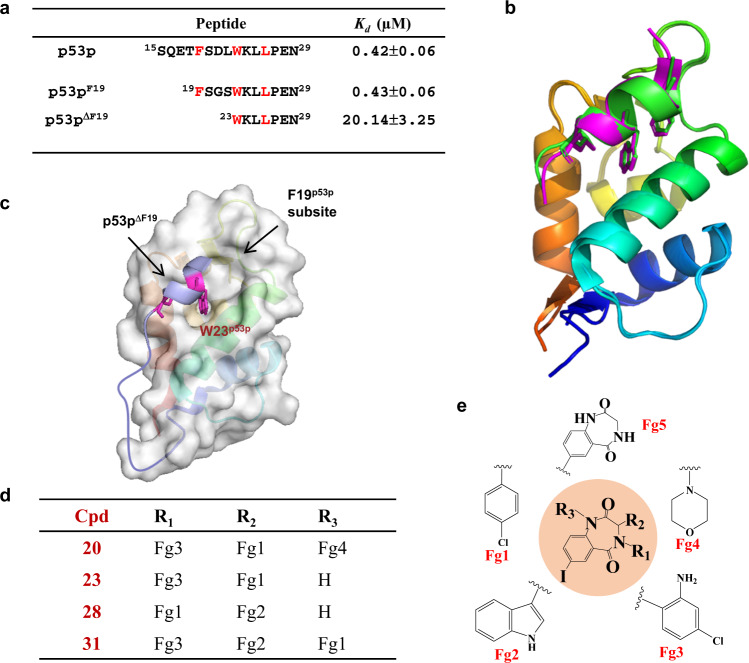
Identification and characterization of additive and allosteric fragments enhancing the binding affinity of p53p for N-MdmX. **a** Affinity comparison of p53p^F19^ with p53p and p53p^ΔF19^. *n* = 3 independent experiments and the data are represented as mean values ± SD. Source data are provided as a Source Data file. **b** The crystal structure of N-MdmX in complex with p53p^F19^ (purple) is superimposed on that of N-MdmX in complex with p53p (green). **c** A p53p^ΔF19^-MdmX fusion protein was constructed for screening pharmacophores that could specifically target the F19^p53p^ subsite on N-MdmX. **d**, **e** Four benzodiazepine analogs (**d**) and their pharmacophores (**e**).

To understand how the N-MdmX F19^p53p^ subsite thermodynamically interacts with the F19^p53p^ residue, we substituted the amino acid sequence between F19^p53p^ and W23^p53p^ with a flexible linker to form a p53p analog (p53p^F19^, Fig. 6a). We found that p53p^F19^ did not reduce the p53p-binding affinity determined by ITC (Fig. 6a and Supplementary Fig. 5c). Furthermore, we resolved the crystal structure of N-MdmX in complex with p53p^F19^ (Fig. 6b, Supplementary Table 3, and Supplementary Fig. 8), which showed no significant derivation in its conformation compared with that of the N-MdmX/p53p complexes (3DAB). These data indicated that F19^p53p^ contributed to the binding affinity of p53p to N-MdmX only in an additive mode.

**Fig. 7:**
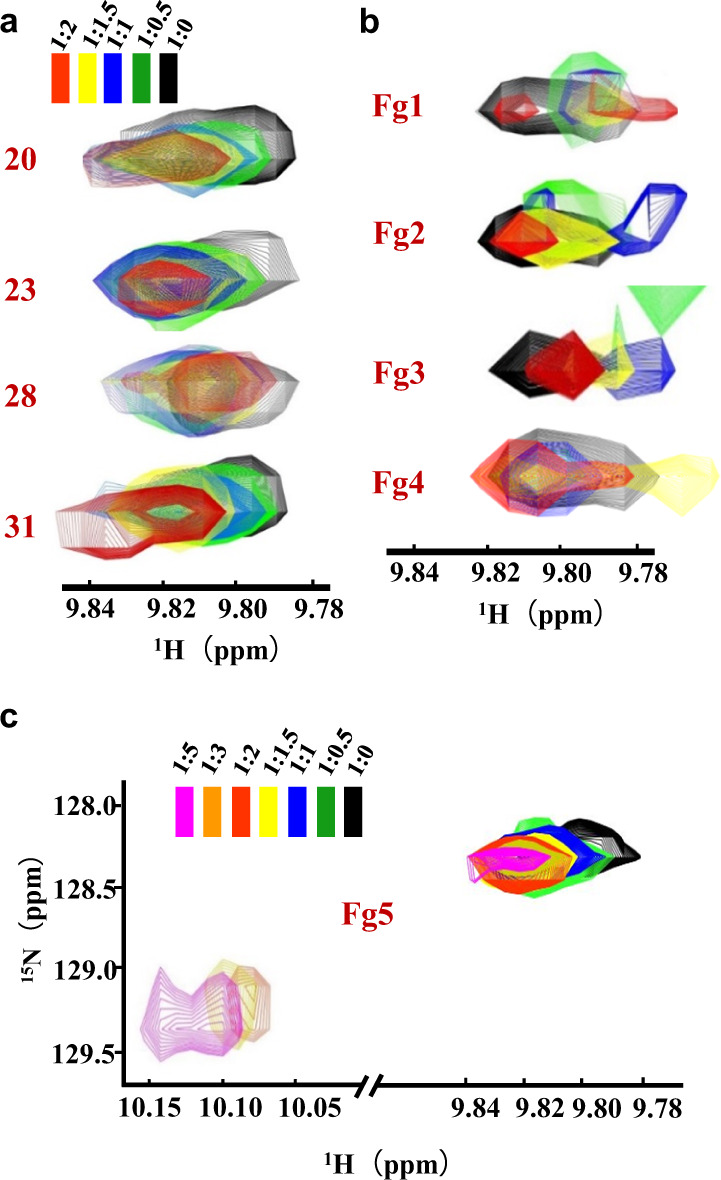
^15^N-^1^H HSQC spectrometric characterization of benzodiazepine compounds enhancing the binding affinity of p53p for N-MdmX. **a** The perturbation of the ^15^N-^1^H HSQC resonances of the W23^p53p^ side chain by four benzodiazepine compounds are compared. Protein and ligand ratios: black, 1:0; green, 1:0.5; blue, 1:1; yellow, 1:1.5 and red, 1:2. **b**, **c** The perturbation of the ^15^N–^1^H HSQC resonances of the W23^p53p^ side chain by five pharmacophores are compared. The protein and ligand ratios: black, 1:0; green, 1:0.5; blue, 1:1; yellow, 1:1.5; red, 1:2, orange, 1:3 and purple, 1:5.

Next, we attempted to identify pharmacophores that allosterically enhanced the binding affinity of p53p for the F19^p53p^ subsite. For this goal, a p53p^ΔF19^ peptide was tethered to N-MdmX to construct a fusion protein model (p53p^ΔF19^-N-MdmX), as previously reported by Chen et al*.*^27^, so that its F19^p53p^ subsite remained empty, as shown in Fig. 6c. In this fusion protein, the W23^p53p^ from p53^ΔF19^ fragment is only one tryptophan residue, functioning as not only a key residue for binding of p53p fragment to N-MdmX but also an intrinsic fluorescence signal to probe the association/dissociation of p53p fragment with N-MdmX. As depicted in Supplementary Fig. 9a, when an allosteric fragment binds to the F19^p53p^ subsite, the W23^p53p^ fluorescence signal of p53^ΔF19^ fragment will be enhanced at 324 nm. If a fragment displaces the p53^ΔF19^ fragment, the W23^p53p^ fluorescence signal of will be attenuated and its maximum emission wavelength will be shifted to 350 nm. Therefore, in this fusion protein model, the intrinsic fluorescence signal of the W23^p53p^ side chain was sensitive to protein conformational change caused by ligand binding. By screening a previously constructed small compound library of Mdm2 inhibitors^27^, we identified four compounds (i.e., Cpds 20, 23, 28, and 31 in Fig. 6d) that enhanced the fluorescence signal of W23^p53p^ (Supplementary Fig. 9b). All the four compounds were benzodiazepine analogs and their functional groups could be dissected into five fragments (Fig. 6e), i.e., Fg1, Fg2, Fg3, Fg4, and Fg5. Verified by ^15^N-^1^H HSQC NMR titration experiments, the four compounds perturbed many ^15^N-^1^H NMR resonances of the fusion protein when the ligand concentration was increased to adjust the ratio of protein and ligand from 1:0 to 1:2 (Supplementary Fig. 10). In particular, as shown in Fig. 7a, Cpds 20, 23, and 31 significantly perturbed the W23^p53p^ side-chain ^15^N-^1^H resonances, while Cpd 28 broadened the W23^p53p^ side-chain resonance peak.

To further evaluate how each fragment allosterically enhances the binding affinity of p53p^ΔF19^, we titrated the ^15^N-^1^H NMR HSQC spectra of the fusion protein with individual fragments at protein and fragment ratio up to 1:2. As shown in Supplementary Figs. 11 and 12, none of these fragments significantly perturbated ^15^N-^1^H NMR HSQC resonance peaks except for Fg5. Regarding the resonance peaks of the W23^p53p^ side chain, Fg1, Fg2, Fg3, and Fg4 caused no significant resonance perturbation when each was titrated in protein and fragment ratio from 1:0 to 1:2 (Fig. 7b), while Fg5 caused significant resonance perturbation (Fig. 7c). Notably, when Fg5 was titrated in protein to fragment ratio over 1:2, Fg5 dramatically shifted the resonance peak of the W23^p53p^ side chain to 10.10 ppm (Fig. 7c). As a result, Fg5 is a potent allosteric pharmacophore that enhances the binding affinity of p53p^ΔF19^ to N-MdmX.

### Rational transformation of p53p to small molecules

**Fig. 8:**
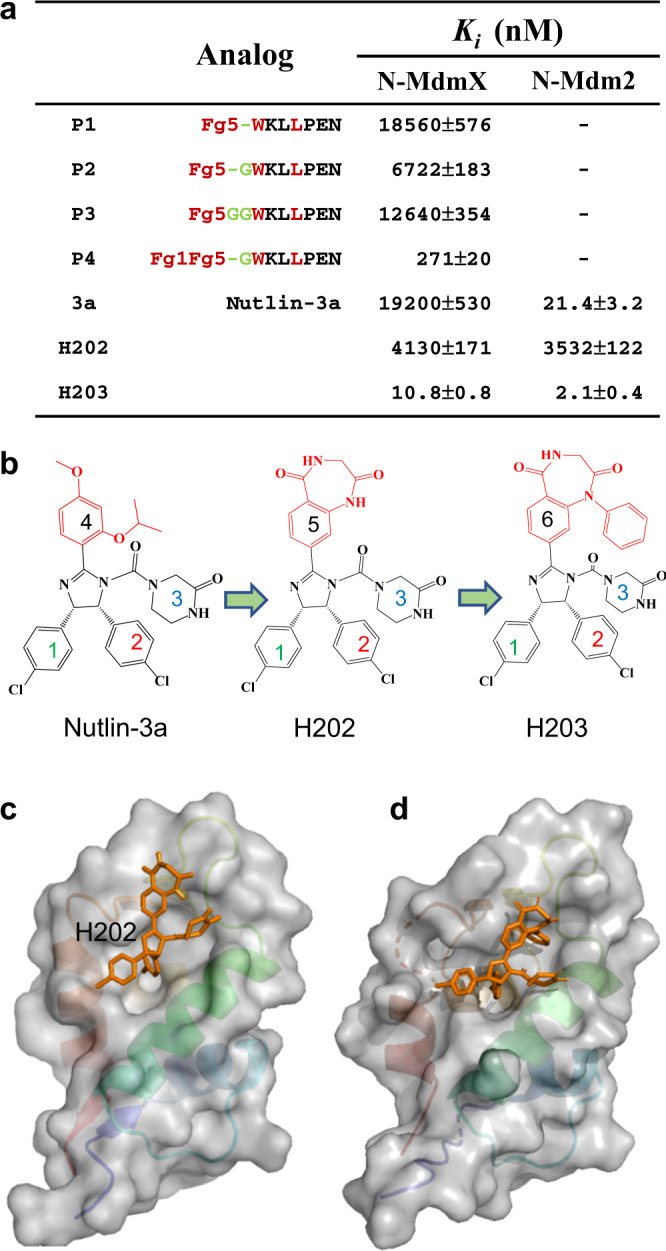
Rational transformation of p53p into small-molecule inhibitors of MdmX. **a** The structure and activity relationships of p53p and nutlin analogs are compared based on Fg1 and Fg5. *n* = 3 independent experiments and the data are represented as mean values ± SD. Source data are provided as a Source Data file. **b** Molecular structures of nutlin analogs H202 and H203. **c**, **d** Structure models of N-MdmX interacting with H202 and H203, evaluated by molecular docking.

Next, we attempted to transfer Fg5 to nutlin-3a, and tested the bivalency effect of Fg5 on the p53p^ΔF19^ peptide. First, we conjugated Fg5 to p53p^ΔF19^ with different lengths of linkers to generate three peptide analogs (P1, P2, and P3, Fig. 8a). Among the three peptide analogs, P2 exhibited the best affinity for N-MdmX with a *K*_*i*_ of 6.72 μM (Fig. 8a). Next, we transferred Fg5 to nutlin-3a by replacing its functional group 4 with Fg5 (Fig. 8b). A resultant nutlin analog (H202) was chemically synthesized (Supplementary Figs. 13 and 14). H202 exhibited an enhanced binding affinity for N-MdmX with a *K*_*i*_ of 4.13 μM (Fig. 8a and Supplementary Fig. 15). Evaluated with molecular docking, Fg5 on H202 functions as a lid to cover the F19^p53p^ subsite on N-MdmX and interacts with the R-3 and R-4 regions (Fig. 8c). Notably, under the lid, the subsite remains empty. As indicated by our crystal structure of N-MdmX/p53p^F19^ (Fig. 6b), the benzene group from F19^p53p^ should be a good candidate to fill up the empty space. Thus, we next introduced benzene into Fg5 to obtain functional group 6 (Supplementary Fig. 16) and prepared H203 (Fig. 8b and Supplementary Figs. 13 and 17). Molecular docking indicated that the function group 6 in H203 fits very well at the F19^p53p^ subsite (Fig. 8d). Quantitatively, H203 exhibited an enhanced binding affinity for N-MdmX with a *K*_*i*_ of 0.011 μM (Fig. 8a and Supplementary Fig. 15), which was nearly1400-fold higher than that of nutlin-3a. Our data also revealed that H203 retained a high binding affinity for N-Mdm2 with a *K*_*i*_ of 0.002 μM (Fig. 8a and Supplementary Fig. 15).

**Fig. 9:**
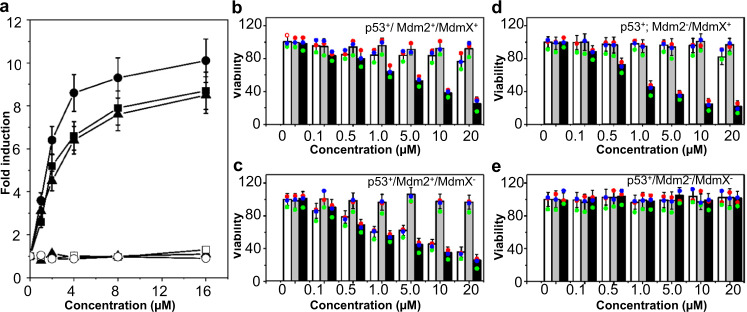
Biological activity of H203. **a** Treatment of cancer cells with H203 induced the expression of the p21 gene but not the p53 gene. Solid and open symbols indicate p21 and p53 transcription levels, respectively. Triangle: H460a cell; circle: RKO cell; and square: HCT116 cell. *n* = 3 independent experiments, and the data are represented as mean values ± SD. **b**–**e** The viability of the H1299 cells overexpressing Mdm2 and/or MdmX was significantly influenced by H203 compared with nutlin-3a and nutlin-3b. *Empty* bar: nutlin-3a (a positive control); *gray* bar: nutlin-3b (a negative control); and *dark* bar: H203. H1299^*p53+/Mdm2+/MdmX+*^ cells (**b**); H1299^*p53+/Mdm2+/MdmX-*^ cells (**c**); H1299^*p53+/Mdm2-/MdmX+*^ cells (**d**); and H1299^*p53+/Mdm2-/MdmX−*^ cells (**e**). *n* = 3 independent experiments and the data are represented as mean values ± SD. Source data for (**a**–**e**) are provided as a Source Data file.

To test whether H203 could induce the p53 pathway in vivo by inhibiting MdmX binding to p53, we treated three cancer cell lines expressing wild-type p53, HCT116, RKO, and H460a, with H203 for 8 h and measured the expression of the p53 and p21 genes (Fig. 9a). The transcription of p21 increased in a dose-dependent manner in all three cell lines, which was consistent with the accumulation of its transcriptional activator, p53. In contrast, the transcription of the p53 gene itself was unaffected by H203. Furthermore, we conducted MTT assay using H1299 cell lines with GFP-tagged p53 expression inducible by G418 (H1299^*p53+*^)^28^. In this study, for a proof of concept, the overexpressed Mdm2 and MdmX in H1299 cells were achieved by the transient transfection of the RFP-tagged MdmX and Mdm2 expression plasmids (see “Methods“). The overexpressed Mdm2 and/or MdmX in the H1299^*p53+*^ were strong inhibitors to cellular p53 protein. The cells were exposed to H203, nutlin-3a (the positive control) or nutlin-3b (the negative control) for 48 h, and cell growth/viability was measured. The results showed that H203 significantly reduced cell viability in the cells overexpressing both Mdm2 and MdmX (Fig. 9b), and a significant and dose-dependent decrease in cell number was also observed in H203-treated cells lacking Mdm2 (Fig. 9c) or MdmX (Fig. 9d). Cells lacking both Mdm2 and MdmX were largely unaffected by H203 treatment (Fig. 9e). In contrast, nutlin-3a exhibited strong inhibition of the cells overexpressing Mdm2 (Fig. 9c), but not those overexpressing MdmX (Fig. 9b, d). Importantly, when model cells simultaneously overexpressed Mdm2 and MdmX, the inhibitory activity of the Mdm2-specific inhibitor, nutlin-3a, was significantly attenuated (Fig. 9b). Furthermore, our western blotting assays confirmed the specificity of H203  for both Mdm2 and MdmX (Supplementary Fig. 18). As shown in Supplementary Fig. 18a, H203 significantly prevented p53 degradation by inhibiting MdmX and Mdm2 at a concentration of large than 1.0 μM. In this work, as H203 had high affinity for both Mdm2 and MdmX, it exhibited comparable activity against the H1299^*p53+/Mdm2+/MdmX+*^ cells (Supplementary Fig. 18a), H1299^*p53+/Mdm2-/MdmX+*^ cells (Supplementary Fig. 18b) and the H1299^*p53+/Mdm2+/MdmX*−^ cells (Supplementary Fig. 18c), while the Mdm2 inhibitor nutin-3a exhibited activity only for the H1299^*p53+/Mdm2+/MdmX*−^ cells (Supplementary Fig. 18c). After H203 treatment, the upregulation of p21 and PUMA proteins in H1299^*p53+/Mdm2+/MdmX+*^ cells, H1299^*p53+/Mdm2+/MdmX−*^ cells and H1299^*p53+/Mdm2-/MdmX+*^ cells were in consistent with increasing p53 activity (Supplementary Fig. 18a–c), while the nutlin-3a treatment upregulated p21 and PUMA expression in H1299^*p53+/Mdm2+/MdmX−*^ cells (Supplementary Fig. 18c). Both H203 and nutlin-3a exhibited no effects on the cells lacking of overexpressed Mdm2 and MdmX (Supplementary Fig. 18d). These results suggested that the H203-mediated decrease in cell viability was strictly p53-dependent and that H203 affected MdmX more specifically than nutlin-3a, providing a potent dual MdmX/Mdm2 inhibitor.

Encouraged by these observations, we would like further to optimize H203 by enhancing the binding of inhibitors for L26^p53p^ subsite. Based on the previous exploration on SAR of MdmX and Mdm2 inhibitors^23,24,29–31^, we explored the SAR of the H203 binding group for L26^p53p^ subsite using molecular docking. A potent nutlin analog was sketched and defined as H210 (Supplementary Fig. 19). Theoretically, H210 exhibited a strong binding affinity for N-MdmX at low nanomolar concentration. Conversely, this compound reduced its binding for N-Mdm2 (Supplementary Fig. 19c). As a result, H210 is likely an effective MdmX inhibitor. Currently, we have already started to synthesize H210 for its biochemical and cell activity assays (Supplementary Fig. 20).

## Discussion

To date, many efforts have been made to improve the binding affinity of nutlin-3a for MdmX via high-throughput screening^32,33^, SAR-based modification^34,35^, computational modeling^36,37^ and structural biology approaches^23^. In this work, we determined crystal structures of MdmX in complex with nutlin-3a. The structures reveal that nutlin-3a docks into the N-MdmX-binding pocket in the appropriate orientation to p53p (Fig. 3a), as it does in N-Mdm2 (Fig. 4b), suggesting that small-molecule Mdm2 inhibitors are excellent “drug leads” for affinity optimization. By comparison, nutlin-3a loses many important intermolecular interactions with MdmX that exist in the Mdm2/nutlin-3a and the MdmX/p53p complexes, reducing its affinity to MdmX.

Previous and current NMR studies indicated the F19^p53p^ subsite (Fig. 3c, d) were highly flexible^22,24^. This subsite is not sensitive to nutlin-3a binding, while its counterpart on Mdm2 becomes rigid upon nutlin-3a binding (Fig. 5). By compound screening, we identified benzodiazepine to allosterically rigidify the F19^p53p^ subsite. Furthermore, we found that the F19^p53p^ residue simply contributed to p53p binding in an additive mode (Fig. 6b). Through integrating benzodiazepine and the side chain of F19^p53p^ residue into the imidazole scaffold of nutlin-3a, we obtained few potent nutlin analogs with anticancer activity. Therefore, our work demonstrated a general strategy to aid the transformation of peptide modulators into small molecules for drug design targeting aberrant protein–protein interactions, based on potent peptides and lead compounds. Previously, conformational discrepancies between N-MdmX and N-Mdm2 in complex with nutlin-3a were predicted simply based on the N-MdmX structure in complex with p53p, and such prediction missed many important conformational changes. Thus, our structures of N-MdmX in complex with nutlin-3a can provide new rationale for the development of MdmX inhibitors, in combination with the high-throughput screening of compound libraries and drug design approaches^34,38^.

More importantly, our results support the previous synergistic concept for the protein–ligand interaction mechanism in which protein-binding pocket dynamics control ligand binding^39,40^, while multivalency is a thermodynamic principle of ligand binding^41^. Restraining the conformational flexibility of the MdmX-binding pocket benefits ligand binding^24,31^, and vice versa, the binding of the ligand to N-MdmX rigidifies its ligand-binding pocket^23,42^. The current work revealed that the MdmX/Mdm2 binding pocket dynamics on the *ps–ns* timescale significantly orchestrated the differential binding of the small-molecule nutlin-3a to MdmX and Mdm2, although the effect of Mdm dynamics on the *μs–ms* timescale on nutlin-3a binding has not yet been explored. Considering that many ^15^N-^1^H HSQC NMR resonances from the free N-MdmX NMR spectrum remain missing even in complex with nutlin-3a^22,24^, it is likely that the Mdm *μs–ms* dynamics may be correlated with in ligand-binding functioning as an indicator to monitor ligand binding. In perspective, the invisible conformational state of MdmX can help to understand the ligand-binding process and selectivity with the advent of advanced NMR dynamics techniques^43,44^. Therefore, this study illustrates a need for future research to examine the correlation between the conformational flexibility of the ligand-binding pocket and ligand multivalency in protein–ligand interactions.

## Methods

### Cell lines and reagents

HCT116, RKO, H164a, and H1299 cells were purchased from ATCC and were cultured in a 37 °C incubator with 5% CO_2_ according to ATCC protocols. Nutlin-3a were purchased from APExBIO (TX, USA). Lipofectamine 2000 was purchased from Invitrogen (Shanghai, China). The peptides including p53p, fluorescein-labeled p53p (Flu-p53p), and a mutant p53 peptide (p53p^AAA^) where three key residues were substituted by alanine were synthesized by TOPE Biotech (Shanghai, China). Peptide analogs were prepared by Huazheng Biotech (Changzhou, China). 1-chloro-4-ethynylbenzene (CAS#: 873-73-4), 1,3-dichloro-5-iodobenzene (CAS#3032-81-3), 2-amino-4-methylbenzoic acid (CAS#: 2305-36-4), 1,2-Bis (4-chlorophenyl) ethane-1,2-diamine (CAS#: 86212-34-2), 2,5-dioxo-2,3,4,5-tetrahydro-1H-benzo[1,4]diazepine-8-carboxylic acid (CAS#: 195985-12-7), 1-(tert-Butoxycarbonyl)-5-oxopiperazine-2-carboxylic acid (CAS#: 1246553-28-5) were purchase from Bidepharm (Shanghai, China). 1-(4-chlorophenyl)-2-(3,5-dichlorophenyl) ethane-1,2-diamine and 2,5-dioxo-1-phenyl-2,3,4,5-tetrahydro-1H-benzo-[1,4]diazepine-8-carboxylic acid were synthesized by Huazheng Biotech (Changzhou, China).

### Protein expression and purification

A modified pET28b plasmid containing the p53-binding domain of human MdmX (N-MdmX, amino acids 22–110) and human Mdm2 (N-Mdm2, amino acids 22–110) were constructed previously in our laboratory^24^. The p53p^ΔF19^ gene was synthesized by GenScript (Wuxi, China) and sub-cloned to the pET28-MdmX plasmid.

Recombinant N-MdmX and N-Mdm2 proteins were prepared in *E. coli* BL21 (*DE3*) cells. Cells were grown in LB medium containing kanamycin (34 μg/mL) and induced with 0.4 mM IPTG at 18 °C for 12 h. For heteronuclear NMR experiments, protein samples were uniformly labeled with ^15^N and ^13^C in MOPS medium with BME vitamins containing 1 g/L (^15^NH_4_)_2_SO_4_ and 2 g/L [^13^C_6_] glucose as the sole sources of nitrogen and carbon, respectively. Cells were harvested by centrifugation at 5000 × *g* for 30 min, resuspended in a buffer containing 10 mM Tris-HCl, 40 mM NaCl, 2 mM β-mercaptoethanol, 2 mM imidazole, pH 8.0 (Buffer A), and lysed by sonication and homogenization, followed by spinning at 18,000 × *g* for 30 min. The supernatant was loaded onto a 5 mL Ni-NTA agarose column (Qiagen, USA) and His-tagged protein was competitively eluted using a gradient of Buffer A mixed with Buffer B containing 10 mM Tris-HCl, 40 mM NaCl, 2 mM β-mercaptoethanol, 300 mM imidazole, pH 8.0. The eluate was diluted in 20 times with a buffer containing 20 mM sodium citrate (pH 6.5), 10% glycerol, 2 mM β-mercaptoethanol. The supernatant was loaded onto a MonoS10/100 cation exchange column (GE, USA) which was pre-equilibrated with 10 column volumes (CV) of Buffer MA (20 mM sodium citrate, 25 mM NaCl, 10% Glycerol, 2 mM β-mercaptoethanol, pH 6.5). The protein was eluted with a linear gradient of NaCl from Buffer MA to Buffer MB (20 mM NaCl, 1 M NaCl, 10% glycerol, 2 mM BME, pH 6.5). Peak fractions were separately collected, and mixed with 2 mM DTT and 2% 18-crown-ether.

The purification of the p53p^ΔF19^-MdmX fusion protein essentially followed the same procedure as above, except that the pH value of samples was adjusted to 7.16 by adding a buffer containing 50 mM Tris-HCl (pH 8.0), 200 mM NaCl, 10 mM imidazole, 2 mM β-mercaptoethanol.

The purified N-MdmX, N-Mdm2, and the p53p^ΔF19^-MdmX protein were respectively concentrated to 0.4–0.8 mg/mL and 10–25 mg/mL, and freshly frozen in liquid nitrogen, and kept at −80 °C.

### Protein crystallization

The conditions of protein crystallization were screened against different kits including Index^TM^, PEGRx, and Crystal Screen^TM^ (Hampton, USA) and the AmSO_4_ Suite, the JCSG^+^ Suite, and the PEGs Suite (Qiagen, USA) using GRYPHON station (ARI, USA). Diamond crystals appeared within two days in the condition.

Protein crystallization was further optimized using a sitting drop method. Briefly, 0.6 μL protein drop was composed of 0.3 μL protein sample (10 mg/mL), 0.3 μL reservoir solution. The drop was incubated at 18 °C. Further, large amounts of protein crystal were reproduced by seeding procedure. New drops of mother liquid were inoculated with crushed needle crystal suspended in a slurry mother solution. Diamond crystals grew after 2 days. The crystals were transferred to a cryoprotection solution containing 50% (v/v) reservoir solution and 25% (v/v) glycerin and quickly cooled in liquid nitrogen.

### Data collection and refinement of protein crystallography

The X-ray diffraction data were collected by HKL 2000 on BL17U1 at Shanghai Synchrotron Radiation Facility (SSRF) using the EigerX16M detector^25^. The datasets were integrated, scaled, reduced, and phased using AutoPROC, XDS, DIALS, Porpoise, and Xia2 programs. Model was built with Basic Molecular Replacement - PHASER in Phenix package using Mdm2 structure from 4J3E as a template for N-Mdm2, and MdmX structures from 6Q9W and 6V4F as a template for N-MdmX. Refinement was done with REFMAC5 in the CCP4i2 package^45,46^.

### NMR ^15^N-^1^H HSQC titration and dynamics experiments

Protein samples were prepared through buffer exchange with the NMR buffer. The concentration of the protein complex was 0.3–0.4 mM. All NMR spectra were collected at 25 °C on a Bruker Avance 600 MHz or 800 MHz spectrometer equipped with a triple-resonance pulsed-field gradient probe. ^15^N−^1^H HSQC NMR spectra were recorded in the States-TPPI mode for quadrature detection. All the NMR samples were prepared in a buffer containing 20 mM sodium phosphate, 200 mM NaCl, 2 mM DTT, and 95% H_2_O/5% D_2_O at pH 6.8. The final datasets were acquired with 2048 complex points in t_2_ and 128 complex points in t_1_. All datasets were processed using Topspin or NMRPipe. Spectral display, assignments, and analysis were performed using the NMRViewJ software package. ^15^N T_1_ and T_2_ relaxation times and the heteronuclear NOE values were measured using ^15^N-labeled protein complexed with nutlin-3a pr p53p using the pulse sequences described by Farrow et al.^47^. Relaxation delays of 2.8 s were used for the T_1_ and T_2_ datasets, and a 5 s recycle delay was used for the heteronuclear NOE experiment. Longitudinal relaxation delays of 10, 70 (2*), 150, 250, 320, 520 (2*), 760, 1100 (2*), 1500, 2000, and 3600 ms (where 2* represents a duplicate measurement) and T_2_ delays of 14.4 (2*), 28.8, 43.2, 57.6, 72 (2*), 86.4, 100.8, 115.2 (2*), 144, and 172.8 ms were sampled for all complexes. Steady-state heteronuclear NOE data were obtained in an interleaved manner with and without proton pre-saturation (3 s). To minimize heating between the T_2_ time points, the total number of CPMG pulses was kept constant for each time point by introducing the appropriate number of dummy CPMG pulses^48,49^. Errors in the heteronuclear NOE values were estimated from the root-mean-square variation of noise in empty regions of the two spectra as previously described^50^.

Uncertainties in peak heights for *R*_*1*_ and *R*_*2*_ measurements were estimated from duplicate datasets. Values for R_1_ and R_2_ and the uncertainties were determined by nonlinear least-squares fitting of experimental data to mono-exponential functions using GUARDD with MATLAB 2018a. Errors for the heteronuclear NOE values were estimated from the root-mean-square variation of noise in empty regions of the two spectra as described previously^51^.

^15^N-^1^H HSQC titrations were performed by stepwise addition of compounds (at a high concentration) into the ^15^N-labeled component (typically at a concentration of 0.1–0.3 mM) to a final 1-5-fold excess. Minimal changes in volume and pH were ensured throughout the NMR sample preparations.

### Fluorescence polarization (FP) assay

All fluorescence polarization assays were done using black, low-protein-binding 96-well plates (Corning, NY) in a total volume of 100 μL per well of 20 mM phosphate (pH 6.8), 200 mM NaCl, and 1 mM DTT. After a preincubating of 75 nM fluorescein-p53p (fluorescein-GSGSSQETFSDLWKLLPEN, Flu-p53p) with 1.5 μM N-MdmX for 30 min, a compound was then added and the mixture incubated for an additional 30 min. FP readings were taken with a 555 nm excitation filter and a 632 nm static and polarized filter on a BioTek H1 multiplate reader with Gen5 software. The unlabeled p53p peptide and nutlin-3a were used as positive controls. A mutant p53 peptide (p53p^AAA^) was used as the negative control. All FP data were fitted with Origin 2017 for obtaining *K*_*d*_ values.

### Isothermal titration calorimetry (ITC) assay

The protein−ligand interactions were characterized using a model ITC-200 isothermal titration microcalorimeter (Malvern, USA) at 25 °C. A typical experiment included the injection of 19 aliquots (2.0 μL each) containing an ~0.2 mM ligand solution into an ~10–20 μM protein solution in the ITC cell (volume of ∼200 μL). Titrations were performed with a stirring speed of 750 rpm and a spacing time of 120 s. A control experiment was run by injecting a ligand solution into buffer instead of a protein solution in the cell. Before data analysis, the control values were subtracted from the experimental data. The binding isotherms were integrated to give the enthalpy change (Δ*H*) plotted as a function of the molar ratio of the ligand. When necessary, prior to the integration procedures, the baseline was manually adjusted to minimize the background noise. The Origin 7.0-based software was used for data analysis with the one-set-of-binding-sites model. The disassociation constant of *K*_d_ was determined from the slope of the central linear part of the fractional saturation curve. The Gibbs free energy change (Δ*G*) and the entropy change (Δ*S*) were calculated on the basis of the following equations: Δ*G* =−*RT ln Kd* = Δ*H* − *T*Δ*S*, where Δ*H* was derived from the original Δ*H/*molar ratio plots.

### Synthesis of nutlin analogs

Synthesis of nutlin analogs was performed using the procedure developed by Davis et al.^52^ via the development of a diastereo- and enantioselective bisamidine-catalyzed aryl nitromethane addition to an azomethine, as described in Supplementary Note 1 and Supplementary Fig. 13. All reactions used 1.1 equivalent of nitroalkane in toluene (0.1 M) with an 18–26 h reaction time unless otherwise noted. The reaction was monitored by LC-MS until conversion was complete. The reaction was quenched with water. The aqueous layer was extracted with dichloromethane. The organic layers were combined, dried over sodium sulfate, and concentrated to give the crude product. Purification was performed on a Waters reverse-phase HPLC (C18 column, mobile phase: water with 0.1% formic acid and methanol with 0.1% formic acid) and was further separated by SFC (OD-H column) in order to give pure enantiomers.

### Molecular dynamics simulation

Molecular dynamics (MD) simulations were performed to investigate the conformational preference of H72. The initial structures of N-MdmX and N-Mdm2 were isolated from the N-MdmX/p53p (3DAB) and N-Mdm2/p53p complex (1YCR), respectively.

We performed standard MD simulations with the AMBER99SB-ILDN force field^53^ and TIP3P^54^ water model using GROMACS^55,56^. One N-MdmX or N-Mdm2 molecule was placed in cubic box with periodic boundary conditions. Counter ions were added to neutralize the net charges. All bonds were constrained using the LINCS algorism^57^. A time step of 2 fs was used. Long-range electrostatic interactions were calculated by particle-mesh Ewald method^58^ with a fourth-order interpolation and a grid spacing of 0.16 nm. The cutoff distances were set to 10 Å for short-range electrostatic and van der Waals interactions. The solute and solvent were coupled separately to a temperature bath of 298 K using a velocity-rescaling thermostat with a relaxation time of 0.1 ps^59^. The pressure was maintained at 1 bar using the Parrinello–Rahman algorithm^60^ with a relaxation time of 2 ps and isothermal compressibility of 4.5 ×  10^−5^ bar^−1^. The systems were relaxed by 1000 steps of the steepest-descent energy minimization followed by 1 ns equilibration at NVT and NPT ensembles. Production simulations with 1 μs duration were carried out in the NVT ensemble. Coordinates were saved every 5 ps and the side chain dihedral angles were calculated.

### Molecular docking

Potent inhibitors were generated with ChemBioDraw14 and further energetically minimized with ChemBio3D (PerkinElmer, USA). The minimized structures were exported in pdb format and manually evaluated using the ADT tool program harboring the AutoDock 4 program^61^. The residues H72 and K93 around the ligand pocket on N-MdmX and N-Mdm2 were set up as flexible residues. The receptor grid box was set directly on the MdmX crystal structure obtained in this study after extraction of the included nutlin-3a molecule.

### Real-time RT-qPCR reaction

Cancer cells with wild-type p53 (HCT116, RKO, and H460a) were treated with H203 for 8 h, and the change in the level of transcription was measured by quantitative PCR and expressed as fold induction compared with the untreated control. Total RNAs were extracted using a Trizol protocol with RNeasy^®^ Mini kit from Qiagen (TX, USA). The concentration of RNAs was measured with Nanodrop-2000c. Real-time qPCR was performed with a BaldStar TaqMan One-Step RT-qPCR Kit from Biorab (Beijing, China) on a BioRad CFX96 qPCR instrument (CA, USA). The primers and probes for qPCR were designed using Primer Express 3.0 software (Applied Biosystems, USA). The sequences of primers for p21 were RT-p21-L1 (5′-CTTTGTCACCGAGACACCAC-3′) and p21-R1 (5′-CAGGTCCACATGGTCTTCCT-3′). The sequence of the probe for p21 was p21-P1 (5′-ACTCATCCCGGCCTCGCCGG-3′). The sequences of primers for p53 were RT-p53-L1 (5′-GTCCAGATGAAGCTCCCAGA-3′) and RT-p53-R1 (5′-CAAGAAGCCCAGACGGAAAC-3′). The sequence of the probe for p53 was p53-P1 (5′-AGCTCCTACACCGGCGGCCC-3′). TaqMan probes were labeled with 5′-FAM and 3′-TAMRA. All primers and probes were synthesized by GenScript (Nanjing, China). Samples were analyzed in triplicate and normalized to the control.

### Analysis of cell proliferation with MTT assay

MTT assay was performed on a Synergy H1 multiplate reader (Biotek, USA) using H1299 cells containing engineered-inducible wild-type p53 gene, encoding p53 protein tagged with GFP protein. Overexpression of Mdm2 and MdmX was achieved by transfecting pCMV plasmids harboring a gene encoding the full-length Mdm2 and MdmX tagged with RFP protein. Cells were grown at 37 °C with 5% CO_2_ in Dulbecco’s modified eagle medium supplemented with 10% serum, penicillin and streptomycin for 3 days, refreshed the medium with RPMI 1640 supplemented with 3% FBS. Cells were plated at a density of 1 × 10^4^/cm^2^. After 2 h, added H203 and nutlin-3a for 24 h. After adding 10 µl/well MTT (5 mg/ml) solution, the cells were incubated for another 4 h at 37 °C in a CO_2_ incubator. After the supernatant was discarded, the cells were washed with PBS, and 100 µl DMSO was added to each well. The plates were agitated on a plate shaker for 10 min and then were read the OD at 520 nm. Data were processed with MicroCal Origin software (v2017, MicroCal, USA).

### Western blotting

Total cellular proteins were extracted with a RPMI lysis buffer containing 50 mM Tris (pH 7.4), 150 mM NaCl, 1% Triton-X100, 0.1% sodium deoxycholate, 0.1% SDS and 0.1% PMSF. The concentration of the protein extracts was firstly measured with Nanodrop-2000c at OD_280nm_ and the cellular β-actin contents were then calibrated with mouse anti-β-actin monoclonal antibody from Proteintech (Wuhan, China; Cat#: HRP-60008; Gene ID 60, 100 μg/ml) with a dilution ratio of 1:10000. Calibrated samples were separated by SDS-PAGE, while a normal molecular marker was used. Each SDS-PAGE gel was electrically transferred to a polyvinylidene difluoride (PVDF) membrane (Millipore, USA). In order to minimize the usage of antibodies, each PVDF membrane was cut into different sections, based on the molecular weights of cellular p53, Mdm2, MdmX, p21, PUMA, and β-actin and the features of their corresponding antibodies provided by their manufacturers. Each section of PVDF membranes was blocked with a 5% skimmed milk powder dissolved in TBST buffer for 1 h at room temperature, followed by incubation individually with their corresponding primary antibodies for 2 h in a sealed plastic bag. After being washed three times with TBST, the membranes were incubated with HRP-conjugated secondary antibody for 1 hr in a sealed plastic bag. Multiple sections of PVDF membranes were collected and the protein bands were visualized using enhanced chemiluminescence (ECL) reagents from BioSharp (Beijing, China) on a Tanon 5200 Chemiliminescent Imager (Shanghai, China). Each blotting was repeated until a good quality image was achieved. Cellular p53 protein was blotted with a rabbit anti-TP53 polyclonal antibody IgG from CUSABIO (TX, USA; Cat#: CSB-PA15509AORB, Lot#: F0912A) with a dilution ratio of 1:4000; Cellular Mdm2 and MdmX proteins tagged with RFP were assayed with a mouse anti-RFP monoclonal antibody from Solarbio (Beijing, China; Cat#: K20016M) with a dilution ratio of 1:10,000. Cellular p21 protein was detected with a rabbit anti-p21 polyclonal antibody from Elabscience (Wuhan, China; Cat#: E-AB-40097) with a dilution ratio of 1:500. Cellular PUMA protein was detected with a rabbit anti-human PUMA monoclonal antibody from Beyotime (Shanghai, China; Cat#: AF1204; Gene ID 27113) with a dilution ratio of 1:1000; Secondary antibodies were detected with either HRP-conjugated goat anti-rabbit IgG(H + L) which was from Proteintech (Wuhan, China; Cat#: SA00001-2) with a dilution ratio of 1:10000 or HRP-conjugated goat anti-mouse IgG(H + L) which was form Biosharp (Guangzhou, China; Cat#: BL001A, 0.8 mg/ml) with a dilution ratio of 1:10,000, respectively.

### Reporting summary

Further information on research design is available in the Nature Research Reporting Summary linked to this article.

## Supplementary information

Supplementary information

Reporting Summary

## Data Availability

The crystal structures generated in this study have been deposited in the Protein Data Bank under accession codes 7C3Y (N-MdmX/nutlin-3a), 7C44 (N-MdmX/nutlin-3a), 7C3Q (N-MdmX/nutlin-3a), 5ZXF (N-Mdm2/nutlin-3a), 5ZO2 (N-MdmX/nutlin-3a), and 7EL4 (N-MdmX/p53p analog). The initial structure model for Mdm2 was adopted from a crystal structure of N-Mdm2/nutlin-3a, available under accession code 4J3E. The initial structure models for MdmX were the crystal structures of N-MdmX/Cpd15 and N-MdmX/p53 analog, available under accession codes 6Q9W and 6V4F. The crystal structures of the MdmX/p53 peptide complexes and the MdmX/WK298 complexes used for structure comparison are available under accession codes 3DAB and 3LBJ, respectively. The initial structure of N-Mdm2 for MD simulations was isolated from the N-Mdm2/p53p complexes, which is available under accession code 1YCR. Source data are provided with this paper.

## Source data

Source Data
