# Supplementary material for: Leveraging the multivalent p53 peptide-MdmX interaction to guide the improvement of small molecule inhibitors
Source: Nat Commun. 2022 Feb 28;13:1087. doi: 10.1038/s41467-022-28721-x (PMC8885691; doi:10.1038/s41467-022-28721-x)
Supplement: Supplementary file 1 — Supplementary information [file 41467_2022_28721_MOESM1_ESM.pdf]

Supplementary information

Leveraging the multivalent p53 peptide-MdmX interaction to guide the improvement of small molecule inhibitors

Xiyao Cheng[1], Rong Chen[1], Ting Zhou[1], Bailing Zhang[1], Zichun Li[1], Meng Gao[1], Yongqi Huang[1,], Huili Liu[2,*] and Zhengding Su[1,*]*

[1]Protein Engineering and Biopharmaceutical Sciences Laboratory, Hubei University of Technology, Wuhan, 430068, China;

[2]National Center for Magnetic Resonance in Wuhan, State Key Laboratory of Magnetic Resonance and Atomic and Molecular Physics, Wuhan Institute of Physics and Mathematics, Chinese Academy of Sciences,33 Hongshanche Road, Wuhan, Hubei, 430071, China

***To whom correspondence may be addressed**: ZDS, zhengdingsu@hbut.edu.cn or HLL, liuhuili@wipm.ac.cn or YQH, yqhuang@hbut.edu.cn

Supplementary Note 1

All reactions used 1.1 equivalent of nitroalkane in toluene (0.1 M) with an 18 − 26 h reaction time unless otherwise noted. The reaction was monitored by LC-MS until conversion was complete. The reaction was quenched with water. The aqueous layer was extracted with dichloromethane. The organic layers were combined, dried over sodium sulfate, and concentrated to give the crude product. Purification was performed on a Waters reverse-phase HPLC (C18 column, mobile phase: water with 0.1% formic acid and methanol with 0.1% formic acid) and was further separated by SFC (OD-H column) in order to give pure enantiomers.

8-((4S,5R)-4,5-bis(4-chlorophenyl)-1-(3-oxopiperazine-1-carbonyl)-4,5-dihydro-1H-imidazol-2-yl)-3,4-dihydro-1H-benzo[e][1,4]diazepine-2,5-dione （H202 ）

Boc_2O (765 mg, 3.5 mmol) was added at 0° C to a solution of **1** (1.01 g, 3.6 mmol) in DCM (20 mL). The mixture was stirred for 2 h, and then quenched with water (15 mL). The two phases were separated and the aqueous phase was further extracted with DCM twice. The combined organic phases were washed with water (15 mL), dried over anhydrous Na_2SO4 and concentrated to obtain **2** (1.32 g, 96% yield) as white solid, which was used in the next step without further purification.

L-mandelic acid (546 mg, 3.6 mmol) was added to a solution of **2** (2.61 g, 6.9 mmol) in THF (52 mL) at room temperature. The mixture was stirred for 30 min at 70° C, and then stirred at room temperature for 1 h and 0° C for 30 min. After filtration, the filtration cake was collected (1.25g, 90% ee) and further crystallized from THF (22 mL) to give Cpd 4-salt (918 mg, > 99% ee) as white crystal which was freed from saturated aq. Na_2CO_3 to give **3** (623 mg, yield 25%) as white solid.

To a solution of 2,5-Dioxo-2,3,4,5-tetrahydro-1H-benzo [1,4]diazepine-8-carboxylic acid（**4**）(950 μmol) in DCM (15ml) were added DIPEA (238 mg, 1.84 mmol) and HATU (1.05 g, 2.76 mmol) at 0 °C. The mixture was stirred at 0 °C for 30 min and then **3** (351 mg, 921 μmol) was added. The reaction mixture was stirred at room temperature overnight. Water （20 ml）was added and the resulting mixtures was extracted with DCM three times (30 ml x3). The combined organic phases were dried over anhydrous Na_2SO_4 and concentrated. The residue was purified by flash column chromatography on silica gel (DCM: MeOH=5:1) to afford **5**. To a solution **5** (802 μmol) in DCM (8 mL), HCO_2H (1.00 g, 8.77 mmol) was added. The reaction mixture was stirred at room temperature for 3 h and then concentrated. The residue was poured into water (50 mL) and the resulting mixture was adjusted to pH 9. This mixture was then extracted with DCM (15 mL x 2) and the combined organic phases were washed with brine (10 mL), dried over Na_2SO_4 and concentrated to afford yellow oil intermediates (91% yield) as, which was used in next step without further purification.

To a solution of the yellow oil intermediates (230 μmol) in DCM (8 mL), CDI (68 mg, 425 μmol) was added at 0° C. The mixture was stirred at room temperature for 1 h. Piperazin-2-one (42 mg, 420 μmol) was added at room temperature and the mixture was stirred at room temperature for 2 h. Water (10 mL) was added and the 20 resulting mixture was extracted with EtOAc three times (10 mL/each). The combined organic phases were washed with brine (10 mL), dried over anhydrous Na_2SO_4 and concentrated to afford white solid intermediates.

To a solution of Ph_3PO (120 mg, 432 μmol) in DCM (6 mL), Tf_2O (240 mg, 850 μmol) was added dropwise at 0° C. The mixture was stirred at 0° C for 30 min. A solution of the above white solid intermediates (225 μmol) in DCM (2 mL) was added dropwise at 0° C. The mixture was

stirred at 0° C for 1 h. The reaction mixture was concentrated and the residue was purified with prep-TLC (DCM : MeOH = 11: 1) to afford H202 (62% yield) as white solid. ^{1}H NMR (600 MHz, DMSO): δ 10.45 (26NH), 8.49 (s, 22H), 8.08 (d, J = 7.5 Hz, 25H), 8.05 (29NH), 8.01 (37NH), 7.79 (d, J = 7.5 Hz, 19H), 7.23 (d, J = 7.5 Hz, 14H), 7.23 (d, J = 7.5 Hz, 16H), 7.18 (d, J = 7.5 Hz, 13H), 7.18 (d, J = 7.5 Hz, 17H), 7.09 (d, J = 7.5 Hz, 9H), 7.09(d, J = 7.5 Hz, 11H), 6.99 (d, J = 7.5 Hz, 8H), 6.99 (d, J = 7.5 Hz, 12H), 5.85 (d, J = 7.0 Hz, 1H), 5.37 (d, J = 7.0 Hz, 2H), 3.98 (s, 28H), 3.87 (s, 39H), 3.64 (t, J = 7.1 Hz, 35H), 3.64 (q, J = 7.1 Hz, 36H); ^{13}C NMR (600 MHz, DMSO): δ 168.28, 168.04, 165.83, 161.63, 155.81, 139.18, 137.13, 136.55, 136.22, 132.45, 132.14, 130.70, 129.53, 128.88, 128.33, 128.29, 127.64, 124.01, 120.99, 61.73, 56.72, 48.12, 44.87, 43.85, 36.72; MS (m/z): [M]+ calcd. for C29H24Cl2N6O4, 591.40 Da; found, 591.40 Da.

8-((4S,5R)-4,5-bis(4-chlorophenyl)-1-(3-oxopiperazine-1-carbonyl)-4,5-dihydro-1H-imidazol-2-yl)-1-phenyl-3,4-dihydro-1H-benzo[e][1,4]diazepine-2,5-dione（H203）

To a solution of 2,5-Dioxo-2,3,4,5-tetrahydro-1H-benzo [1,4] diazepine-8-carboxylic acid (**6**) (950 μmol) in DCM (15ml) were added DIPEA (238 mg, 1.84 mmol) and HATU (1.05 g, 2.76 mmol) at 0 °C. The mixture was stirred at 0 °C for 30 min and then **3** (351 mg, 921 μmol) was added. The reaction mixture was stirred at room temperature overnight. Water （20 ml） was added and the resulting mixtures was extracted with DCM three times (30 ml x3). The combined organic phases were dried over anhydrous Na_2SO_4 and concentrated. The residue was purified by flash column chromatography on silica gel (DCM: MeOH=5:1) to afford **7**. To a solution **7** (802 μmol) in DCM (8 mL), HCO_2H (1.00 g, 8.77 mmol) was added. The reaction mixture was stirred at room temperature for 3 h and then concentrated. The residue was poured into water (50 mL) and the resulting mixture was adjusted to pH 9. This mixture was then extracted twice with DCM (15

mL/each) and the combined organic phases were washed with brine (10 mL), dried over Na_2SO_4 and concentrated to afford yellow oil intermediates (93% yield) as, which was used in next step without further purification.

To a solution of the yellow oil intermediates (230 μmol) in DCM (8 mL), CDI (68 mg, 425 μmol) was added at 0° C. The mixture was stirred at room temperature for 1 h. Piperazin-2-one (42 mg, 420 μmol) was added at room temperature and the mixture was stirred at room temperature for 2 h. Water (10 mL) was added and the 20 resulting mixture was extracted with EtOAc three times (10 mL/each). The combined organic phases were washed with brine (10 mL), dried over anhydrous Na_2SO_4 and concentrated to afford white solid intermediates. To a solution of Ph_3PO (120 mg, 432 μmol) in DCM (6 mL), Tf_2O (240 mg, 850 μmol) was added dropwise at 0° C. The mixture was stirred at 0° C for 30 min. A solution of the above white solid intermediates (250 μmol) in DCM (2 mL) was added dropwise at 0° C. The mixture was stirred at 0° C for 1 h. The reaction mixture was concentrated and the residue was purified with prep-TLC (DCM : MeOH = 11: 1) to afford H203 (73% yield) as white solid. ^1H NMR (600 MHz, DMSO): δ 8.04 (27NH), 8.04 (s, 25H), 7.92 (d, J = 7.5 Hz, 22H), 7.90 (d, J = 7.5 Hz, 21H), 7.76 (38NH), 7.45 (t, J = 7.5 Hz, 44H), 7.45 (t, J = 7.5 Hz, 46H), 7.34 (t, J = 7.5 Hz, 45H), 7.26 (d, J = 7.5 Hz, 12H), 7.26 (d, J = 7.5 Hz, 14H), 7.25 (d, J = 7.5 Hz, 11H), 7.25 (d, J = 7.5 Hz, 15H), 7.13 (d, J = 7.5 Hz, 2H), 7.13(d, J = 7.5 Hz, 6H), 7.09 (d, J = 7.5 Hz, 43H), 7.09(d, J = 7.5 Hz, 47H), 6.94 (d, J = 7.5 Hz, 3H), 6.94 (d, J = 7.5 Hz, 5H), 5.74 (d, J = 7.0 Hz, 7H), 5.62 (d, J = 7.0 Hz, 8H), 4.40 (s, 28H), 3.45 (q, J = 7.1 Hz, 39H), 3.45 (t, J = 7.1 Hz, 40H), 3.41 (s, 36H); ^{13}C NMR (600 MHz, DMSO): δ 169.24, 167.81, 165.75, 162.35, 156.16, 141.23, 140.96, 137.14, 134.15, 132.33, 131.73, 131.11, 130.58, 129.94, 129.91, 129.02, 128.32, 128.23, 127.98, 125.01, 123.88, 68.23, 56.51, 48.72, 45.59, 42.13. MS (m/z): [M]+ calcd. for C35H28Cl2N6O4, 667.55; found, 670.60;

Supplementary Table 1. Data collection and refinement statistics of N-MdmX in complex with nutlin-3a.

**PDB ID**	**7C3Y**	**7C44**	**7C3Q**
**Crystal sample**	378-3	378-4	381-1
**Data collection**			
Space group	$P4_32_12$	$P4_32_12$	$P4_32_12$
Cell dimensions (Å)	47.55, 47.55, 90.68	47.47, 47.47, 91.19	47.73, 47.73, 92.32
a, b, c, α, β, γ	90.00°, 90.00°, 90.00°	90.00°, 90.00°, 90.00°	90.00°, 90.00°, 90.00°
Resolution (Å)	90.676– 1.49	27.03– 1.65	92.32 – 1.49
R_{merge}	0.06	0.04	0.07
$<I/\sigma(I)>$	1.70 (at 1.63Å)	1.70 (at 1.63Å)	2.54 (at 1.81Å)
Completeness (%)	100	99.8	95.8
Redundancy	22.7	24.1	23.0
**Refinement**			
Resolution (Å)	42.15 – 1.63	25.61 – 1.65	42.43 – 1.80
No. reflections	11624	13154	10024
R_{work} / R_{free}	0.200/0.227	0.207/0.265	0.197/0.241
Number of atoms	1694	1695	1660
B-factors	32.4	34.7	31.1
R.m.s. deviations	0.0103/1.63	0.0118/1.698	1.605
Bond lengths (Å)	0.0103	0.0118	0.0104
Bond angles (°)	1.63	1.698	1.605

Supplementary Table 2. Data collection and refinement statistics of N-Mdm2 in complex with nutlin-3a.

**PDB ID**	**5ZXF**	**5ZO2**
**Crystal sample**	CXY-018	CR-106
**Data collection**		
Space group	$P2_12_12_1$	$P2_12_12_1$
Cell dimensions (Å)	42.58, 43.22, 53.85	42.5, 42.99, 54.53
a, b, c, α, β, γ	90.00°, 90.00°, 90.00°	90.00°, 90.00°, 90.00°
Resolution (Å)	33.398-1.144	33.52-1.53
R_{merge}	0.07	0.05
$\langle I/\sigma(I) \rangle$	2.13 (at 1.25Å)	2.01 (at 1.35Å)
Completeness (%)	98.8	99.6
Redundancy	11.7	12.4
**Refinement**		
Resolution (Å)	1.25	1.35
No. reflections	26267	21289
R_{work} / R_{free}	0.218/0.243	0.226/0.254
Number of atoms	797	802
B-factors	17.7	20.2
R.m.s. deviations	0.85/0.85	1.23/1.25
Bond lengths (Å)	0.85	1.23
Bond angles (°)	0.85	1.25

Supplementary Table 3. Data collection and refinement statistics of N-MdmX in complex with p53p^{F19}

**PDB ID**	**7EL4**
**Crystal sample**	CXY-516
**Data collection**	
Space group	$I4_1$
Cell dimensions (Å)	65.10, 65.10, 94.98
a, b, c, α, β, γ	90.00°, 90.00°, 90.00°
Resolution (Å)	53.695-2.109
R_{merge}	0.068
$<I/\sigma(I)>$	2.23 (at 2.10Å)
Completeness (%)	100%
Redundancy	12.9
**Refinement**	
Resolution (Å)	
No. reflections	2.11
R_{work} / R_{free}	10599
Number of atoms	0.225/0.224
B-factors	1617
R.m.s. deviations	51.9
Bond lengths (Å)	0.0092/1.715
Bond angles (°)	0.0090

Supplementary Table 4. **The summary of the thermodynamic parameters of N-MdmX titrated with nutlin-3a, p53p and p53p^{F19}**

**Protein**	**N-MdmX**		
**Ligand**	**p53p**	**nutlin-3a**	**p53p^{F19}**
K_d (μM)	0.36 ± 0.02	19.62 ± 3.42	0.33 ± 0.03
ΔH (kcal/mol)	-18.7 ± 0.89	-2.34 ± 1.98	-17.46 ± 0.91
$-T\Delta S$ (kcal/mol)	9.91 ± 0.27	-4.08 ± 1.24	8.64 ± 2.12
ΔG (kcal/mol)	-8.79 ± 1.12	-6.42 ± 3.24	-8.84 ± 1.23

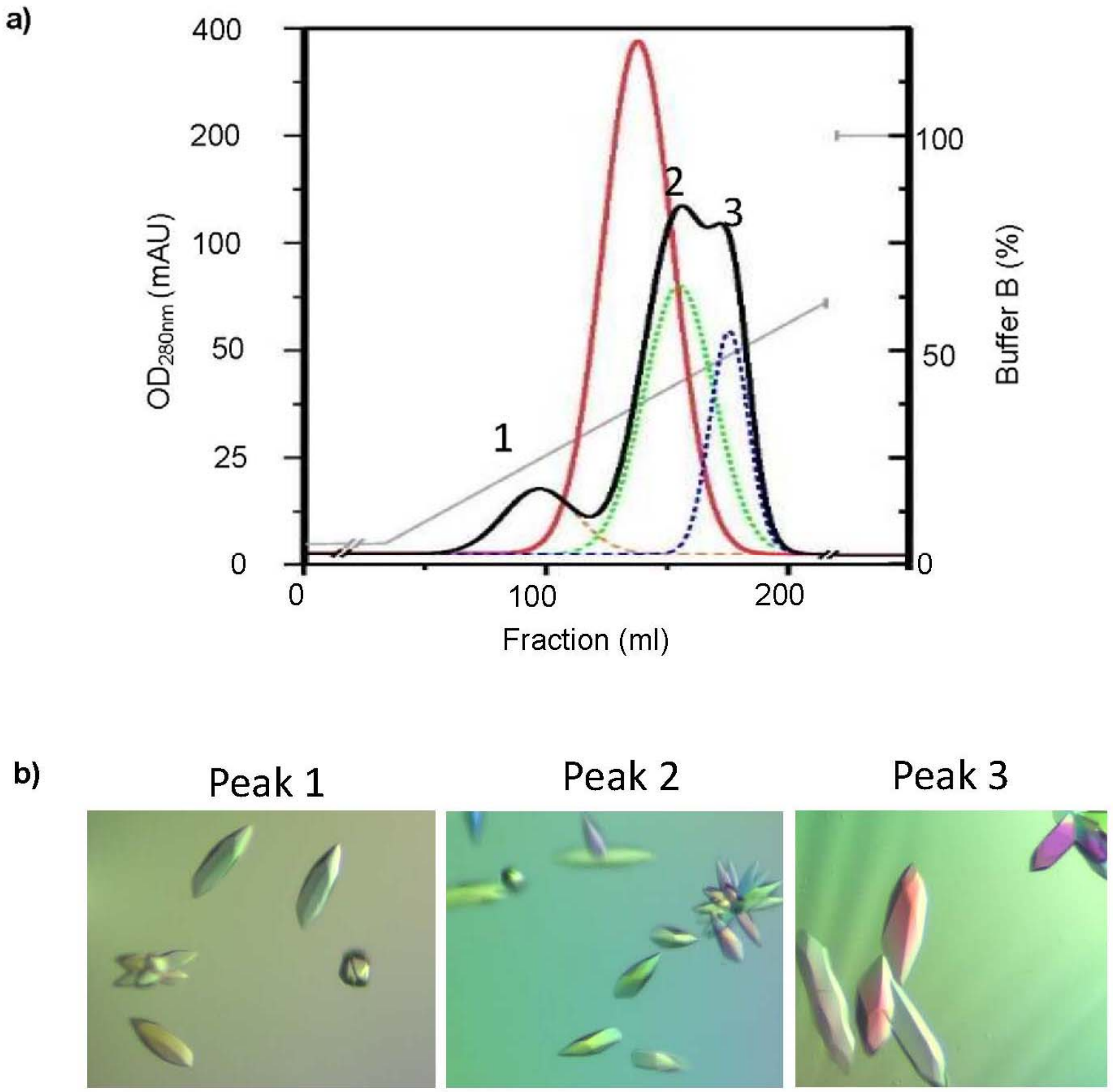

Supplementary Fig. 1. Purification and crystallization of N-MdmX. a) Purification of N-MdmX with Mono S cation ion-exchange in comparison with N-Mdm2. *Black*, N-MdmX; *red*, N-Mdm2; *grey*, NaCl gradient and *dash*, fitted peaks for N-MdmX profile. **b)** single protein crystals were obtained from the three peaks of protein fractions.

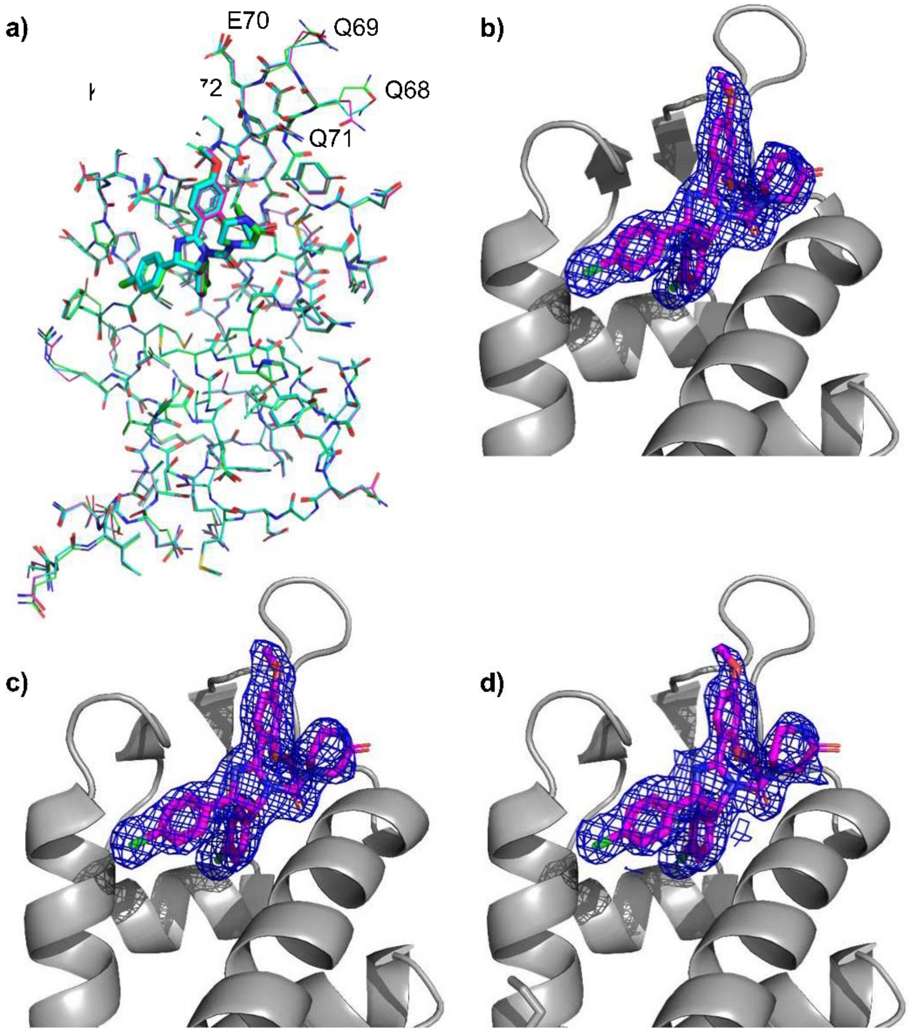
a)
E70
Q69
Q68
Q71
b)
c)
d)

e)

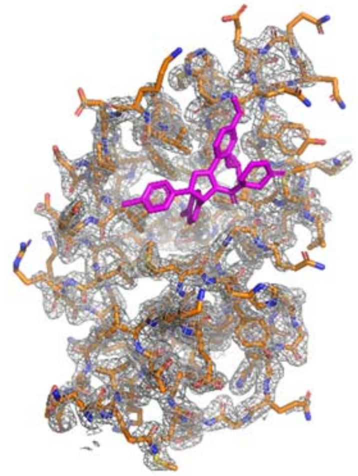

f)

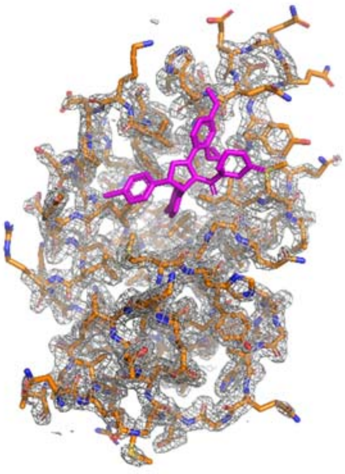

g)

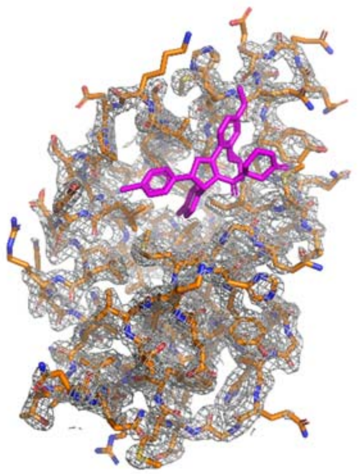

Supplementary Fig. 2. Interaction of nutlin-3a with N-MdmX. **a**) The side-chain configuration comparison of three crystal structures of N-MdmX in complex with nutlin-3a. *Blue*, *green*, and *magenta* represent the crystal structures obtained from Sample Peak 1, Sample Peak2 and Sample Peak 3, respectively. **b – d**) The 2Fo-Fc omit maps contoured at 1.0 σ of nutlin-3a molecules in the three crystal structures of N-MdmX/nutlin-3a complexes (7C44, 7C3Q and 7C3Y, respectively). **e-g**) The 2Fo-Fc omit maps contoured at 1.5 σ of N-MdmX in complex with nutlin-3a (7C44, 7C3Q and 7C3Y, respectively).

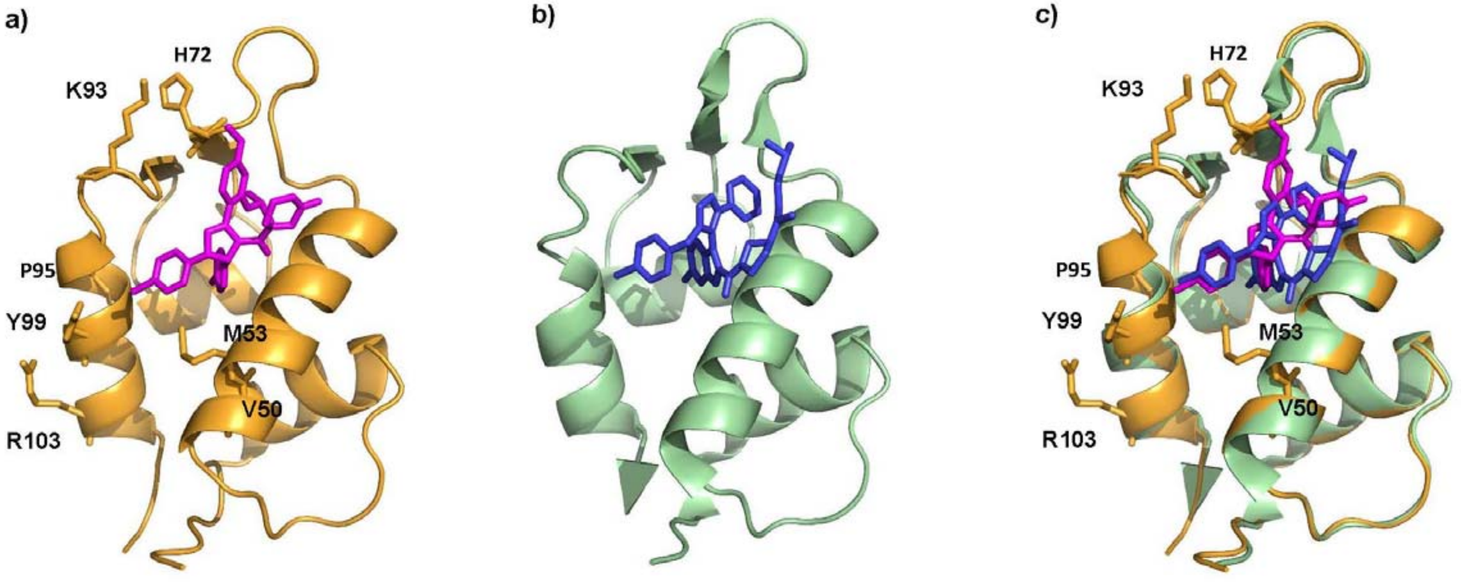

Supplementary Fig. 3. Comparison of N-MdmX structures in complex with nutlin-3a and WK298. The residues affecting ligand binding are depicted in stick. *Magenta*: nutlin-3a and *blue*: WK298. **a).** The N-MdmX/nutlin-3a complexes; **b).** The N-MdmX/WK298 complexes. **c).** Two N-MdmX structures are superimposed.

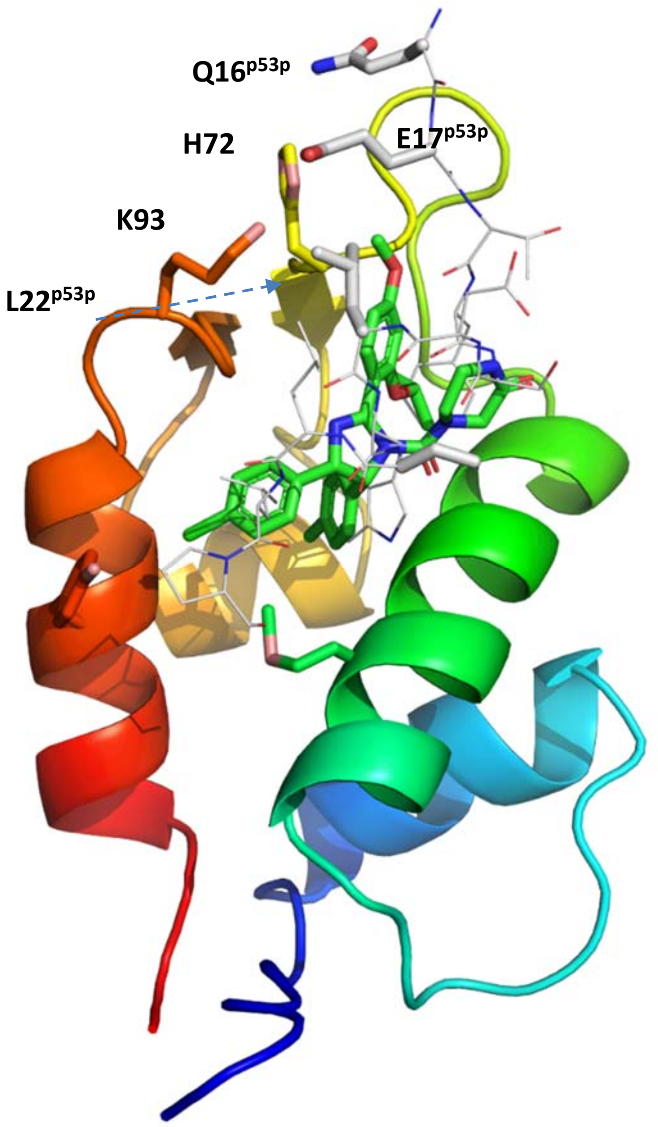

Supplementary Fig. 4. The additional residues from p53p, including Q16^{p53p}, E17^{p53p} and L22^{p53p}, formed intermolecular interactions with the R4-region of N-MdmX. P53p is depicted in line and the side-chains of Q16^{p53p}, E17^{p53p} and L22^{p53p} are shown in stick. Nutlin-3a is present in green stick.

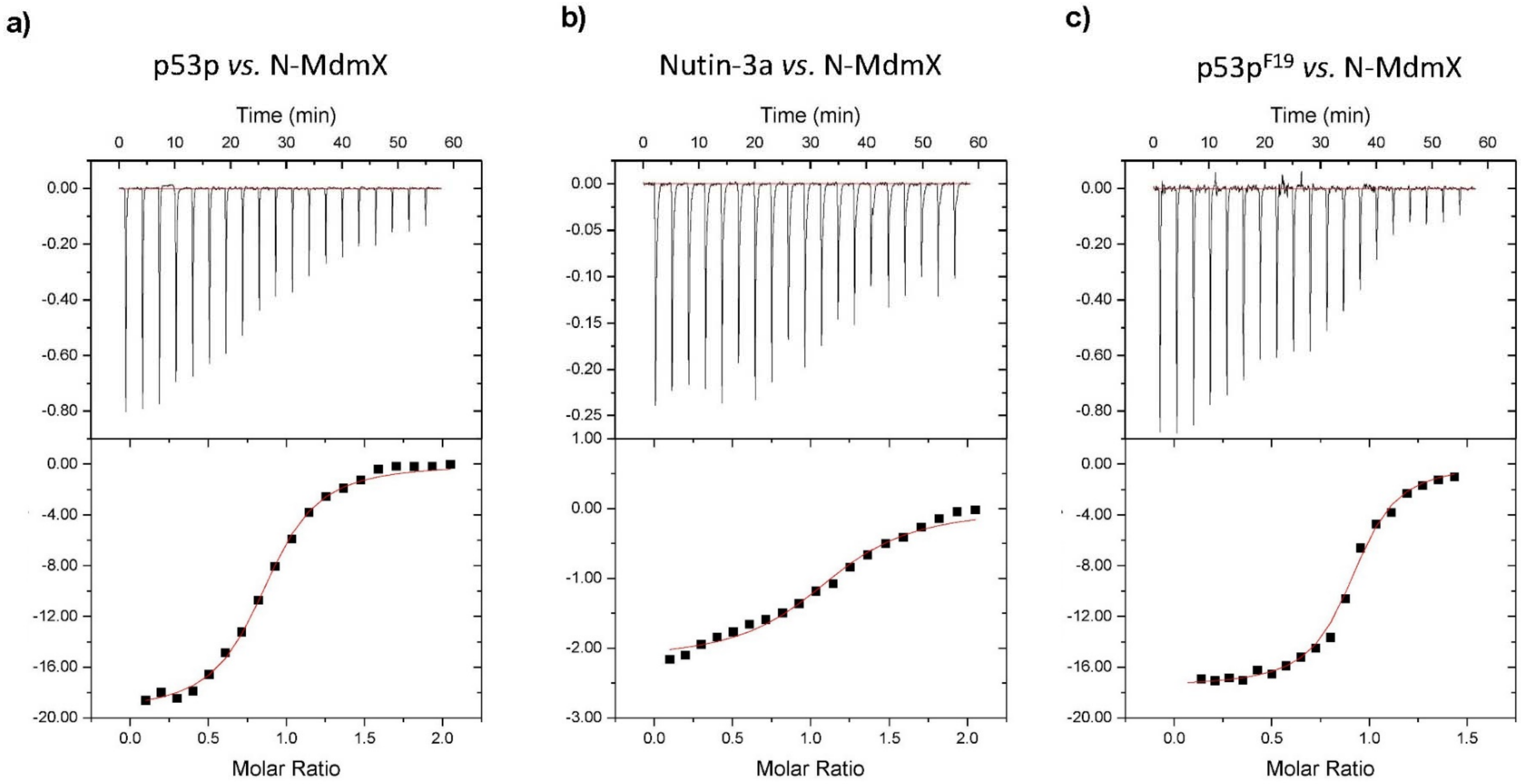

Supplementary Fig. 5. ITC assays of the interaction of N-MdmX with p53p, nutlin-3a and a p53p analog. Thermodynamic profiles describing the heat flow over the course of N-MdmX titrated with p53p (a), nutlin-3a (b) and the p53p analog, $p53p^{F19}$ (c) were fitted to a one-site binding model to obtain the values for stoichiometry (n), the binding constant (K_d) and enthalpy (ΔH) (Supplementary Table 4).

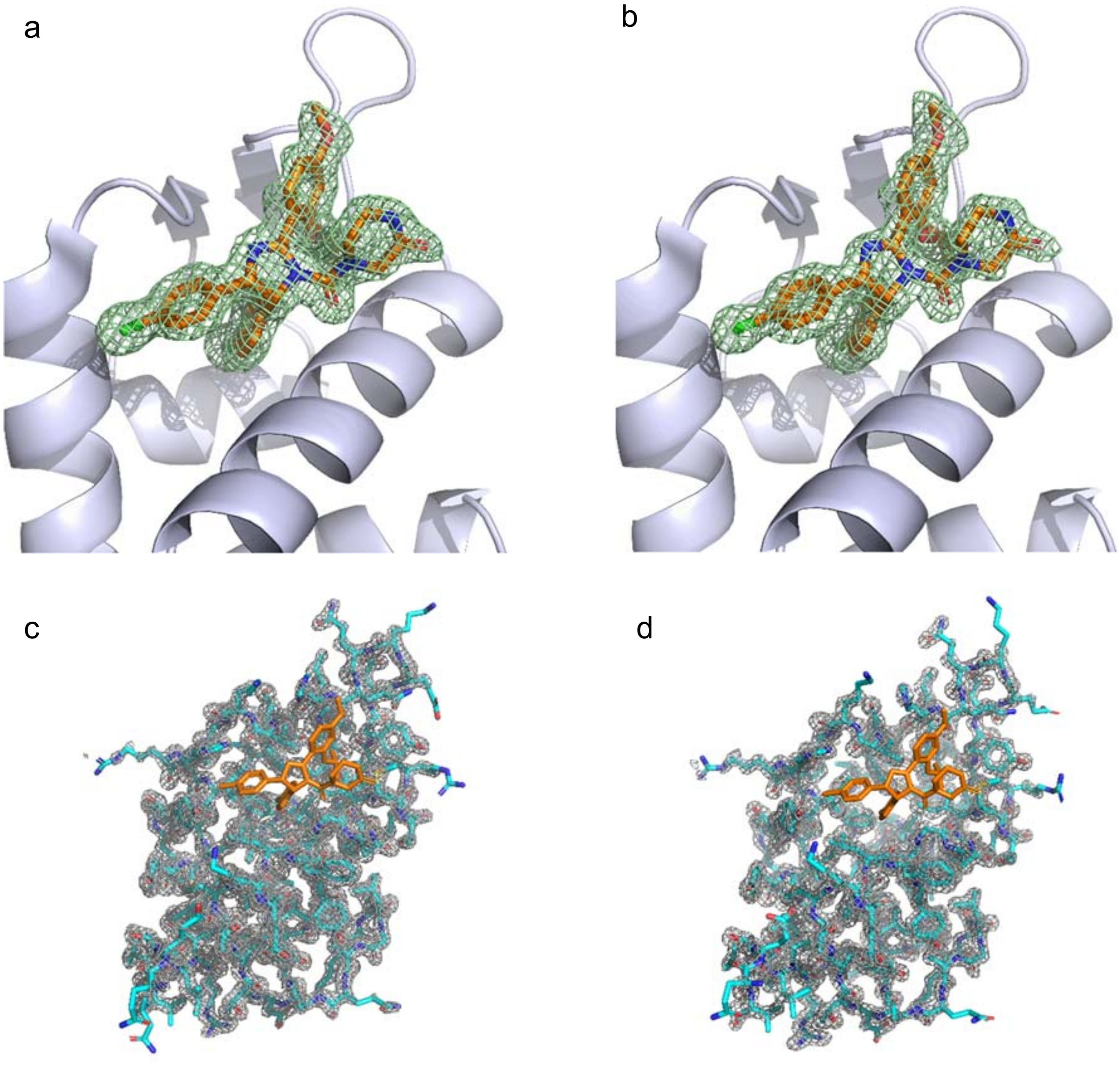

Supplementary Fig. 6. X-ray crystal structure of N-Mdm2 in complex with nutlin-3a. a-b) The 2Fo-Fc omit maps contoured at 1.0 σ of nutlin-3a molecules in the two crystal structures of N-Mdm2/nutlin-3a complexes (5Z02 and 5ZXF, respectively). **c-d)** The 2Fo-Fc omit maps contoured at 1.5 σ of N-MdmX2 in complex with nutlin-3a (5Z02 and 5ZXF, respectively).

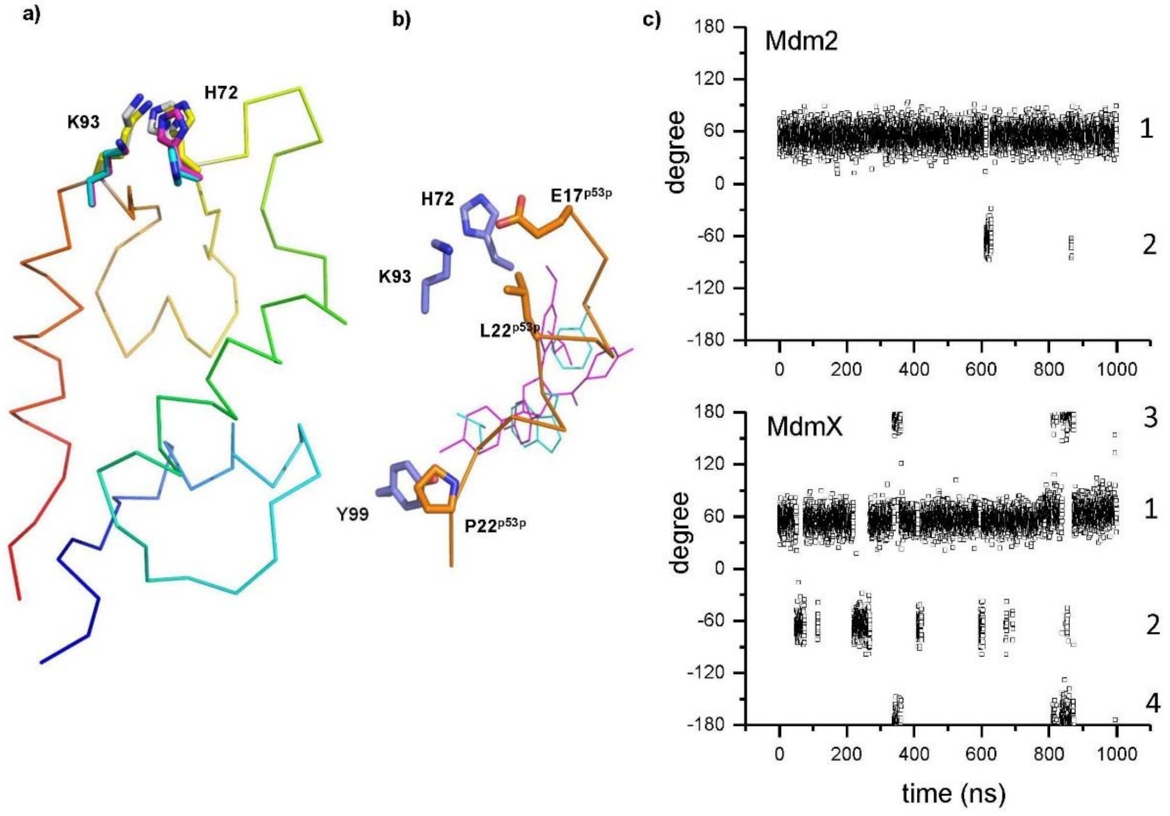

Supplementary Fig. 7. The interaction of K93-H72 pair with ligand is significantly correlated with the ligand binding affinity. a) Comparison of the configurations of H72 residues on N-MdmX and N-Mdm2 interacting with nutlin-3a and p53p. *Yellow*, MdmX/nutlin-3a complexes; *grey*, MdmX/p53p complexes; *cyan*, Mdm2/nutlin-3a complexes in close state and *magenta*, Mdm2/nutlin-3a complexes in open state. **b**) E17^{p53p} and L22^{p53p} formed an intermolecular interaction with H72 and K93 from N-MdmX. P27^{p53p} formed an intermolecular interaction with Y99 from N-MdmX. **c**) Molecular dynamics simulation of the side-chain configuration of H72 on N-MdmX in comparison with N-Mdm2.1, the unbound state; 2, the bound state; 3 and 4, other states. It was notable that the 1 and 2 states in the N-Mdm2/nutlin-3a complexes were observable in X-ray crystal structures. Source data for **c** are provided as a Source Data file.

a)

b)

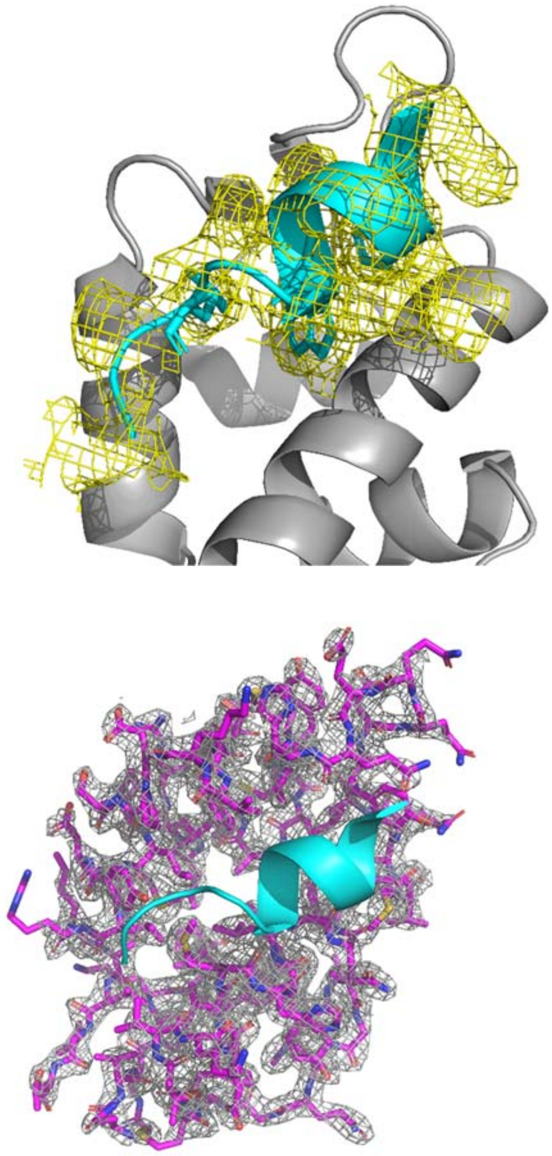

Supplementary Fig. 8. X-ray crystal structure of N-MdmX in complex with p53p^{F19} (7el4.pdb**). a)** The 2Fo-Fc omit maps contoured at 1.0 σ of p53p^{F19} peptide in the crystal structure. **b)** The 2Fo-Fc omit maps contoured at 1.5 σ of N-MdmX in the complexes.

a)

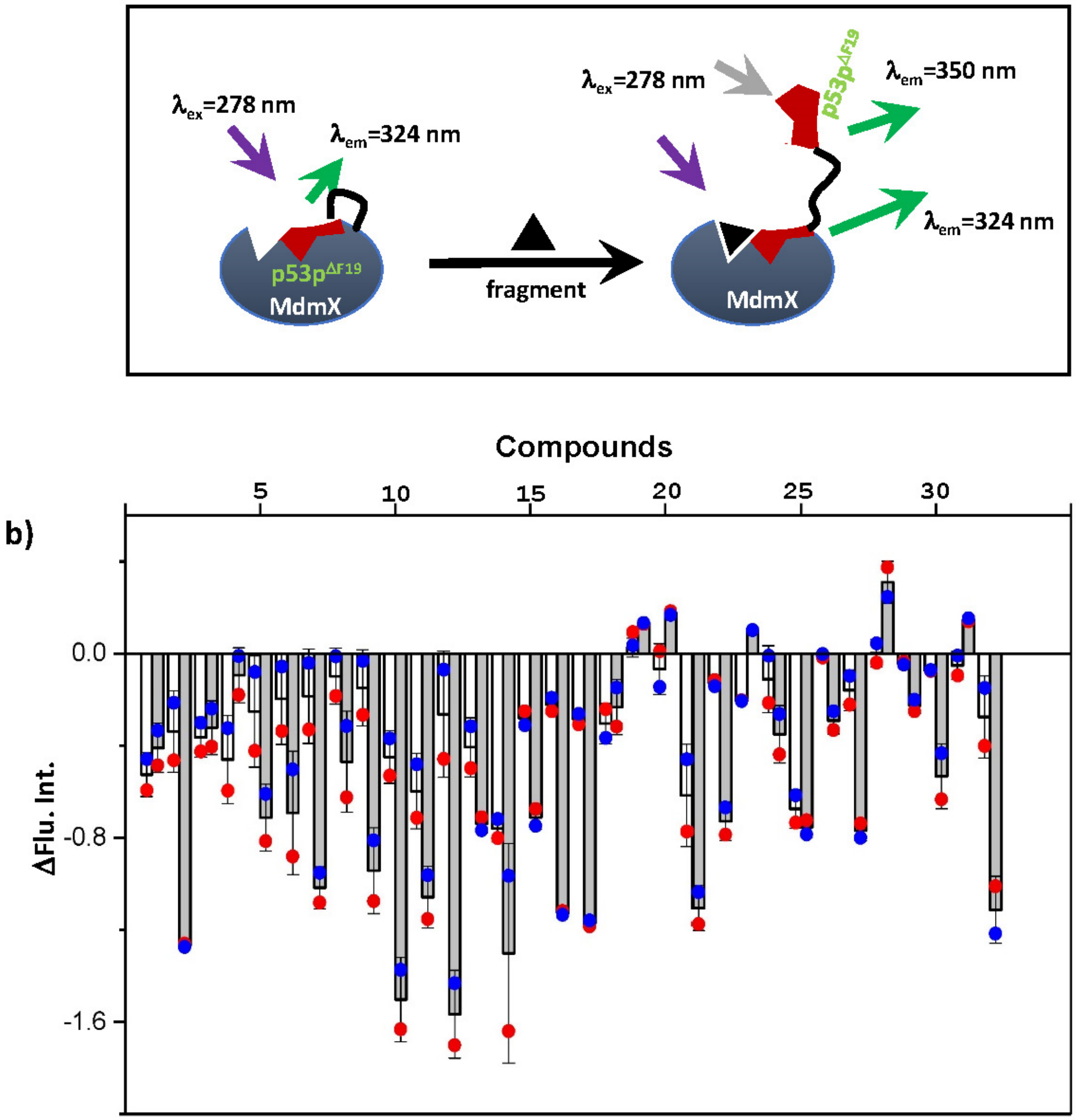

Supplementary Fig. 9. HTS of a Mdm2 inhibitor library using with the p53p$^{\Delta F19}$-MdmX fusion protein. **a**) A cartoon was given to interpret the fluorescent signal changes of W23^{p53p} responding the binding of allosteric fragments to the F19^{p53p} subsite on the N-MdmX. the maximum emission wavelength of the W23 fluorescent signal in bound state is at 324 nm, when an allosteric fragment binds to the F19^{p53p} subsite, the W23 fluorescent signal is enhanced. Otherwise, the W23 fluorescent signal is attenuated and its maximum wavelength is moved to 350

nm, as a fragment displaces $p53p^{\Delta F19}$. **b**) The W23 fluorescent signal changes were caused by 1 μM (open) and 10 μM (fill) of each compound. Each set of data was averaged from two-time screening assays and are presented as mean values ±SD. n = 2 independent screening experiments. Source data for **b** are provided as a Source Data file.

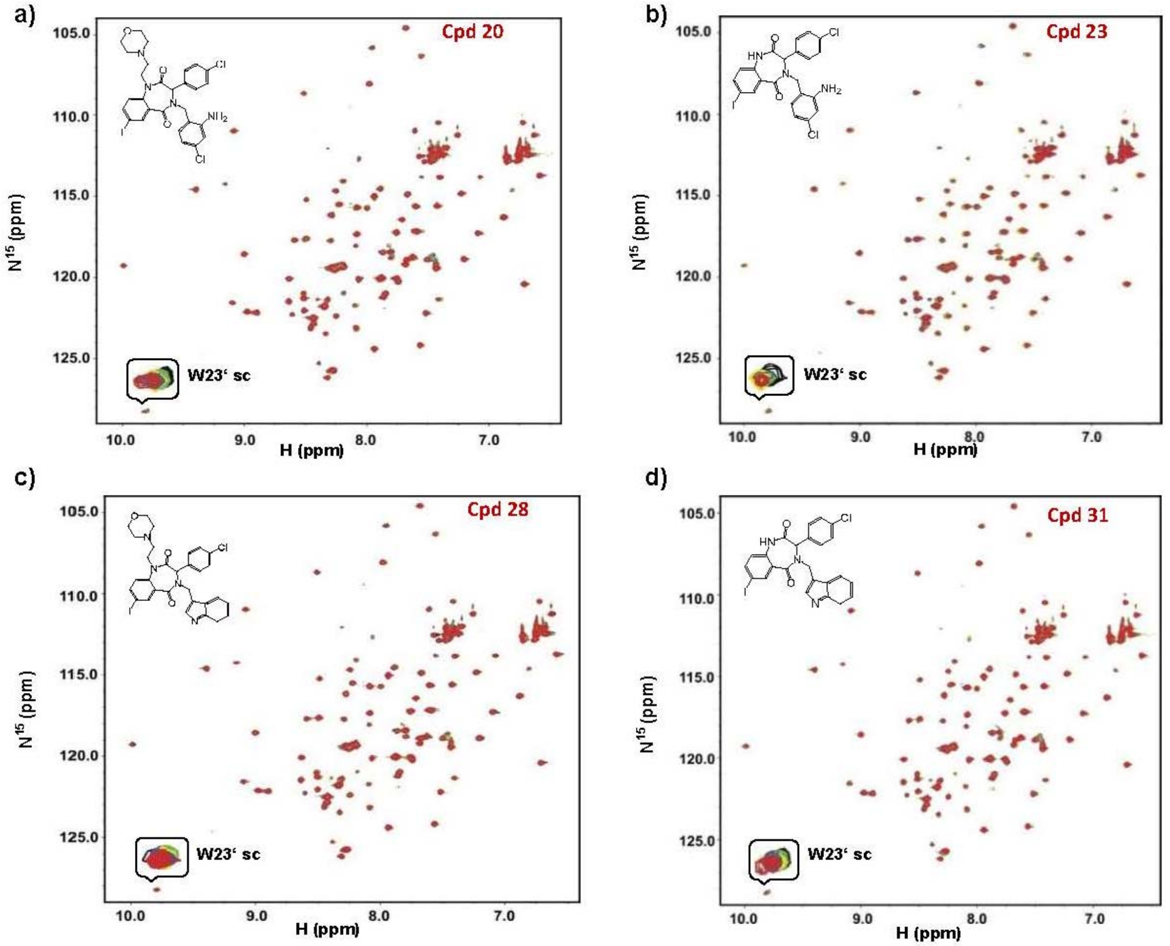

Supplementary Fig. 10. ^{15}N-^{1}H HSQC NMR titration of N-MdmX byCpd20 (a), Cpd23 (b), Cpd26 (c) and Cpd31 (d). The structures of four compounds were included in the inset. The

resonance peaks of W23 side-chains were enlarged for clarity (see **Fig. 4f** in Main text). The molar ratio of protein and compound was coded in color. *Black*, 1:0; *green*, 1:0.5; *blue*, 1:1; *yellow*, 1:1.5 and *red*, 1:2.

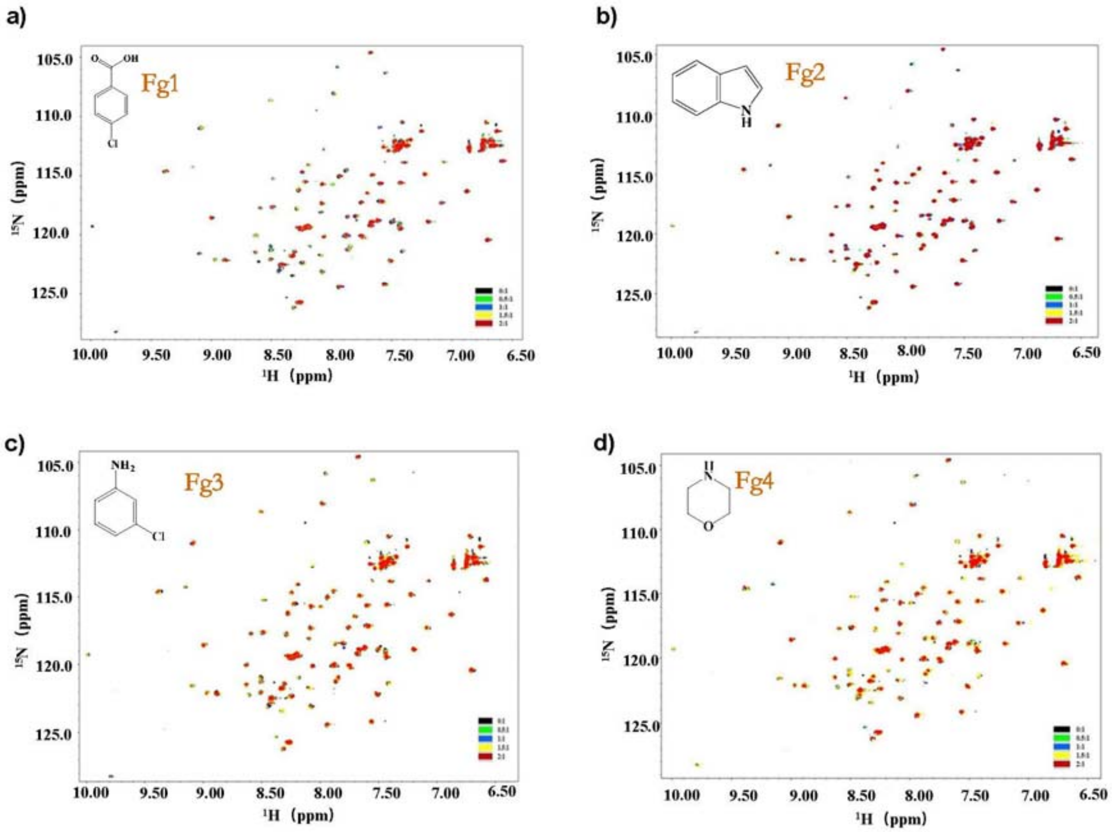

Supplementary Fig. 11. ^{15}N-^{1}H HSQC NMR titration of N-MdmX by Fg1 (a), Fg2 (b), Fg3 (c) and Fg4 (d). The structures of four fragments were included in the inset. The resonance peaks of W23 side-chains were enlarged for clarity in **Fig. 3g**). The molar ratio of protein and compound was coded in color. *Black*, 1:0; *green*, 1:0.5; *blue*, 1:1; *yellow*, 1:1.5 and *red*, 1:2.

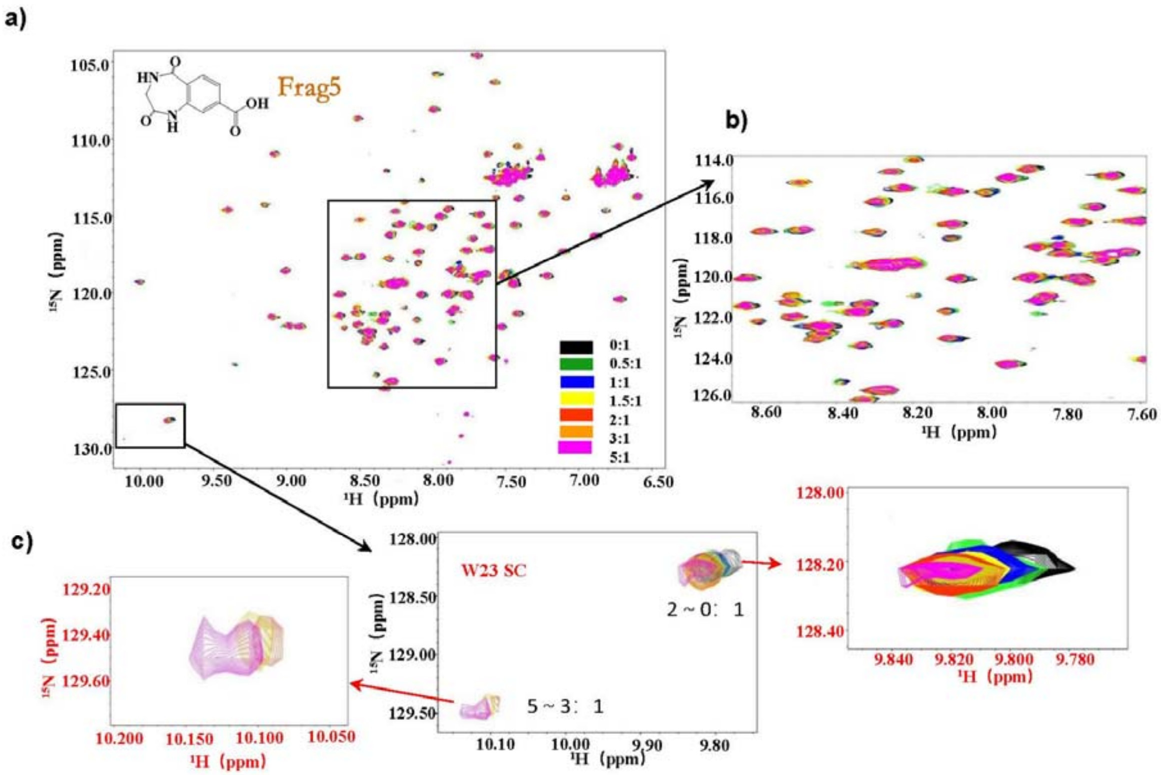

Supplementary Fig. 12. ^{15}N-^{1}H HSQC NMR titration of N-MdmX by Fg5. The structure of Fg5 was included in the inset. The molar ratio of protein and compound was coded in color. *Black*, 1:0; *green*, 1:0.5; *blue*, 1:1; *yellow*, 1:1.5; *red*, 1:2, *orange*, 1:3 and *pink*, 1:3. **a).** A full ^{15}N-^{1}H HSQC NMR titration spectrum. **b**) An enlarged local spectrum for the resonances in the range between 114 ppm and 126 ppm in N^{15}-dimension and between 7.5 ppm and 9.0 ppm in H-

dimension. were. **c**) The resonance peaks of W23 side-chains were enlarged for clarity (see **Fig. 3h)**.

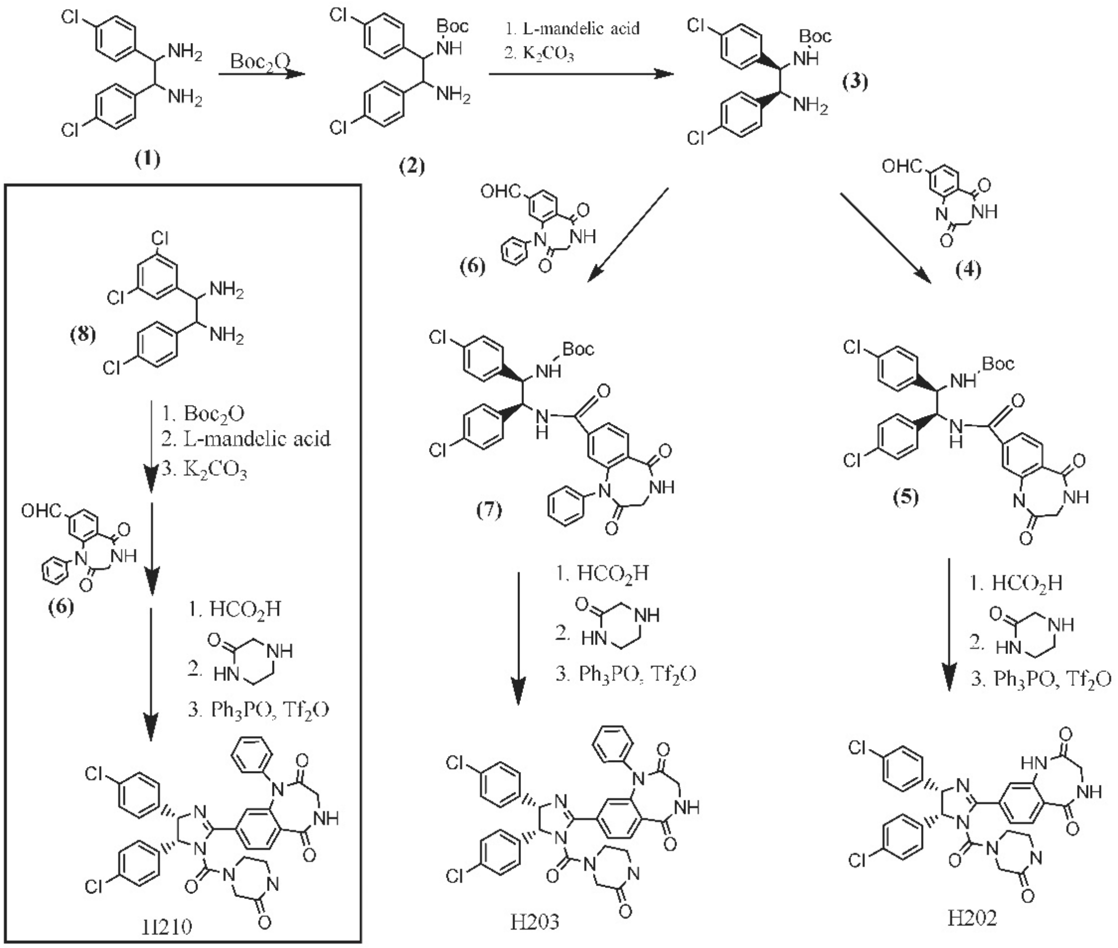

Supplementary Fig. 13. A scheme outlining synthesis of nutlin analogs.

a

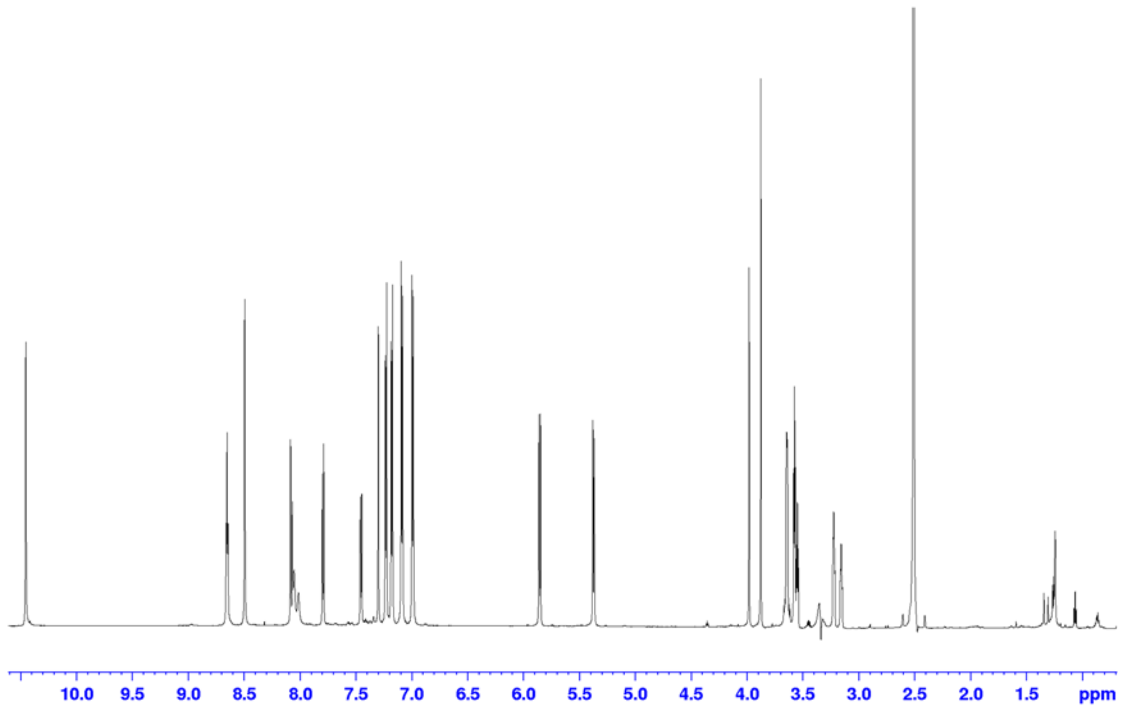
10.0 9.5 9.0 8.5 8.0 7.5 7.0 6.5 6.0 5.5 5.0 4.5 4.0 3.5 3.0 2.5 2.0 1.5 ppm

b

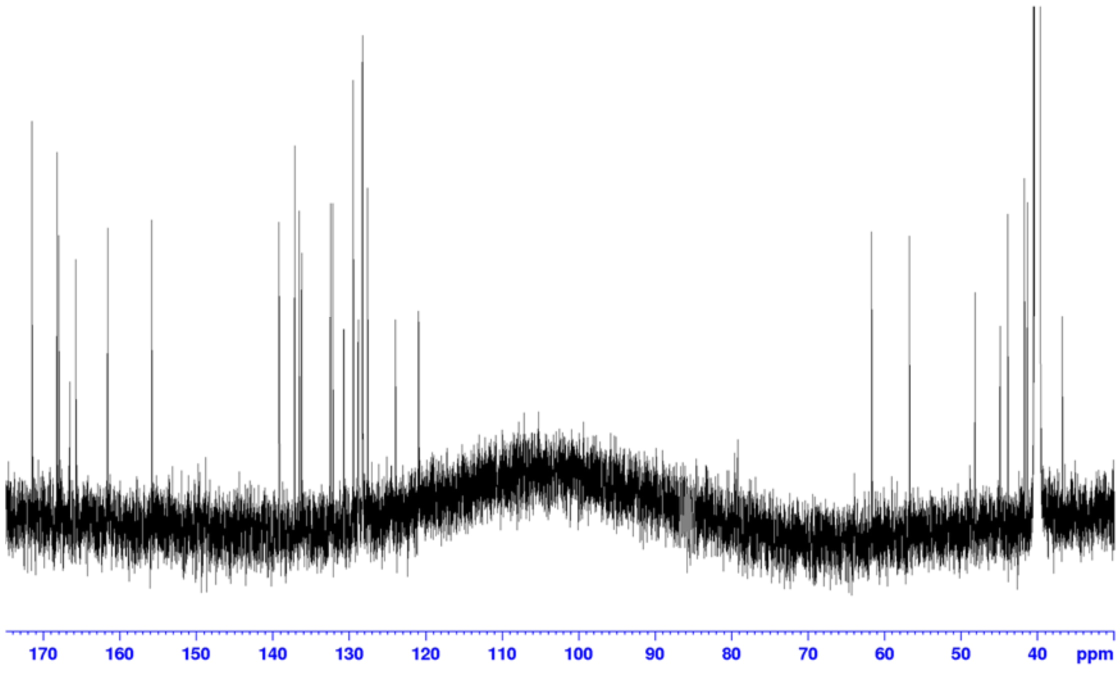
170 160 150 140 130 120 110 100 90 80 70 60 50 40 ppm

c

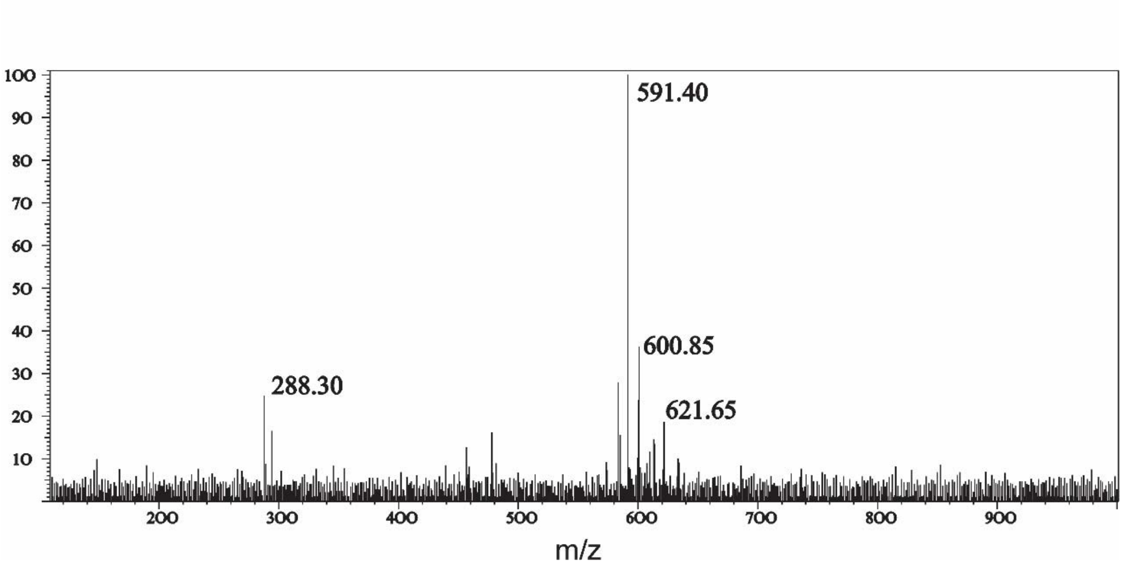

Supplementary Fig. 14. NMR and MS characterization of H202. a) & b). ^{1}H and ^{13}C NMR spectra of H202. **c**) Mass spectrum of H202.

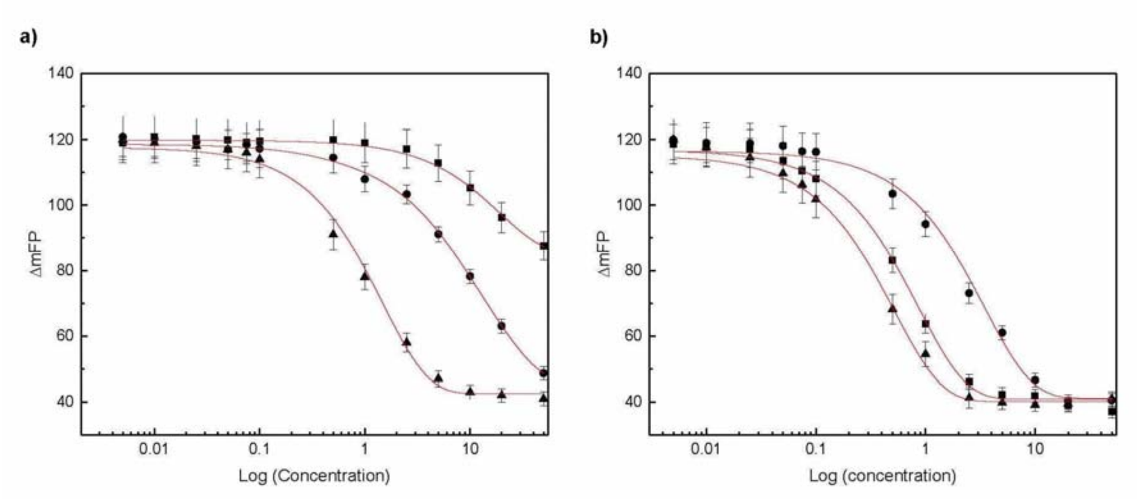

Supplementary Fig. 15. Determination of the inhibitory constants of nutlin-3a, H202 and H203 to Mdm2 and MdmX analyzed by fluorescence polarization. *Square*, nutlin-3a; *cycle*, H202 and *triangle*, H203. n = 3 independent experiments and the data are represented as mean values ± SD. **a**) N-MdmX; **b**) N-Mdm2. Source data are provided as a Source Data file.

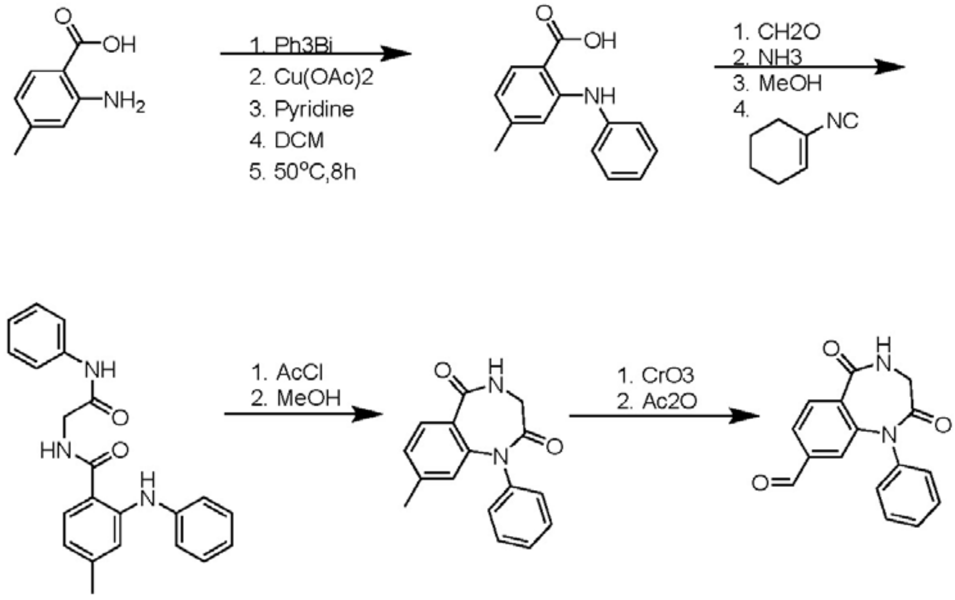

Supplementary Fig. 16. Synthesis of 2,5-dioxo-1-phenyl-2,3,4,5-tetrahydro-1H-benzo-[1,4]diazepine-8-carboxylic acid （6）.

a

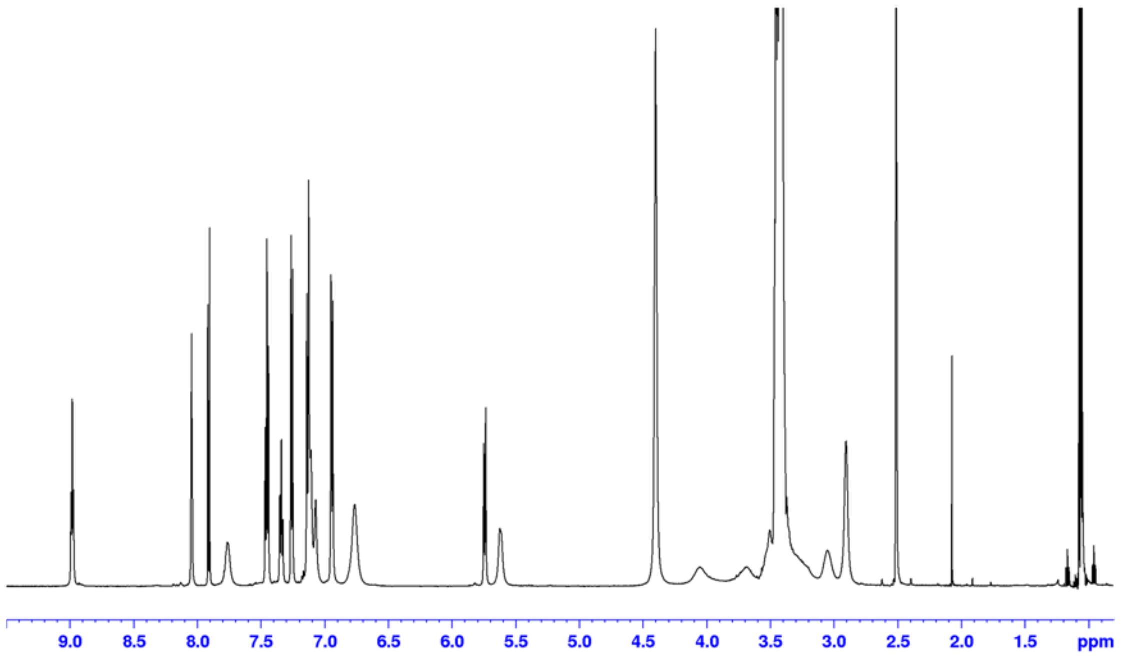

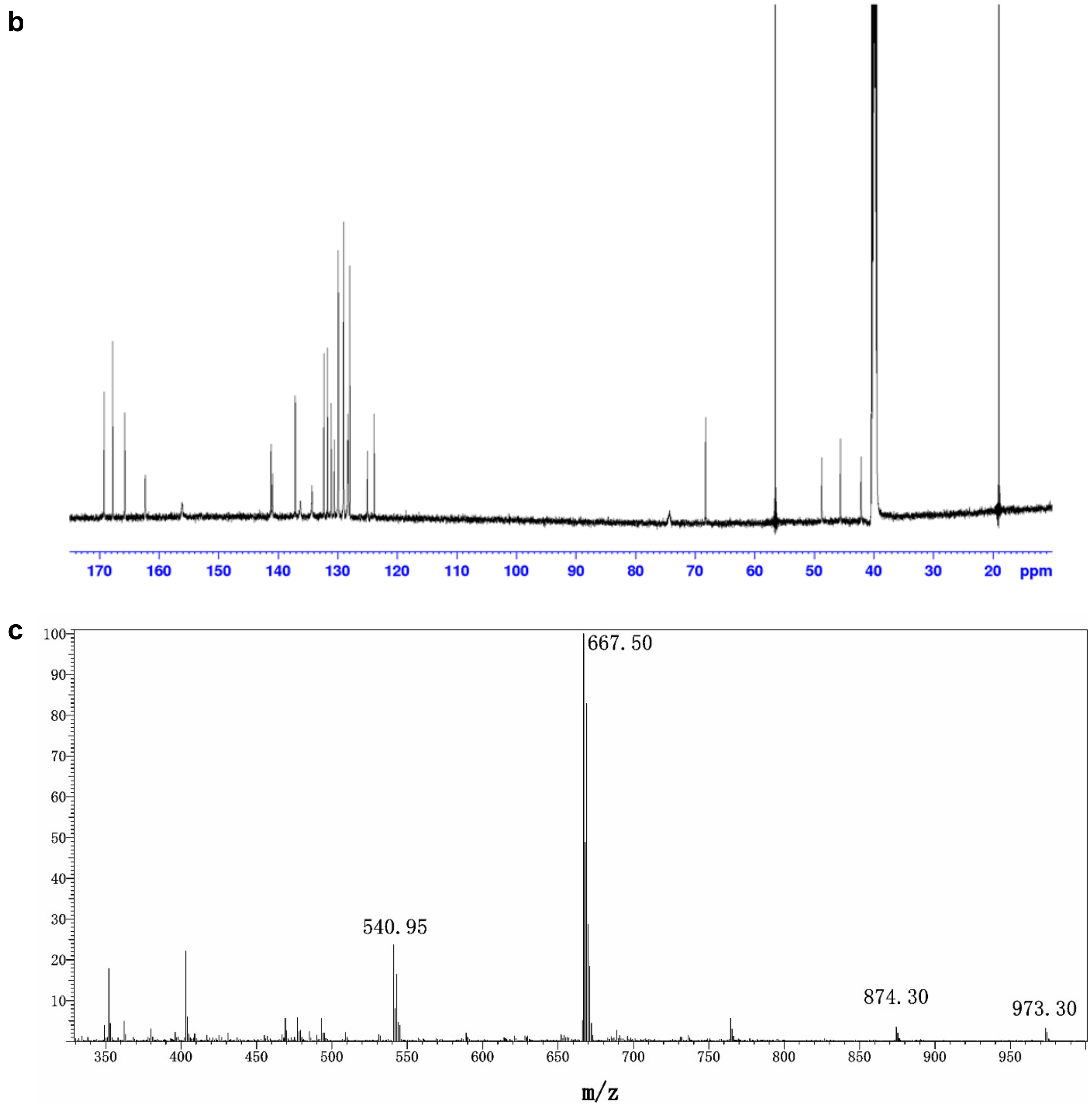

Supplementary Fig. 17. NMR and MS characterization of H203. a) & b). ^{1}H and ^{13}C NMR spectra of H203. **c**) Mass spectrum of H203.

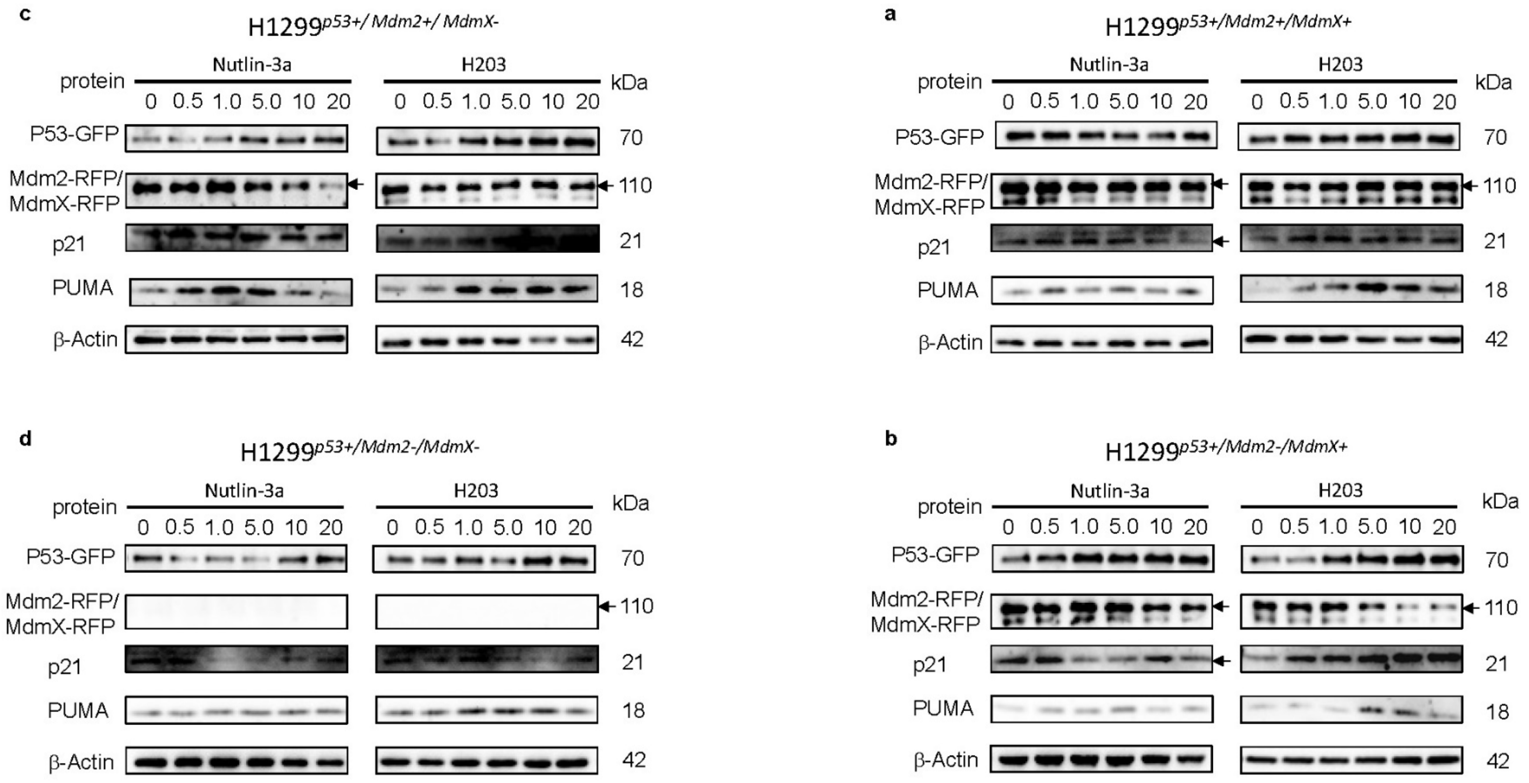

Supplementary Fig. 18 Western blotting assay of the inhibitory effects of H203, in comparison with nutlin-3a, on preventing the p53 degradation mediated by Mdm2 and MdmX in H1299^{p53+} cells. The data are representative immunoblot results from three independent experiments. Arrows indicate protein bands. The cells were exposed to nutlin-3a and H203 for 48 h. **a**) H1299$^{p53+/Mdm2+/MdmX+}$ cells. **b**) H1299$^{p53+/Mdm2-/MdmX+}$ cells. **c**) H1299$^{p53+/Mdm2+/MdmX-}$ cells. **d**) H1299$^{p53+/Mdm2-/MdmX-}$ cells. These samples derive from the same experiments and those blots were processed in parallel. Source data are provided as a Source Data file.

a)

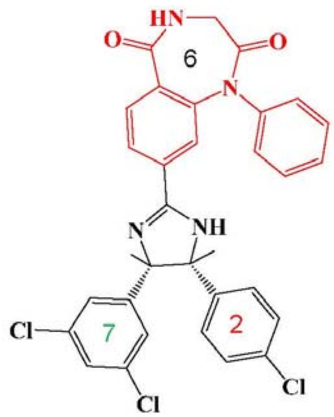

H210

b)

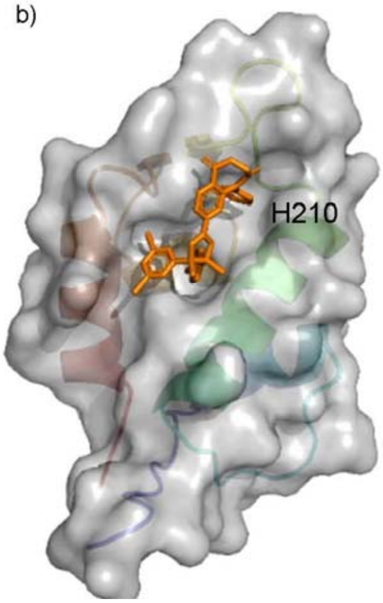

c)

**Name**	**Molecular Formula**	**Weight (g/mol)**	**MdmX**		**Mdm2**	
			**Score (kcal/mol)**	$\boldsymbol{K_i}$ **(nM)**	**Score (kcal/mol)**	***Ki*** **(nM)**
nutlin-3a	$C_{38}H_{58}O_2N_2$	576.91	-6.49	16765	-10.38	23.12
H202	$C_{29}H_{24}O_4N_6Cl_2$	591.45	-9.86	59.25	-11.19	6.31
H203	$C_{35}H_{28}O_4N_6Cl_2$	667.55	-11.04	8.13	-12.17	1.19
H210	$C_{32}H_{25}O_2N_4Cl_3$	603.93	-11.65	2.89	-6.81	9.66

Supplementary Fig. 19. Comparison of H210 with nutlin-3a, H202 and H203 with autodocking. a) Molecular structure of H210; **b**) Structural model of N-MdmX in complex with H210 generated from autodocking. **c**)

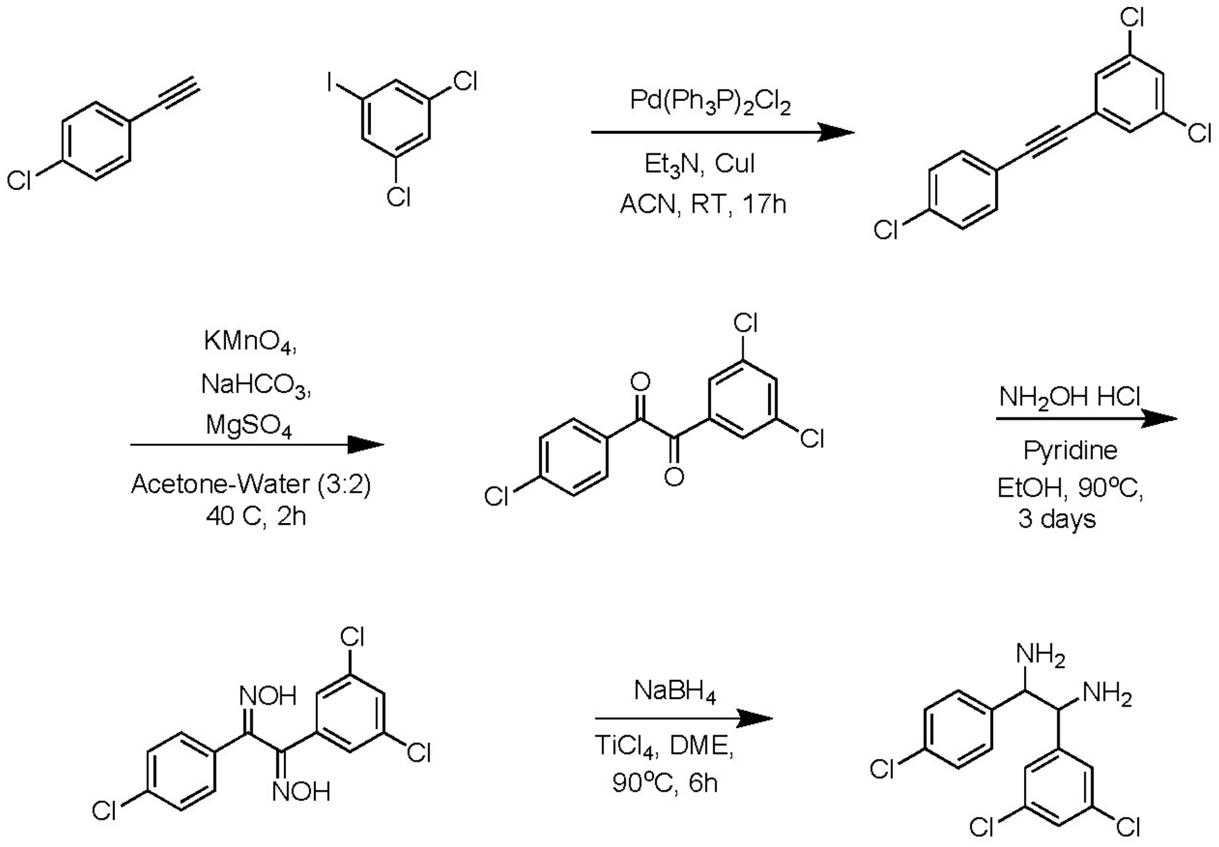

Supplementary Fig. 20. Synthesis of 1-(4-chlorophenyl)-2-(3,5-dichlorophenyl)ethane-1,2-diamine.
