# Supplementary material for: Leveraging the multivalent p53 peptide-MdmX interaction to guide the improvement of small molecule inhibitors
Source: Nat Commun. 2022 Feb 28;13:1087. doi: 10.1038/s41467-022-28721-x (PMC8885691; doi:10.1038/s41467-022-28721-x)
Supplement: Supplementary file 2 — Reporting Summary [file 41467_2022_28721_MOESM2_ESM.pdf]

nature research

Corresponding author(s): Zhengding Su

Last updated by author(s): January 26, 2022

Reporting Summary

Nature Research wishes to improve the reproducibility of the work that we publish. This form provides structure for consistency and transparency in reporting. For further information on Nature Research policies, see our Editorial Policies and the Editorial Policy Checklist.

Statistics

For all statistical analyses, confirm that the following items are present in the figure legend, table legend, main text, or Methods section.

n/a	Confirmed	
☐	☒	The exact sample size (*n*) for each experimental group/condition, given as a discrete number and unit of measurement
☐	☒	A statement on whether measurements were taken from distinct samples or whether the same sample was measured repeatedly
☒	☐	The statistical test(s) used AND whether they are one- or two-sided<br>*Only common tests should be described solely by name; describe more complex techniques in the Methods section.*
☒	☐	A description of all covariates tested
☒	☐	A description of any assumptions or corrections, such as tests of normality and adjustment for multiple comparisons
☐	☒	A full description of the statistical parameters including central tendency (e.g. means) or other basic estimates (e.g. regression coefficient) AND variation (e.g. standard deviation) or associated estimates of uncertainty (e.g. confidence intervals)
☒	☐	For null hypothesis testing, the test statistic (e.g. *F*, *t*, *r*) with confidence intervals, effect sizes, degrees of freedom and *P* value noted<br>*Give P values as exact values whenever suitable.*
☒	☐	For Bayesian analysis, information on the choice of priors and Markov chain Monte Carlo settings
☒	☐	For hierarchical and complex designs, identification of the appropriate level for tests and full reporting of outcomes
☒	☐	Estimates of effect sizes (e.g. Cohen's *d*, Pearson's *r*), indicating how they were calculated

Our web collection on statistics for biologists contains articles on many of the points above.

Software and code

Policy information about availability of computer code

Data collection	HKL2000 for crystal diffraction collection; Topspin3.0 for NMR titration and NOE; Origin 7 ITC 200 for ITC titration; Gens CHS 3.03 for FP data collection.
Data analysis	AutoPROC, XDS, DIALS, Porpoise and Xia2 for crystal diffraction data integration, scaling, reduction and phasing; CCP4i2 for molecular replacement, refinement; NMRViewJ9.2.b20 for displaying HSQC spectra; NMRPipe for NOE data processing; GUARDD/MATLAB2018a for CPMG data processing; Origin 7.0 for ITC data analysis; AMBER99SB-ILDN, TIP3P and GROMACS for molecule dynamics simulation; Origin 2018 for FP data fitting

For manuscripts utilizing custom algorithms or software that are central to the research but not yet described in published literature, software must be made available to editors and reviewers. We strongly encourage code deposition in a community repository (e.g. GitHub). See the Nature Research guidelines for submitting code & software for further information.

Data

Policy information about availability of data

All manuscripts must include a data availability statement. This statement should provide the following information, where applicable:

- Accession codes, unique identifiers, or web links for publicly available datasets
- A list of figures that have associated raw data
- A description of any restrictions on data availability

The crystal structure data generated in this study have been deposited in the Protein Data Bank under accession code 7C3Y.pdb [http://doi.org/10.2210/pdb7C3Y/pdb] (N-MdmX/nutlin-3a), 7C44.pdb [http://doi.org/p10.2210/pdb7C44/pdb] (N-MdmX/nutlin-3a),7C3Q.pdb [http://doi.org/p10.2210/pdb7C3Q/pdb] (N-MdmX/nutlin-3a), 5ZXF.pdb [http://doi.org/10.2210/pdb5ZXF/pdb] (N-Mdm2/nutlin-3a), 5ZO2.pdb [http://doi.org/p210.2210/pdb5ZO2/pdb] (N-MdmX/nutlin-3a), and 7EL4 [http://doi.org/p10.2210/pdb7EL4/pdb] (N-MdmX/nutlin-3ap53p analog). The initial structure model for Mdm2 was adopted from an X-ray crystal structure of

N-Mdm2/nutlin-3a under accession code of 4J3E [http://doi.org/10.2210/pdb4J3E/pdb]. The initial structure models for MdmX were the X-ray crystal structures of N-MdmX/Cpd15 and N-MdmX/p53 analog under accession code of 6Q9W [http://doi.org/10.2210/pdb6Q9W/pdb] and 6V4F [http://doi.org/10.2210/pdb6V4F/pdb]. The X-ray crystal structures of the MdmX/p53 peptide complexes and the MdmX/WK298 complexes used for structure comparison were under accession code of 3DAB [http://doi.org/10.2210/pdb3DAB/pdb] and 3LBJ [http://doi.org/10.2210/pdb3LBJ/pdb], respectively. The initial structure of N-Mdm2 for MD was isolated from the N-Mdm2/p53p complexes under accession code of 1YCR [http://doi.org/10.2210/pdb1YCR/pdb]. Source data are provided with this paper.

Field-specific reporting

Please select the one below that is the best fit for your research. If you are not sure, read the appropriate sections before making your selection.

☒ Life sciences ☐ Behavioural & social sciences ☐ Ecological, evolutionary & environmental sciences

For a reference copy of the document with all sections, see nature.com/documents/nr-reporting-summary-flat.pdf

Life sciences study design

All studies must disclose on these points even when the disclosure is negative.

Sample size	No sample size calculations were performed, but several replication were performed where appropriate for concordance and to establish consistent results.
Data exclusions	No data were excluded from the analyses.
Replication	Multiple biologically independent samples were performed in order to verify reproducibility for each experiment. All experiments were repeated at least twice. Detailed information on replicates were described in the figure legends. All attempts at replication were successful.<br>1) Protein crystallization structure determination has been successfully reproduced twice with diffracting crystals.<br>2) The NMR experiments were replicated with two successful independent samples and we chose a better one to present.<br>3) The compound screening were replicated with two successful independent samples and a mean was presented.<br>4) For fluorescence polarization assays, ITC Titration, real-time qPCR assay and MTT experiments, independent experimental repeats were performed at least three times to insure reliability and reproducibility of the results.<br>5) For western blot assay, at least two replicates with independent cell samples were taken to verify the reproducibility of the experimental finding.
Randomization	Randomization was not considered within the experimental samples tested since cell lines used were genetically identical.
Blinding	Blinding was not considered within the experimental samples tested since this study was not practical and no subjective results were gathered.

Reporting for specific materials, systems and methods

We require information from authors about some types of materials, experimental systems and methods used in many studies. Here, indicate whether each material, system or method listed is relevant to your study. If you are not sure if a list item applies to your research, read the appropriate section before selecting a response.

Materials & experimental systems

n/a	Involved in the study	
☐	☒	Antibodies
☐	☒	Eukaryotic cell lines
☒	☐	Palaeontology and archaeology
☒	☐	Animals and other organisms
☒	☐	Human research participants
☒	☐	Clinical data
☒	☐	Dual use research of concern

Methods

n/a	Involved in the study	
☒	☐	ChIP-seq
☒	☐	Flow cytometry
☒	☐	MRI-based neuroimaging

Antibodies

Antibodies used	Primary antibody:<br>Rabbit anti TP53 Polyclonal Antibody IgG (CUSABIO, TX, USA; Cat#: CSB-PA15509AORB, Lot#: F0912A), 1:4000;<br>Mouse Anti-RFP tag Monoclonal Antibody (Solarbio, Beijing, China; Cat#: K20016M), 1:10000;<br>Rabbit anti P21 Polyclonal Antibody (Elabscience, Wuhan, China; Cat#: E-AB-40097), 1:500;<br>Rabbit anti human PUMA Monoclonal Antibody (Beyotime, Shanghai, China; Cat#: AF1204; Gene ID 27113), 1:1000;<br>Mouse Anti-beta Actin Monoclonal Antibody (Proteintech, Wuhan, China; Cat#: HRP-60008; Gene ID 60), 100ug/ml, 1:10000.<br>Secondary antibody:<br>HRP-conjugated goat anti rabbit IgG(H+L) (Proteintech, Wuhan, China; Cat#: SA00001-2), 1:10000;<br>HRP-conjugated goat anti mouse IgG(H+L) (Biosharp, Guangzhou, China; Cat#: BL001A), 0.8mg/ml, 1:10000.
Validation	The antibodies have been validated in other studies, the link of which could be found in the manufacturer's website. The manuals of all the antibodies coming with the reagents are included in Source Data file.<br>1) Rabbit anti TP53 Polyclonal Antibody IgG: Positive WB detected in Mouse kidney tissue 2.5μg/ml (http://www.cusabio.cn/Polyclonal-Antibodies/Tp53-Antibody-156219.html);<br>2) Mouse Anti-RFP tag Monoclonal Antibody: Western blot analysis with RFP tag antibody diluted at 1:10000 (https://www.solarbio.com/goods-64425.html);<br>3) Rabbit anti P21 Polyclonal Antibody: western blot analysis of MCF-7 and 293T using polyclonal antibody at dulution of 1:600 (https://www.elabscience.cn/search-keywords=e-ab-40097.html);<br>4) Rabbit anti human PUMA Monoclonal Antibody: category for apoptosis and metabolism. PUMA is involved in p53-dependent and -independent apoptosis induced by a variety of signals (https://www.beyotime.com/product/AF1204.htm);<br>5) Mouse Anti-beta Actin Monoclonal Antibody: western blot analysis of beta-actin in various tissues and cell lines using Proteintech antibody HRP-60008 at a dilution of 1:5000. 44 publications and cited applications on WB (https://www.ptglab.com/products/ACTB-Antibody-HRP-60008.htm).<br>6) HRP-conjugated goat anti rabbit IgG(H+L) : 1:2000-1:10000 for western blotting with ECL substrates (https://www.ptglab.com/products/HRP-conjugated-Affinipure-Goat-Anti-Rabbit-IgG-H-L-secondary-antibody.htm);<br>7) HRP-conjugated goat anti mouse IgG(H+L) : western blots: 1:4000-1:80000 (http://www.biosharp.cn/index/product/details/language/cn/product_id/78.html).

Eukaryotic cell lines

Policy information about cell lines

Cell line source(s)	H1299 (ATCC: CRL-5803), HCT116 (ATCC: CCL-247), RKO(ATCC: CRL-2577), H1648 (ATCC: CRL-5882) .
Authentication	Not authentication after purchase from ATCC.
Mycoplasma contamination	The cells were checked for mycoplasma contamination by Hoechst straining, and tested negative.
Commonly misidentified lines<br>(See ICLAC register)	No commonly misidentified cell lines were used.
