# Supplementary material for: Leveraging the multivalent p53 peptide-MdmX interaction to guide the improvement of small molecule inhibitors
Source: Nat Commun. 2022 Feb 28;13:1087. doi: 10.1038/s41467-022-28721-x (PMC8885691; doi:10.1038/s41467-022-28721-x)
Supplement: Supplementary file 3 — Source Data [file 41467_2022_28721_MOESM3_ESM.zip › Source data/Antibody verification/1-TP53 antibody Rabbit CSB-PA15509A0Rb.pdf]

Tp53 Antibody

**Product Code**	CSB-PA15509A0Rb
**Storage**	Upon receipt, store at -20°C or -80°C. Avoid repeated freeze.
**Uniprot No.**	P10361
**Immunogen**	Recombinant Rat Cellular tumor antigen p53 protein (1-391AA)
**Raised In**	Rabbit
**Species Reactivity**	Rat, Mouse
**Tested Applications**	ELISA, WB; Recommended dilution: WB:1:500-1:5000
**Form**	Liquid
**Conjugate**	Non-conjugated
**Storage Buffer**	Preservative: 0.03% Proclin 300<br>Constituents: 50% Glycerol, 0.01M PBS, PH 7.4
**Purification Method**	>95%, Protein G purified
**Isotype**	IgG
**Clonality**	Polyclonal
**Product Type**	Polyclonal Antibody
**Immunogen Species**	Rattus norvegicus (Rat)
**Research Area**	Cell Biology
**Gene Names**	Tp53

Image

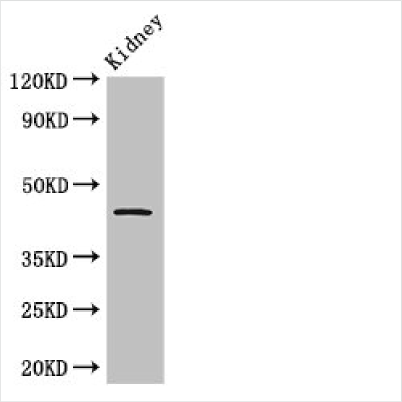

Western Blot
Positive WB detected in: Mouse kidney tissue
All lanes: Tp53 antibody at 2.5µg/ml
Secondary
Goat polyclonal to rabbit IgG at 1/50000 dilution
Predicted band size: 44 kDa
Observed band size: 44 kDa
