# Supplementary material for: Leveraging the multivalent p53 peptide-MdmX interaction to guide the improvement of small molecule inhibitors
Source: Nat Commun. 2022 Feb 28;13:1087. doi: 10.1038/s41467-022-28721-x (PMC8885691; doi:10.1038/s41467-022-28721-x)
Supplement: Supplementary file 3 — Source Data [file 41467_2022_28721_MOESM3_ESM.zip › Source data/Antibody verification/2-Anti-RFP tag Solarbio.pdf]

Solarbio®
LIFE SCIENCES

Anti-RFP tag Monoclonal Antibody

Cat: K200016M

Summary:

【Product name】: Anti-VSV-G-tag antibody

【Source】: Mouse

【Isotype】: IgG1

【Species reactivity】: Human Mouse Rat

【Swiss Prot】:

【Gene ID】:

【Calculated】: MW:kDa

【Observed】: MW:32kDa

【Purification】: Affinity purification

【Tested applications】: WB

【Recommended dilution】: WB 1:10000-15000.

【WB Positive sample】: Recombinant protein

【Subcellular location】:

【Immunogen】: recombinant protein

【Storage】: Shipped at 4°C. Upon delivery aliquot and store at -20°C

Background:

The red fluorescent protein cloned from Discosoma coral (DsRed or drFP583) holds great promise for biotechnology and cell biology as a spectrally distinct companionor substitute for the green fluorescent protein(GFP) from the Aequorea jellyfish.

Solarbio®
LIFE SCIENCES

Verified picture

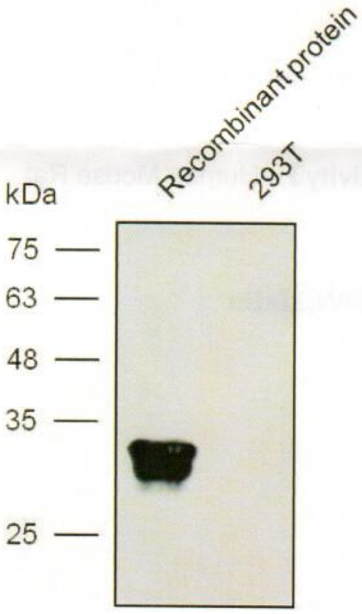

Western blot analysis with RFP tag antibody diluted at 1:10000

Sales:sales@solarbio.com
Tech:searvice@solarbio.com
