# Supplementary material for: Leveraging the multivalent p53 peptide-MdmX interaction to guide the improvement of small molecule inhibitors
Source: Nat Commun. 2022 Feb 28;13:1087. doi: 10.1038/s41467-022-28721-x (PMC8885691; doi:10.1038/s41467-022-28721-x)
Supplement: Supplementary file 3 — Source Data [file 41467_2022_28721_MOESM3_ESM.zip › Source data/Antibody verification/3-P21 Polyclonal Antibody 18kDa Rabbit IgG Elabscience.pdf]

Tel:86-27-87879180
Techsupport:86-27-87526315
www.elabscience.cn
E-mail:antibody@elabscience.cn
Elabscience Biotechnology Co.,Ltd

Elabscience®

P21 Polyclonal Antibody

**Catalog No.**	E-AB-40097	**Reactivity**	H
**Storage**	Store at -20°C. Avoid freeze / thaw cycles.	**Host**	Rabbit
**Applications**	WB	**Isotype**	IgG

Note: Centrifuge before opening to ensure complete recovery of vial contents.

Images

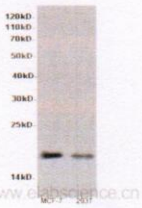

Western Blot analysis of MCF-7 and 293T cells using P21 Polyclonal Antibody at dilution of 1:600

Immunogen Information

**Immunogen**	Recombinant Human P21 protein
**GeneID**	1026
**Swissprot**	P38936
**Synonyms**	CAP20, CDK-interacting protein 1, CDKI, CDKN1, Cdkn1a, CDN1A_HUMAN, CIP1, MDA-6, MDA6, p21, P21 protein, p21CIP1, p21WAF, PIC1, SDI1, SLC12A9, WAF1

Product Information

**Calculated MW**	18kDa
**Observed MW**	18kDa
**Buffer**	PBS with 0.05% Proclin300, 50% glycerol, pH7.3.
**Purify**	Affinity purification
**Dilution**	WB 1:500-1:1000

Background

May be involved in p53/TP53 mediated inhibition of cellular proliferation in response to DNA damage. Binds to and inhibits cyclin-dependent kinase activity, preventing phosphorylation of critical cyclin-dependent kinase substrates and blocking cell cycle progression. Functions in the nuclear localization and assembly of cyclin D-CDK4 complex and promotes its kinase activity towards RB1. At higher stoichiometric ratios, inhibits the kinase activity of the cyclin D-CDK4 complex. Inhibits DNA synthesis by DNA polymerase delta by competing with POLD3 for PCNA binding (PubMed:11595739).

For Research Use Only
Thank you for your recent purchase.
If you would like to learn more about antibodies,please visit www.elabscience.cn.

Focus on your research
Service for life science

Applications:WB-Western Blot IHC-Immunohistochemistry IF-Immunofluorescence IP-Immunoprecipitation FC-Flow cytometry ChIP-Chromatin Immunoprecipitation Reactivity: H-Human R-Rat M-Mouse Mk-Monkey Dg-Dog Ch-Chicken Hm-Hamster Rb-Rabbit Sh-Sheep Pg-Pig Z-Zebrafish X-Xenopus C-Cow.
