# Supplementary material for: Leveraging the multivalent p53 peptide-MdmX interaction to guide the improvement of small molecule inhibitors
Source: Nat Commun. 2022 Feb 28;13:1087. doi: 10.1038/s41467-022-28721-x (PMC8885691; doi:10.1038/s41467-022-28721-x)
Supplement: Supplementary file 3 — Source Data [file 41467_2022_28721_MOESM3_ESM.zip › Source data/Antibody verification/4-Anti-PUMA antibody.pdf]

碧云天 Beyotime
碧云天网站 微信公众号
碧云天生物技术 / Beyotime Biotechnology
订货热线：400-1683301 或 800-8283301
订货e-mail：order@beyotime.com
技术咨询：info@beyotime.com
网址：http://www.beyotime.com

# PUMA Rabbit Monoclonal Antibody

| 产品编号 | 产品名称 | 包装 |
|---|---|---|
| AF1204 | PUMA Rabbit Monoclonal Antibody | 50μl |

## 产品简介：

| 来源 | 用途 | 交叉反应性 | 分子量 |
|---|---|---|---|
| Rabbit | WB, IF, IHC, ICC, FC | H, M, R | 18 kDa |

WB, Western blot; IP, Immunoprecipitation; IF, Immunofluorescence; IHC, Immunohistochemistry; ICC, Immunocytochemistry; FC, Flow Cytometry; ELISA, Enzyme-linked Immunosorbent Assay; ChIP, Chromatin Immunoprecipitation Assay.

H, Human; M, Mouse; R, Rat; C, Chicken; Cw, Cow; Dg, Dog; Gp, Guinea pig; Hm, Hamster; Hr, Horse; Mk, Monkey; Pg, Pig; Rb, Rabbit; S, Sheep; Z, Zebrafish; All, all species expected.

- 配套提供了 Western 一抗稀释液，可以用于 Western 检测或其它适当用途时的一抗稀释。
- 建议抗体使用时的稀释比例如下(实际使用时需根据抗原水平的高低作适当调整)：

| WB | IP | IF | IHC | ICC | FC | ELISA | ChIP |
|---|---|---|---|---|---|---|---|
| 1:1,000-1:2,000 | - | 1:50-1:200 | 1:50-1:200 | 1:50-1:200 | 1:50-1:100 | - | - |

- 抗体详细信息如下：

| About this Antibody | |
|---|---|
| Name | PUMA Rabbit Monoclonal Antibody |
| Category | Rabbit Monoclonal Antibody (RabMAb); Primary antibody |
| Isotype | IgG |
| Purification | Affinity purification |
| **About the Immunogen** | |
| Immunogen | Recombinant protein |
| Gene ID | 27113(Human); 170770(Mouse); 317673(Rat) |
| SwissProt | Q9BXH1(Human); Q99ML1(Mouse); Q80ZG6(Rat) |
| Synonyms | JFY-1; JFY1; PUMA |
| Category | Apoptosis; Metabolism |
| Background | The expression of PUMA is regulated by the tumor suppressor p53. PUMA is involved in p53-dependent and -independent apoptosis induced by a variety of signals, and is regulated by transcription factors, not by post-translational modifications. After activation, PUMA interacts with antiapoptotic Bcl-2 family members, thus freeing Bax and/or Bak which are then able to signal apoptosis to the mitochondria. Following mitochondrial dysfunction, the caspase cascade is activated ultimately leading to cell death. Several studies have shown that PUMA function is affected or absent in cancer cells. Additionally, many human tumors contain p53 mutations, which results in no induction of PUMA, even after DNA damage induced through irradiation or chemotherapy drugs.Other cancers, which exhibit overexpression of antiapotptic Bcl-2 family proteins, counteract and overpower PUMA-induced apoptosis. |

## 包装清单：

| 产品编号 | 产品名称 | 包装 |
|---|---|---|
| AF1204 | PUMA Rabbit Monoclonal Antibody | 50μl |
| AZ050 | Western一抗稀释液 | 50ml |
| — | 说明书 | 1份 |

## 保存条件：

PUMA Rabbit Monoclonal Antibody -20ºC保存，Western一抗稀释液-20ºC或4ºC保存，一年有效。Western一抗稀释液优先推荐4ºC保存，长期不使用可以考虑-20ºC保存，但冻融可能会导致出现轻微的浑浊和少量不溶物。

## 注意事项:

- 如果本抗体用于Western blot (WB)、免疫荧光(IF)、免疫细胞化学(ICC)等实验，请注意回收使用过的稀释抗体。回收的抗体通常至少可以重复使用5-10次。稀释后的抗体，包括已经使用过的稀释抗体，请4ºC保存。
- 回收后重复使用的抗体，使用方法同新鲜稀释的抗体。如果在重复使用过程中发现抗体出现轻微混浊现象，可以10,000g离心1-3分钟，取上清用于后续检测。如果回收的抗体出现明显的絮状物或长霉长菌等情况，则可以考虑废弃该抗体。
- 提供的Western一抗稀释液也可以用于免疫荧光(IF)、免疫组化(IHC)、免疫细胞化学(ICC)等适当用途。如果希望获得最佳的检测效果，请考虑使用上述检测专用的一抗稀释液。
- 本产品仅限于专业人员的科学研究用，不得用于临床诊断或治疗，不得用于食品或药品，不得存放于普通住宅内。
- 为了您的安全和健康，请穿实验服并戴一次性手套操作。

## 使用说明:

**请根据抗体的实际用途选择相应的使用方法。**

1. **Western检测：**
   a. 按照推荐的稀释比例用碧云天提供的Western一抗稀释液稀释抗体。
   b. 把经过封闭的蛋白膜与稀释好的一抗4ºC缓慢摇动过夜或室温缓慢摇动2小时，确保稀释的抗体至少能在摇动的瞬间覆盖蛋白膜。
   c. 回收稀释的一抗，4ºC保存以备下次继续使用。
   d. 按照Western的实验步骤进行后续的洗涤、二抗孵育、洗涤和检测等操作。具体操作可以参考如下网页：
   http://www.beyotime.com/support/western.htm
2. **免疫染色：**
   可以使用碧云天生产的免疫染色一抗稀释液(P0103)稀释抗体，使用后注意回收稀释好的一抗，具体操作可以参考如下网页：
   http://www.beyotime.com/support/immunol-staining.htm
3. **其它实验操作请自行参考适当的protocol进行。**
4. **代表性图片：**

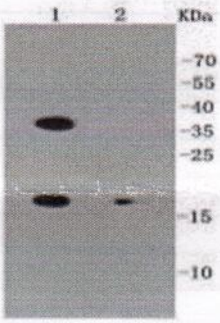


Fig. 1. Western blot analysis of PUMA on different lysates using anti-PUMA antibody at 1/1,000 dilution. Positive control: Lane 1: Hela; Lane 2: K562

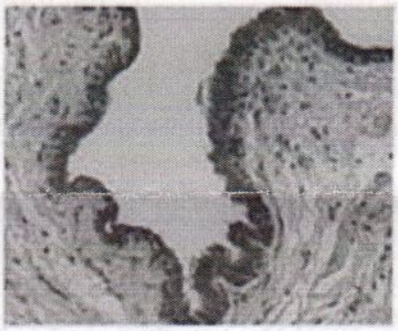

Fig. 2. Immunohistochemical analysis of paraffin-embedded human breast carcinoma tissue using anti-PUMA antibody. Counter stained with hematoxylin.

## 相关产品:

| 产品编号 | 产品名称 | 包装 |
|---|---|---|
| P0006 | Bradford蛋白浓度测定试剂盒 | 1000次 |
| P0010 | BCA蛋白浓度测定试剂盒(增强型) | 500次 |
| P0012 | BCA蛋白浓度测定试剂盒 | 500次 |
| P0012A | SDS-PAGE凝胶配制试剂盒 | 1盒 |
| P0012AC | SDS-PAGE凝胶快速配制试剂盒 | 1盒 |
| P0013 | Western及IP细胞裂解液 | 100ml |
| P0013B | RIPA裂解液(强) | 100ml |
| P0014B | SDS-PAGE电泳液 | 10×1L |
| P0015 | SDS-PAGE蛋白上样缓冲液(5X) | 2ml |
| P0018 | BeyoECL Plus(超敏ECL化学发光试剂盒) | 共100ml |
| P0018A | BeyoECL Star(特超敏ECL化学发光试剂盒) | 共100ml |
| P0020 | 显影定影试剂盒 | 各1升 |
| P0021B | Western转膜液 | 10×1L |
