# Supplementary material for: Leveraging the multivalent p53 peptide-MdmX interaction to guide the improvement of small molecule inhibitors
Source: Nat Commun. 2022 Feb 28;13:1087. doi: 10.1038/s41467-022-28721-x (PMC8885691; doi:10.1038/s41467-022-28721-x)
Supplement: Supplementary file 3 — Source Data [file 41467_2022_28721_MOESM3_ESM.zip › Source data/Antibody verification/5-beta Actin Monoclonal antibody HRP-60008.pdf]

For Research Use Only

beta Actin Monoclonal ANTIBODY

Catalog Number:HRP-60008 44 Publications

proteintech® Antibodies | ELISA kits | Proteins
www.ptgcn.com

Basic Information

Catalog Number:
HRP-60008

Size:
1000 μg/ml

Source:
Mouse

Isotype:
IgM

Purification Method:
Caprylic acid/ammonium sulfate precipitation

Immunogen Catalog Number:

GenBank Accession Number:
BC002409

GeneID (NCBI):
60

Full Name:
actin, beta

Calculated MW:
375aa,42 kDa

Observed MW:
42 kDa

Recommended Dilutions:
WB 1:2000-1:16000

Applications

Tested Applications:
WB, ELISA

Cited Applications:
WB

Species Specificity:
human, mouse, rat, zebrafish,plant

Cited Species:
Chinese hamster, human, mouse, rat

Positive Controls:

WB : HeLa cells;

Background Information

Beta actin, also named as ACTB and F-Actin, belongs to the actin family. Actins are highly conserved globular proteins that are involved in various types of cell motility and are ubiquitously expressed in all eukaryotic cells. At least six isoforms of actins are known in mammals and other vertebrates: alpha (ACTC1, cardiac muscle 1), alpha 1 (ACTA1, skeletal muscle) and 2 (ACTA2, aortic smooth muscle), beta (ACTB), gamma 1 (ACTG1) and 2 (ACTG2, enteric smooth muscle). Beta and gamma 1 are two non-muscle actin proteins. Most actins consist of 376aa, while ACTG2 (rich in muscles) has 375aa and ACTG1(found in non-muscle cells) has only 374aa. Beta actin has been widely used as the internal control in RT-PCR and Western Blotting as a 42-kDa protein. This antibody can recognize all of actins. This antibody is conjugated with HRP.

Note: Do not add Azium (Sodium Azide or Smite) into the dilution buffer. Azium is the HRP inhibitor which decreases the enzymatic activity of HRP.

Notable Publications

Author	Pubmed ID	Journal	Application
Zhimin Huang	30374165	Nat Chem Biol	WB
Jiajie Li	30367515	J Oral Pathol Med	WB
Zhipeng Su	30361332	Mol Cancer Ther	WB

Storage

Storage:
Store at -20°C. Stable for one year after shipment.
Storage Buffer:
PBS with 0.02% Thimerosal, 50% Glycerol, pH 7.3.
Aliquoting is unnecessary for -20°C storage

Selected Validation Data

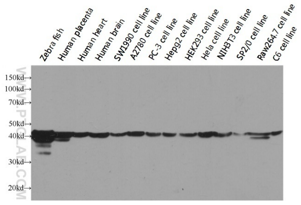

Western blot analysis of beta actin in various tissues and cell lines using Proteintech antibody HRP-60008 at a dilution of 1:5000.
