# Supplementary material for: Leveraging the multivalent p53 peptide-MdmX interaction to guide the improvement of small molecule inhibitors
Source: Nat Commun. 2022 Feb 28;13:1087. doi: 10.1038/s41467-022-28721-x (PMC8885691; doi:10.1038/s41467-022-28721-x)
Supplement: Supplementary file 3 — Source Data [file 41467_2022_28721_MOESM3_ESM.zip › Source data/Antibody verification/6-HRP-Goat Anti-Rabbit IgG Proteintech catSA00001-2 SA00001-2.pdf]

For Research Use Only.
Not For Use In Diagnostics.

# HRP-conjugated Affinipure Goat Anti-Rabbit IgG(H+L)

Catalog Number:SA00001-2

proteintech®
Antibodies | ELISA kits | Proteins

www.ptgcn.com

## Information

Catalog Number:
SA00001-2
Size:
100ul/500ul
Applications:
ELISA,WB

Product name:
HRP-conjugated Affinipure Goat Anti-Rabbit IgG(H+L)
Physical State:
Liquid
Conjugation:
Horseradish Peroxidase

## Recommended Dilutions

1:2000-1:10,000 for western blotting with ECL substrates
1:1000-1:20,000 for ELISA and Western blotting with chromogenic substrates

## Safety Notes

This product is for research use only,not for diagnostic or therapeutic use.

## Storage

Storage:
Store at -20°C. Avoid repeated freeze / thaw cycles.
Storage Buffer:
0.01M Sodium phosphate, 0.25M NaCl, 50% glycerol, pH7.6
Stabilizer:
3mg/ml BSA
Aliquoting is unnecessary for -20°C storage

## Purity

The antibody was purified from antisera by immunoaffinity chromatography using antigens coupled to agarose beads.

For technical support and original validation data for this product please contact:
T: 4006900926 E: Proteintech-CN@ptglab.com W: ptgcn.com

**This product is exclusively available under Proteintech Group brand and is not available to purchase from any other manufacturer.**
