# Supplementary material for: Leveraging the multivalent p53 peptide-MdmX interaction to guide the improvement of small molecule inhibitors
Source: Nat Commun. 2022 Feb 28;13:1087. doi: 10.1038/s41467-022-28721-x (PMC8885691; doi:10.1038/s41467-022-28721-x)
Supplement: Supplementary file 3 — Source Data [file 41467_2022_28721_MOESM3_ESM.zip › Source data/Antibody verification/7-HRP-secondary antibody goat to mouse IgG Biosharp BL001A.pdf]

山羊抗小鼠IgG HRP说明书

Goat Anti-Mouse IgG, Peroxidase Conjugated,H+L

产品编号	产品名称	规格
BL001A	山羊抗小鼠IgG HRP	0.1ml

规格： 原装进口为2.0ml　　进口分装为0.1ml （另加0.1ml甘油）

浓度： 0.8mg/ml

稀释范围： 免疫组化：1:400-1:4000ELISA/Western Blots：1:4000-1:80000

缓冲液： 0.01M的磷酸钠、0.25M的NaCl、pH7.1

稳定剂： 15mg/ml不含IgG的蛋白酶的牛血清白蛋白

防腐剂： 0.01% 硫柳汞 警告：叠氮钠是辣根过氧化物酶的抑制剂，不能作为防腐剂

分装及储存：

1、在进口原装抗体分装时，加入灭菌的50%甘油，能延长试剂的保存期。但应注意的是，此时抗体的使用浓度应降低一倍。

2、使用时，应按推荐的浓度对抗体进行稀释，但应注意，需现用现配，因为稀释后抗体的有效活性会很快降低。

保存条件： -20℃保存一年

Note:For in vitro research use only, not for diagnostic or therapeutic use,
This product is not a medical device.
注意：在体外研究使用，不用于诊断或治疗用途，本产品不是医疗装置。

电话：0551-68105770　68105780　**邮箱：**order@biosharp.cn

www.biosharp.cn
