# Supplementary material for: Leveraging the multivalent p53 peptide-MdmX interaction to guide the improvement of small molecule inhibitors
Source: Nat Commun. 2022 Feb 28;13:1087. doi: 10.1038/s41467-022-28721-x (PMC8885691; doi:10.1038/s41467-022-28721-x)
Supplement: Supplementary file 3 — Source Data [file 41467_2022_28721_MOESM3_ESM.zip › Source data/List of Source Data.docx]

**List of source data**

| **No** | **File name for publication** | **Name in data sheet** | **Source_data_file** |
| --- | --- | --- | --- |
| 1 | Fig.3c | Fig.3c | Source_data_1.xlsx |
| 2 | Fig.3d | Fig.3d | Source_data_1.xlsx |
| 3 | Fig.3e | Fig.3e | Source_data_1.xlsx |
| 4 | Fig.3f | Fig.3f | Source_data_1.xlsx |
| 6 | Fig.5a | Fig.5a | Source_data_1.xlsx |
| 7 | Fig.5b | Fig.5b | Source_data_1.xlsx |
| 8 | Fig.6a | Fig.6a | Source_data_1.xlsx |
| 9 | Fig.8a | Fig.8a | Source_data_1.xlsx |
| 8 | Fig.9a | Fig.9a | Source_data_1.xlsx |
| 9 | Fig.9b | Fig.9b | Source_data_1.xlsx |
| 10 | Fig.9c | Fig.9c | Source_data_1.xlsx |
| 11 | Fig.9d | Fig.9d | Source_data_1.xlsx |
| 12 | Fig.9e | Fig.9e | Source_data_1.xlsx |
| 13 | Supplementary Table 4 | Supplementary Table 4 | Source_data_1.xlsx |
| 14 | Supplementary Fig7c-top panel | Supplementary Fig7c-top panel | Source_data_1.xlsx |
| 15 | Supplementary Fig7c-top bottom | Supplementary Fig7c-top bottom | Source_data_1.xlsx |
| 16 | Supplementary Fig9b | Supplementary Fig9b | Source_data_1.xlsx |
| 17 | Supplementary Fig15a | Supplementary Fig15a | Source_data_1.xlsx |
| 18 | Supplementary Fig15b | Supplementary Fig15b | Source_data_1.xlsx |
| 19 | Supplementary Fig7c |  | Folder: Source data for Supplementary Fig7c |
| 20 | Supplementary Fig18 |  | Folder: Source data for Supplementary Fig18 |
| 21 | Antibody verification |  | Folder: Antibody verification |
