# Supplementary figures and images for: Leveraging the multivalent p53 peptide-MdmX interaction to guide the improvement of small molecule inhibitors
Source: Nat Commun. 2022 Feb 28;13:1087. doi: 10.1038/s41467-022-28721-x (PMC8885691; doi:10.1038/s41467-022-28721-x)

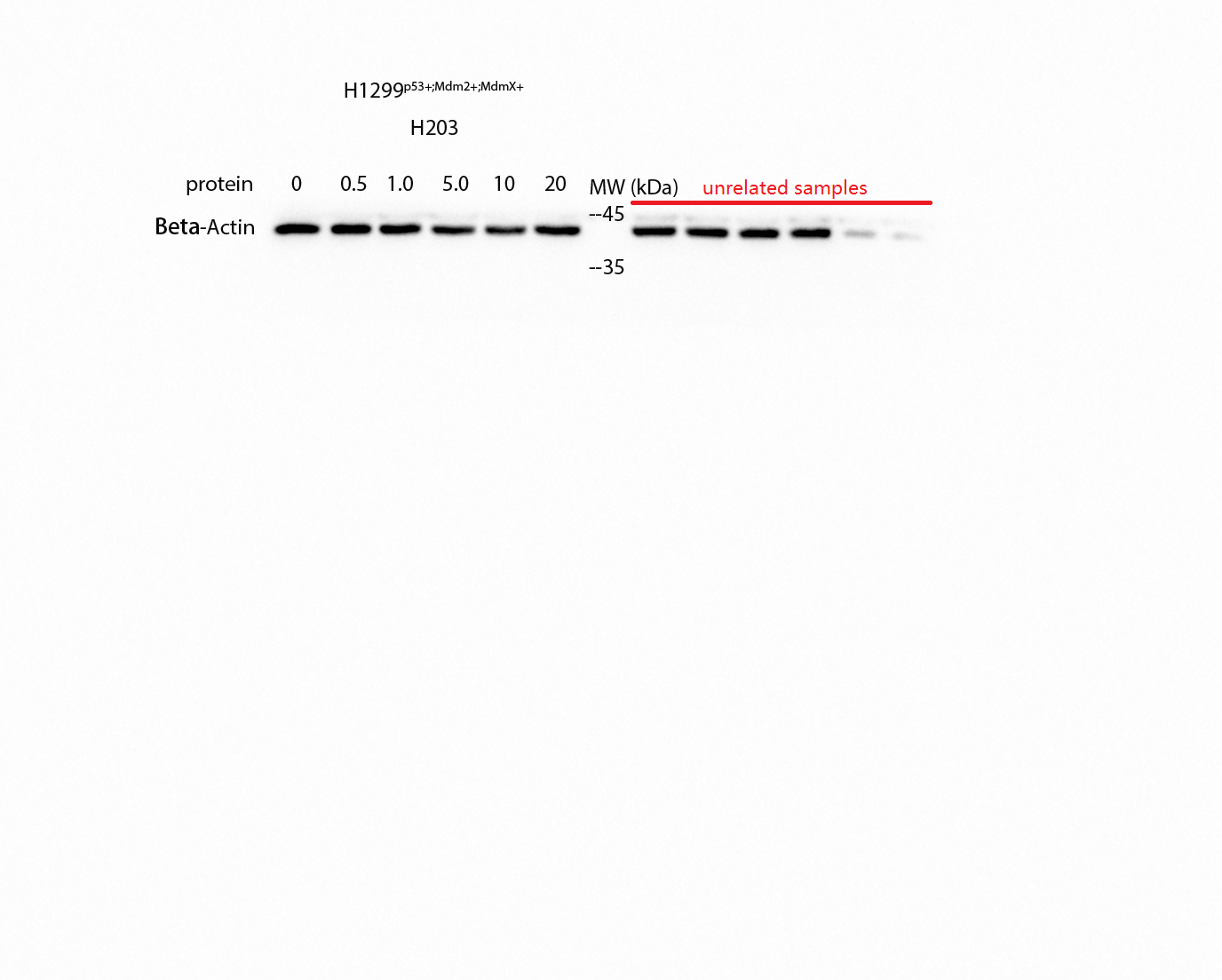

Supplement: Supplementary file 3 — Source Data [file 41467_2022_28721_MOESM3_ESM.zip › Source data/Source data for Supplementary Fig18/B-Actin/Supplementary Fig18a_beta-actin_H203.Tif]

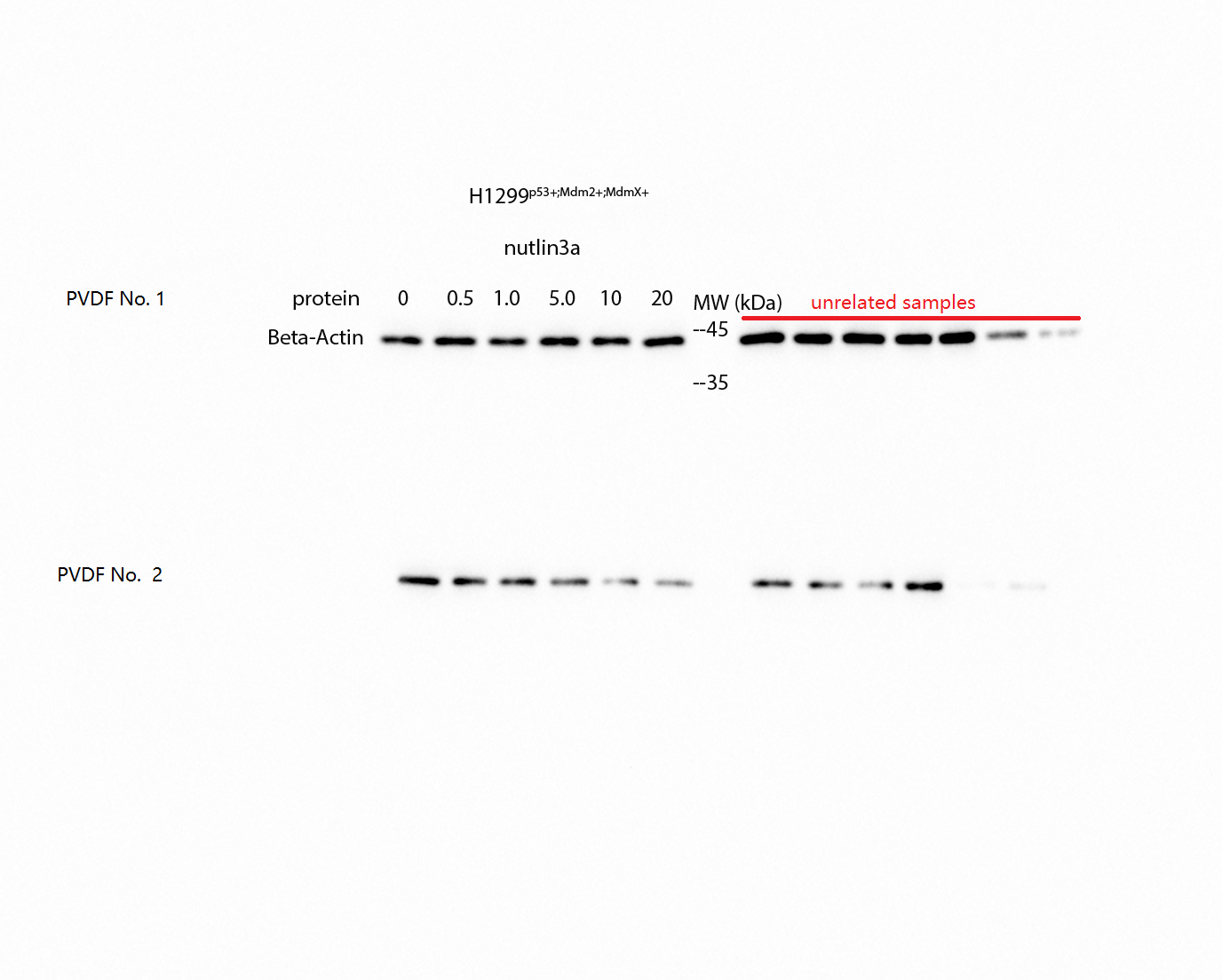

Supplement: Supplementary file 3 — Source Data [file 41467_2022_28721_MOESM3_ESM.zip › Source data/Source data for Supplementary Fig18/B-Actin/Supplementary Fig18a_beta-actin_nutlin3a.Tif]

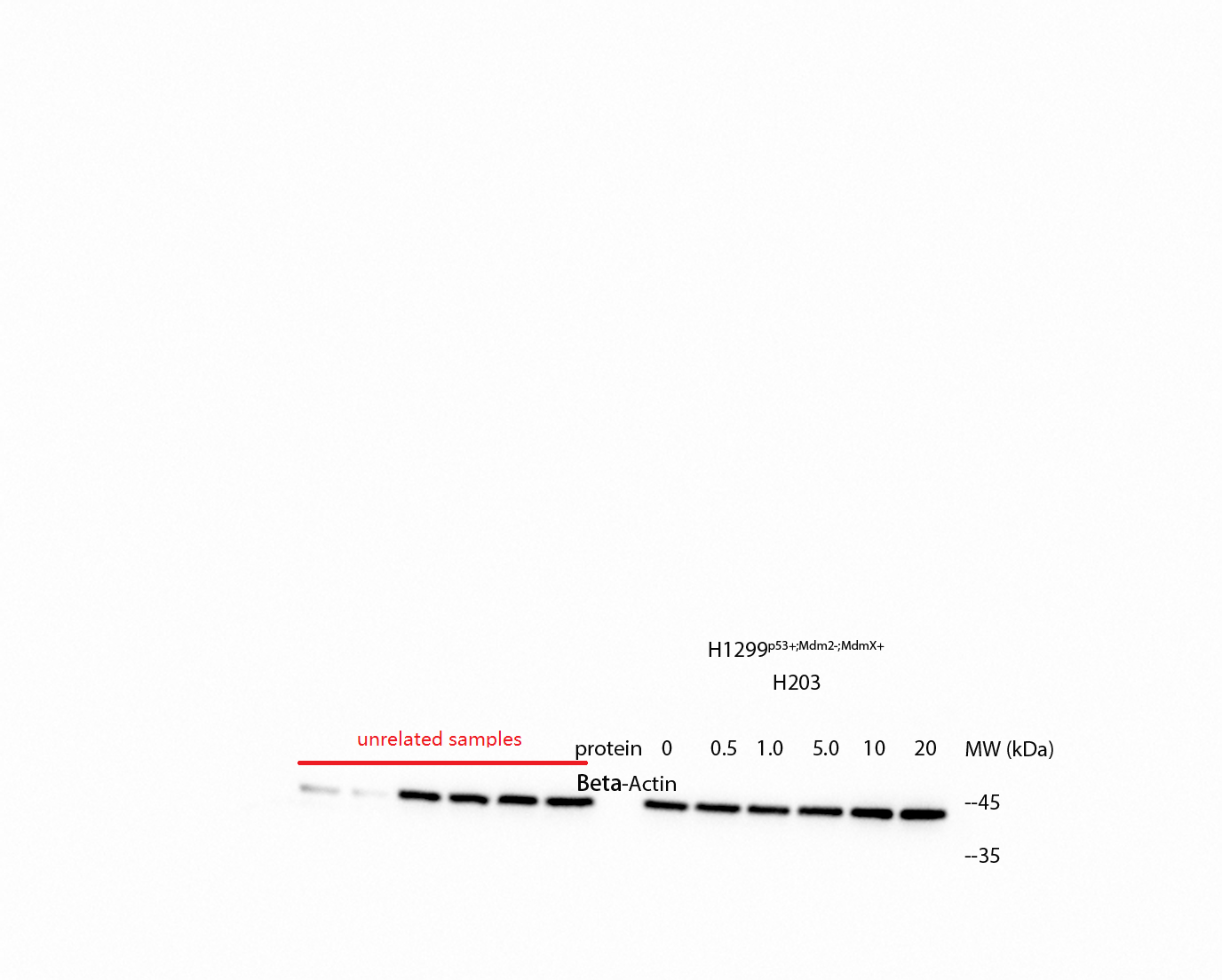

Supplement: Supplementary file 3 — Source Data [file 41467_2022_28721_MOESM3_ESM.zip › Source data/Source data for Supplementary Fig18/B-Actin/Supplementary Fig18b_beta-actin_H203.Tif]

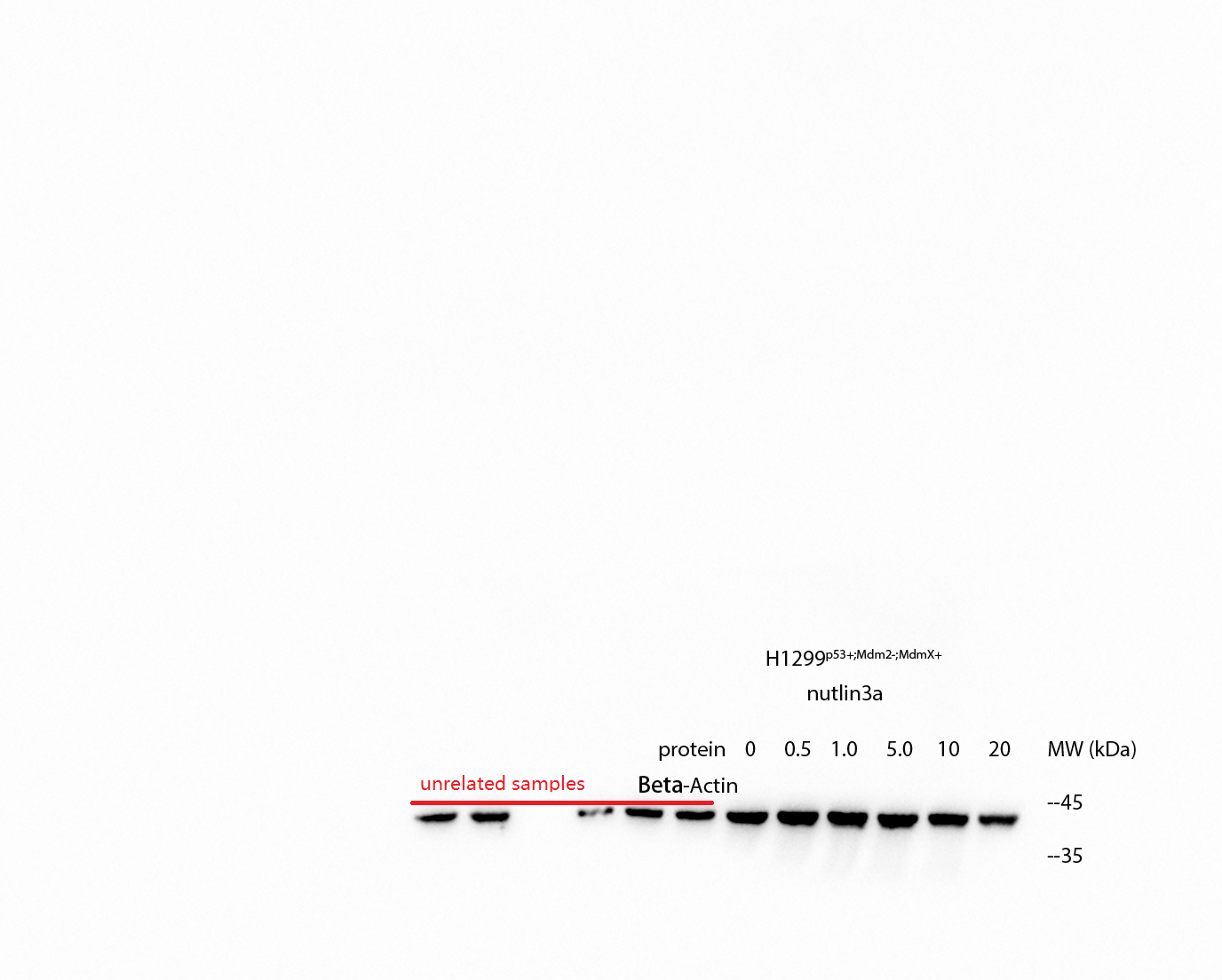

Supplement: Supplementary file 3 — Source Data [file 41467_2022_28721_MOESM3_ESM.zip › Source data/Source data for Supplementary Fig18/B-Actin/Supplementary Fig18b_beta-actin_nutlin3a.Tif]

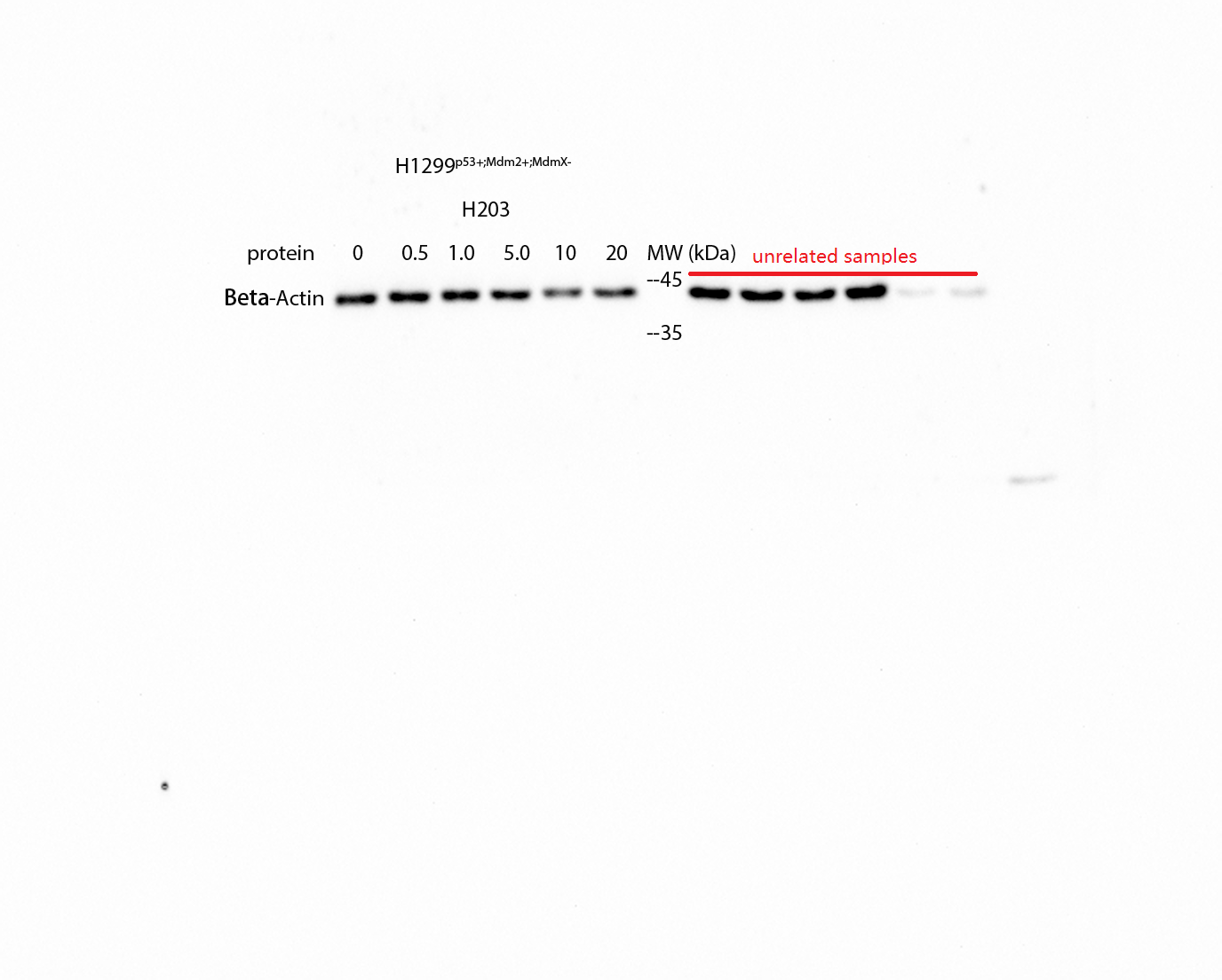

Supplement: Supplementary file 3 — Source Data [file 41467_2022_28721_MOESM3_ESM.zip › Source data/Source data for Supplementary Fig18/B-Actin/Supplementary Fig18c_beta-actin_H203.Tif]

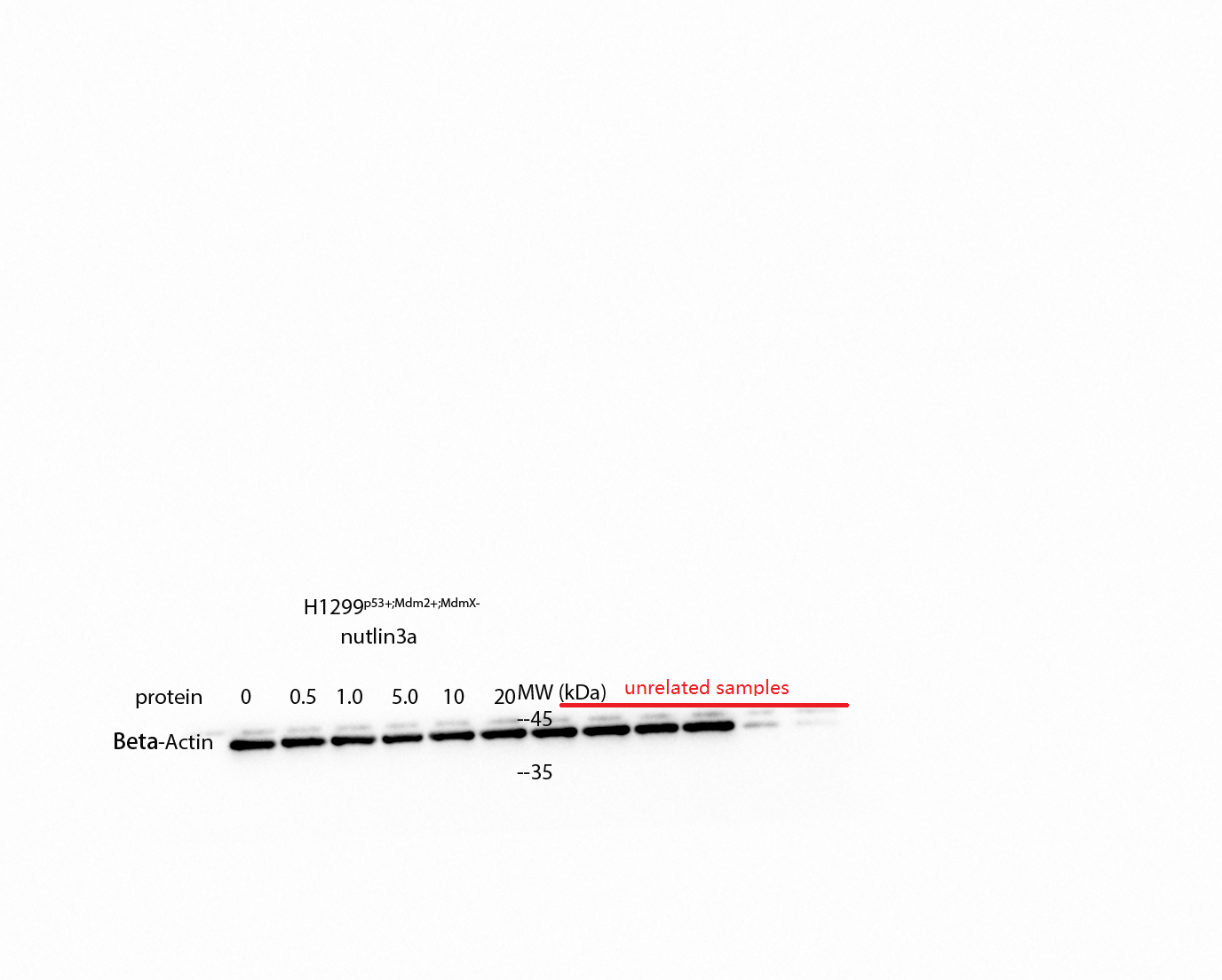

Supplement: Supplementary file 3 — Source Data [file 41467_2022_28721_MOESM3_ESM.zip › Source data/Source data for Supplementary Fig18/B-Actin/Supplementary Fig18c_beta-actin_nutlin3a.Tif]

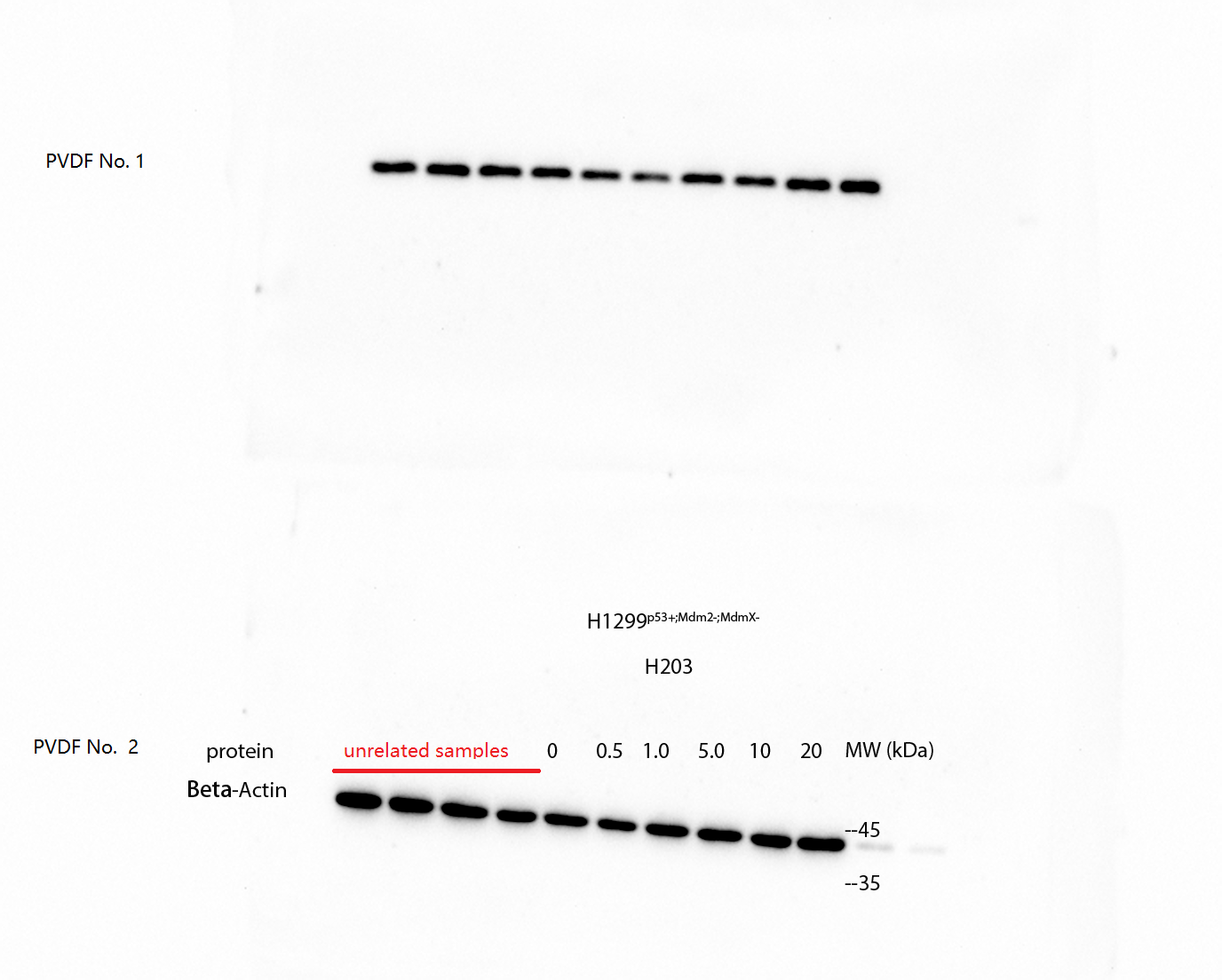

Supplement: Supplementary file 3 — Source Data [file 41467_2022_28721_MOESM3_ESM.zip › Source data/Source data for Supplementary Fig18/B-Actin/Supplementary Fig18d_beta-actin_H203.Tif]

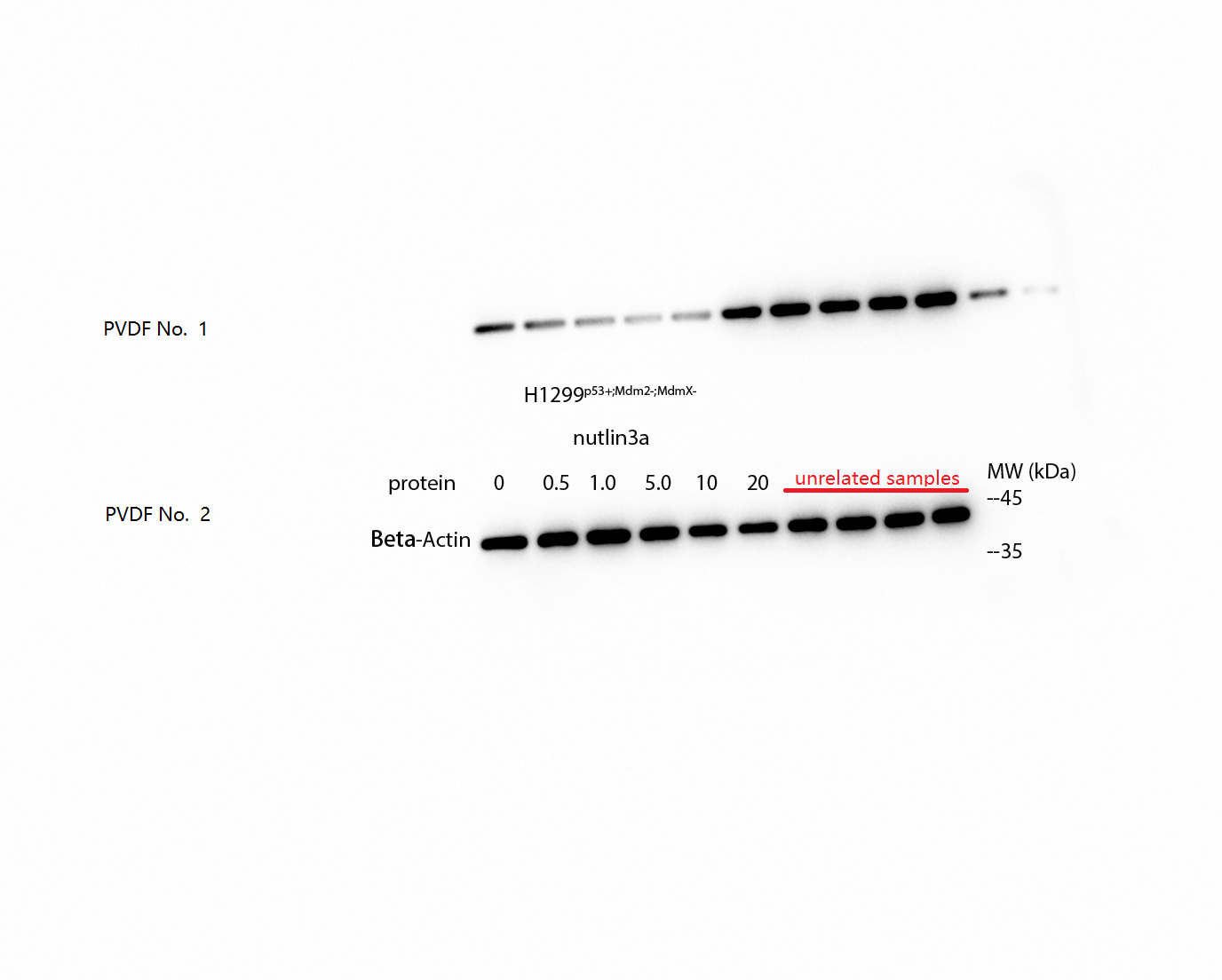

Supplement: Supplementary file 3 — Source Data [file 41467_2022_28721_MOESM3_ESM.zip › Source data/Source data for Supplementary Fig18/B-Actin/Supplementary Fig18d_beta-actin_nutlin3a.Tif]

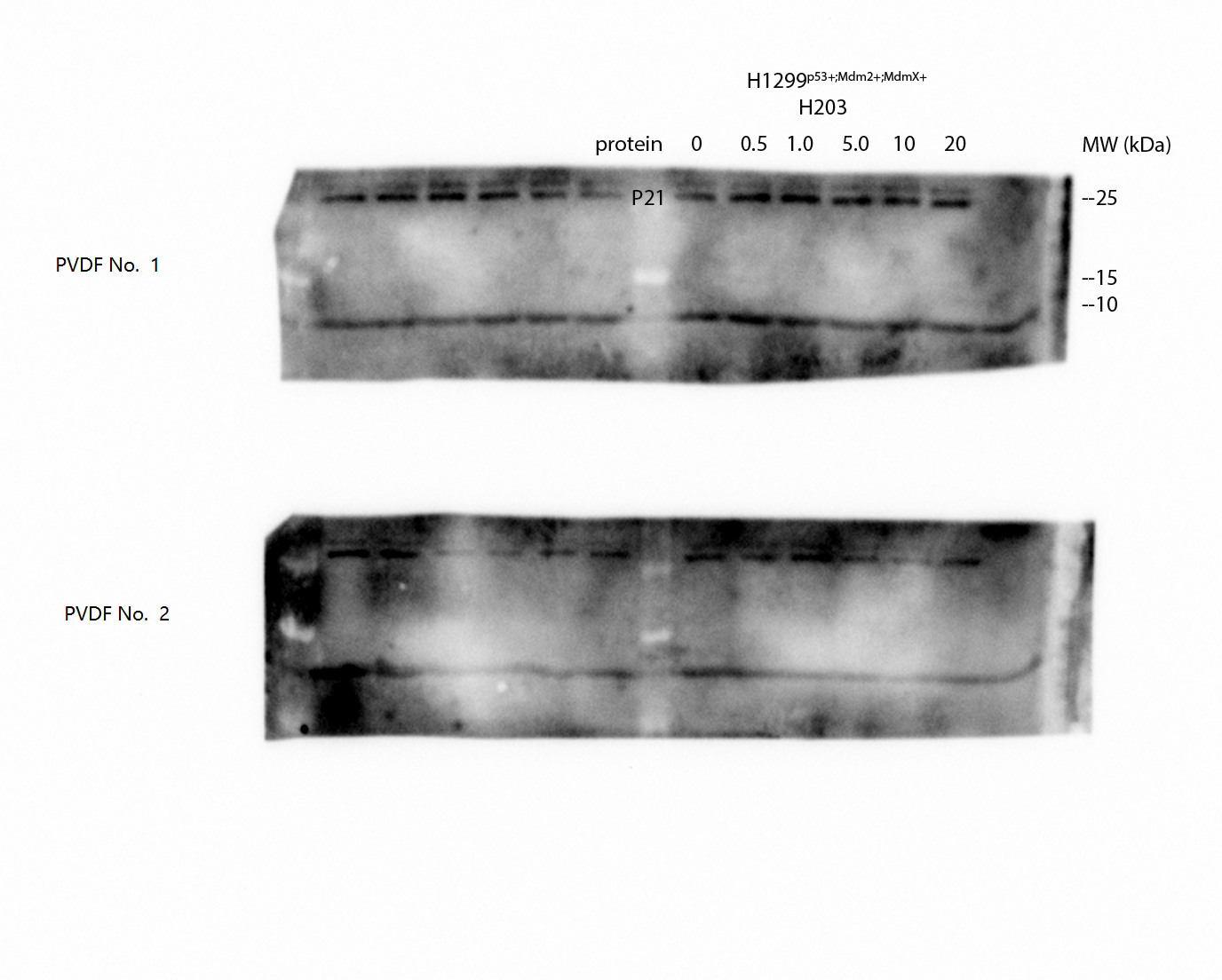

Supplement: Supplementary file 3 — Source Data [file 41467_2022_28721_MOESM3_ESM.zip › Source data/Source data for Supplementary Fig18/p21/Supplementary Fig18a_P21_H203.Tif]

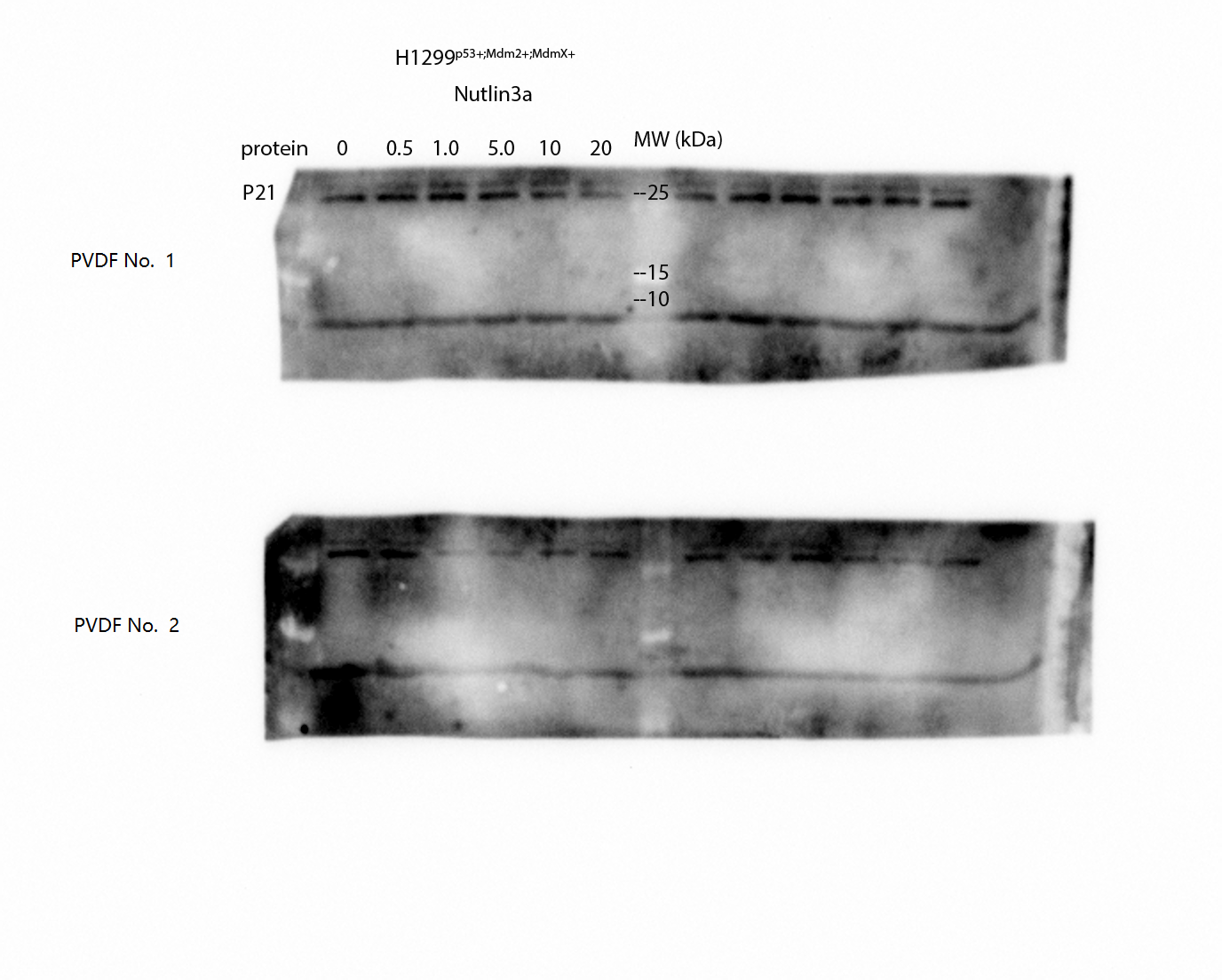

Supplement: Supplementary file 3 — Source Data [file 41467_2022_28721_MOESM3_ESM.zip › Source data/Source data for Supplementary Fig18/p21/Supplementary Fig18a_P21_nutlin3a.Tif]

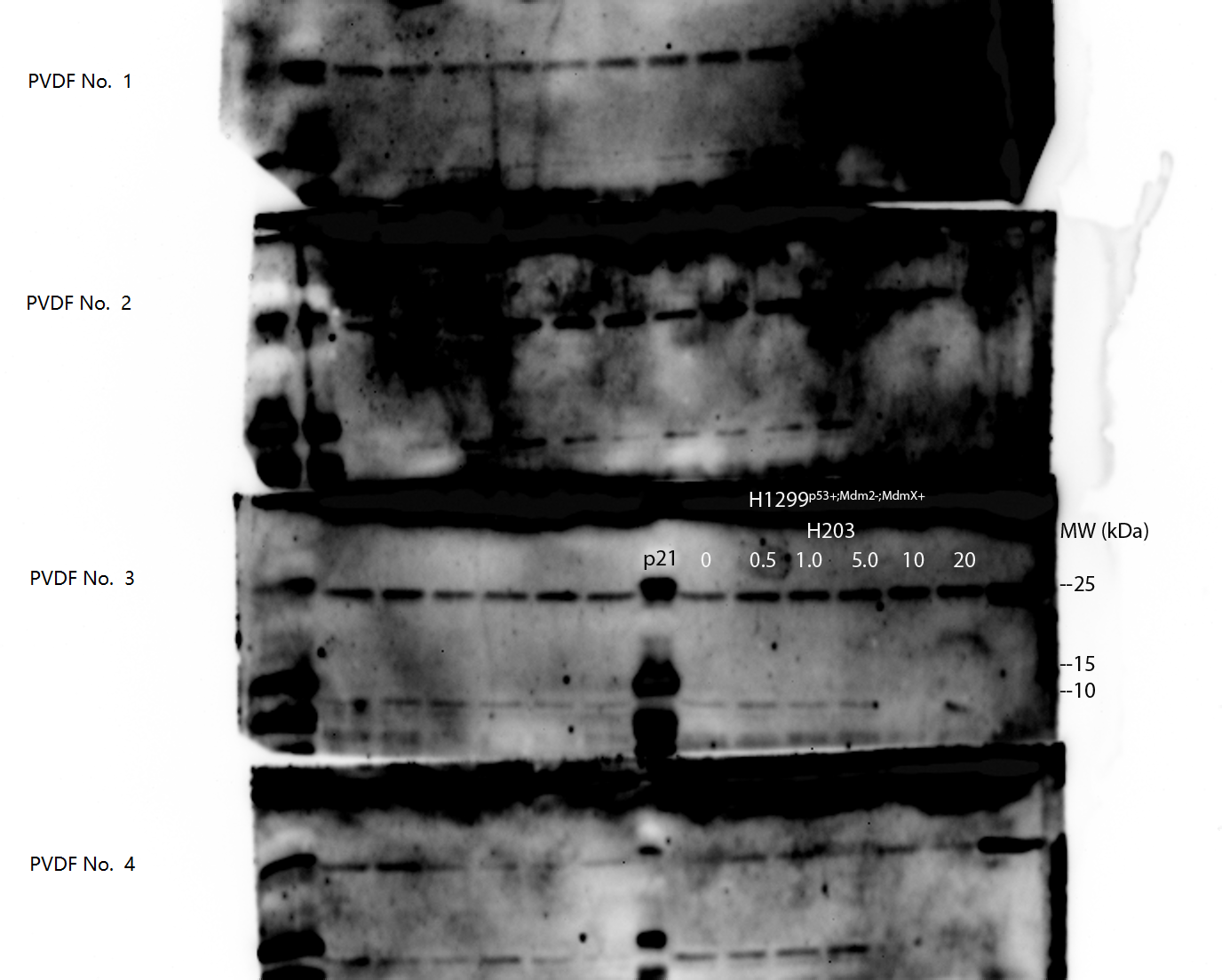

Supplement: Supplementary file 3 — Source Data [file 41467_2022_28721_MOESM3_ESM.zip › Source data/Source data for Supplementary Fig18/p21/Supplementary Fig18b_P21_H203.Tif]

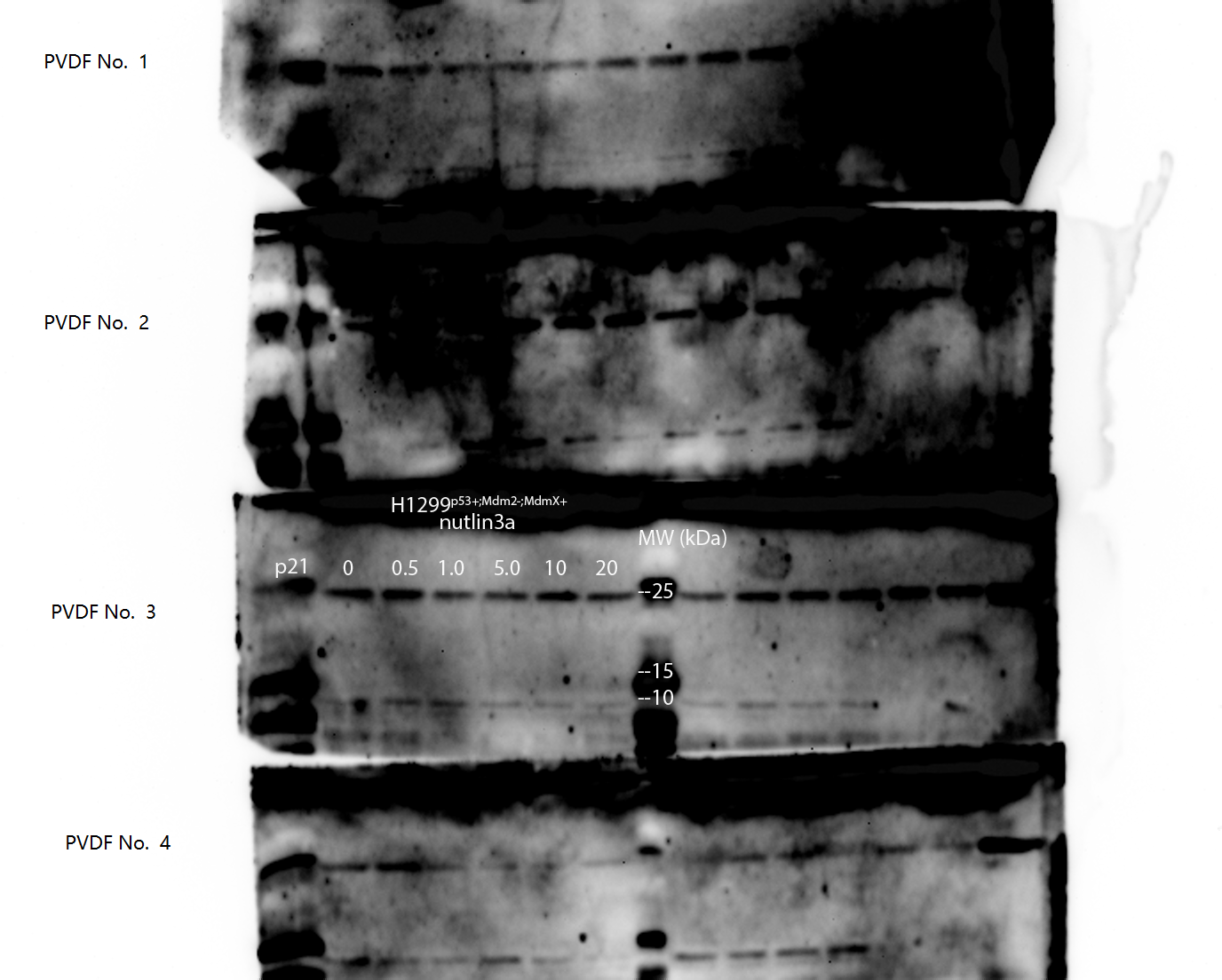

Supplement: Supplementary file 3 — Source Data [file 41467_2022_28721_MOESM3_ESM.zip › Source data/Source data for Supplementary Fig18/p21/Supplementary Fig18b_P21_nutlin3a.Tif]

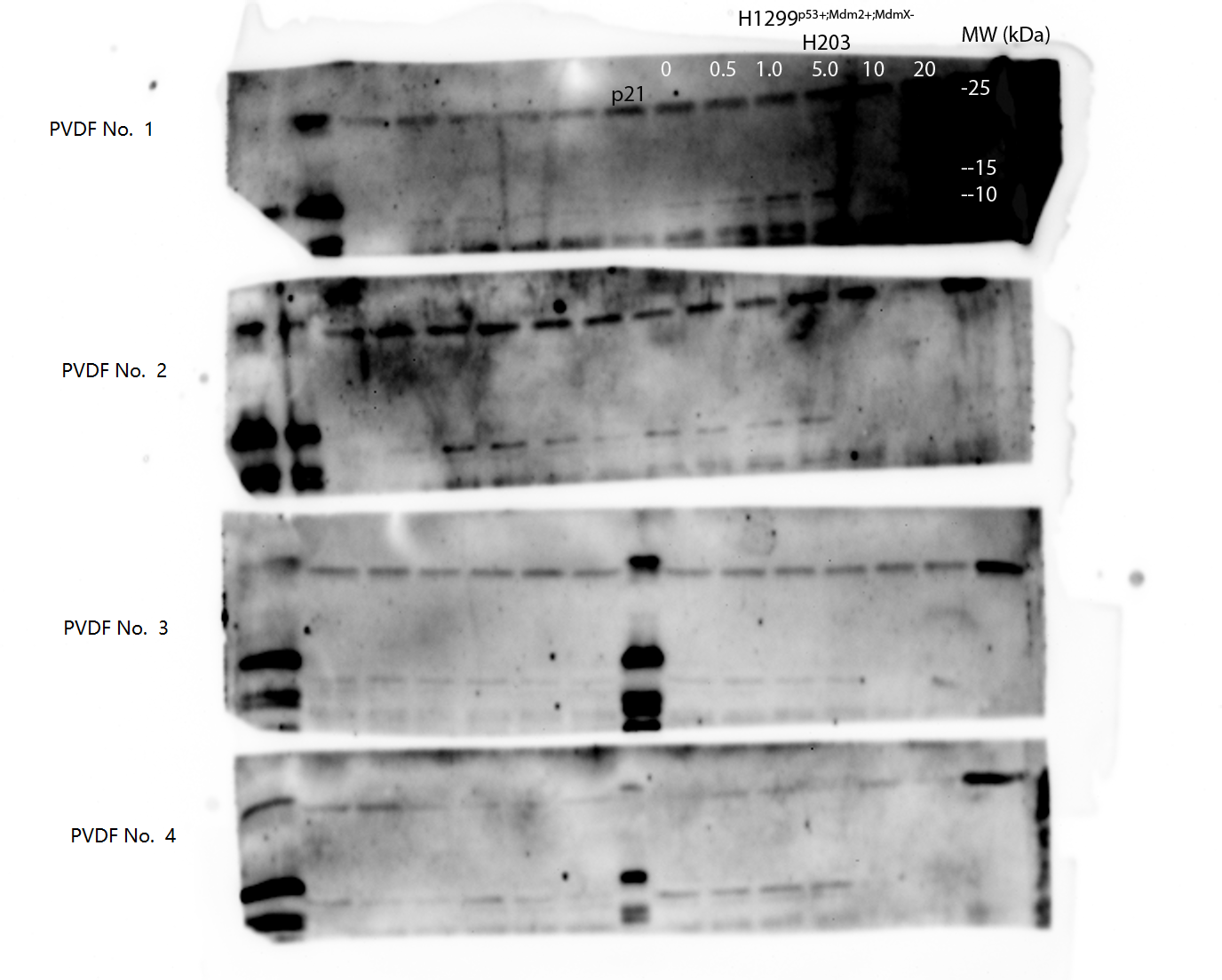

Supplement: Supplementary file 3 — Source Data [file 41467_2022_28721_MOESM3_ESM.zip › Source data/Source data for Supplementary Fig18/p21/Supplementary Fig18c_P21_H203.Tif]

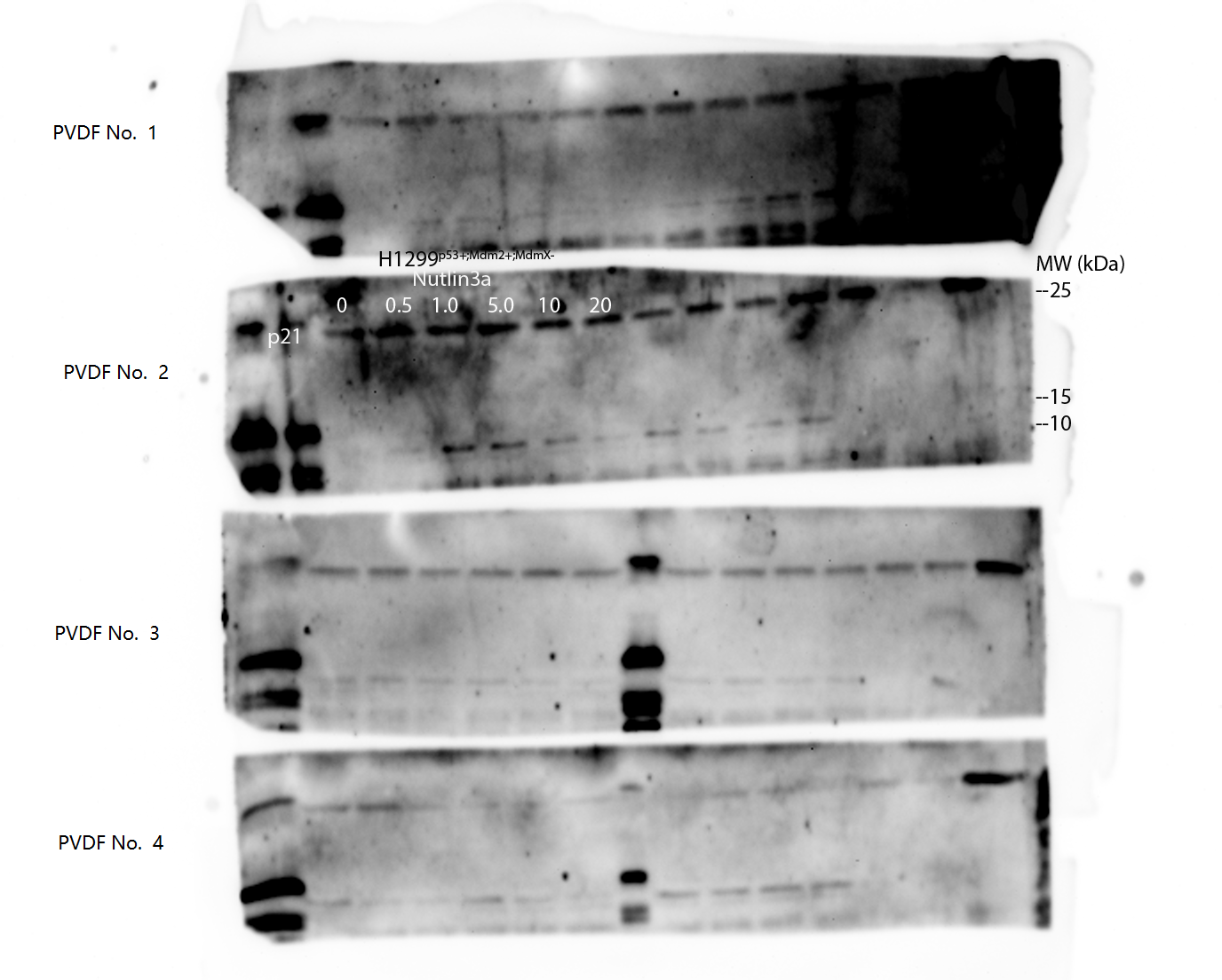

Supplement: Supplementary file 3 — Source Data [file 41467_2022_28721_MOESM3_ESM.zip › Source data/Source data for Supplementary Fig18/p21/Supplementary Fig18c_P21_nutlin3a.Tif]

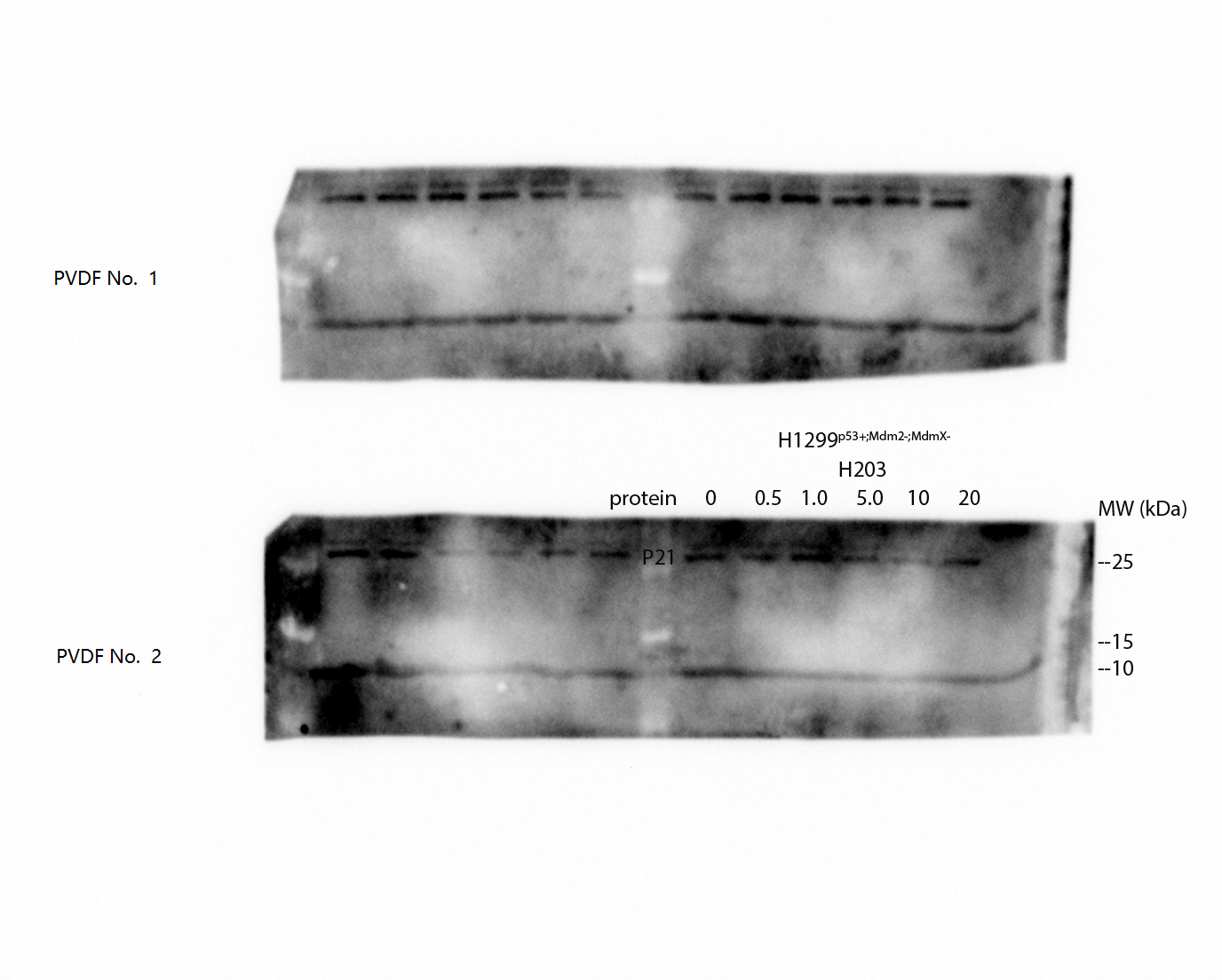

Supplement: Supplementary file 3 — Source Data [file 41467_2022_28721_MOESM3_ESM.zip › Source data/Source data for Supplementary Fig18/p21/Supplementary Fig18d_P21_H203.Tif]

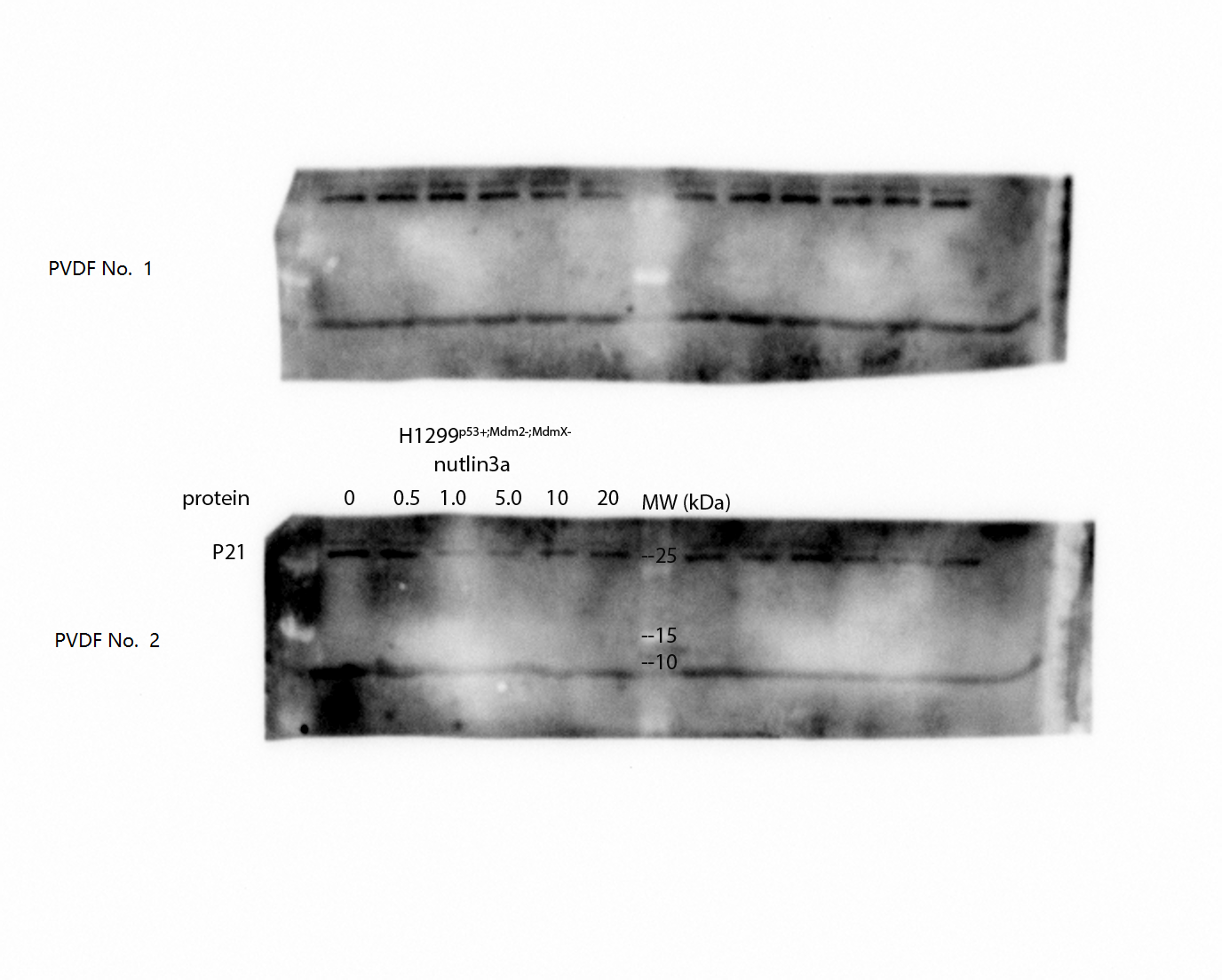

Supplement: Supplementary file 3 — Source Data [file 41467_2022_28721_MOESM3_ESM.zip › Source data/Source data for Supplementary Fig18/p21/Supplementary Fig18d_P21_nutlin3a.Tif]

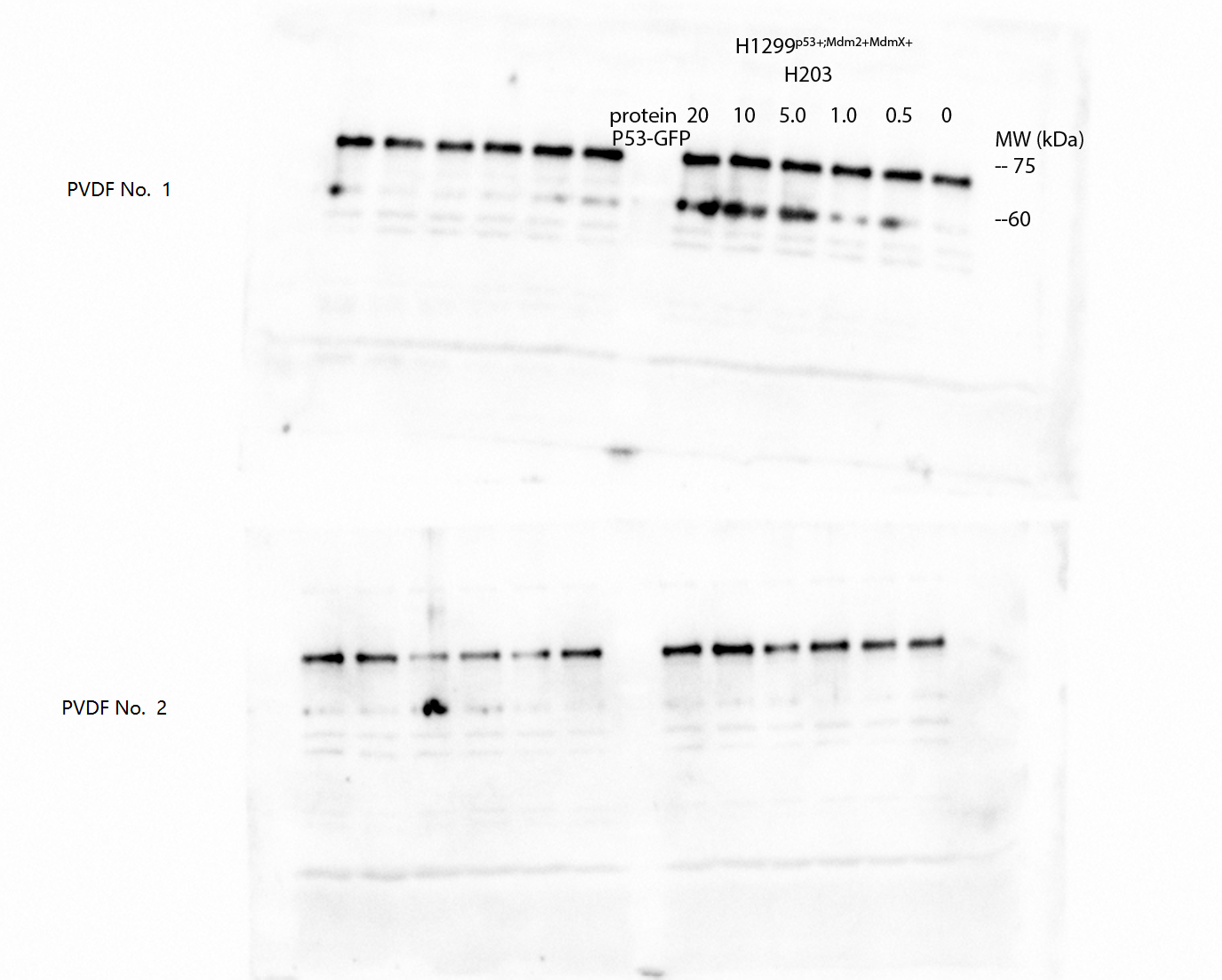

Supplement: Supplementary file 3 — Source Data [file 41467_2022_28721_MOESM3_ESM.zip › Source data/Source data for Supplementary Fig18/p53/Supplementary Fig18a_P53_H203.Tif]

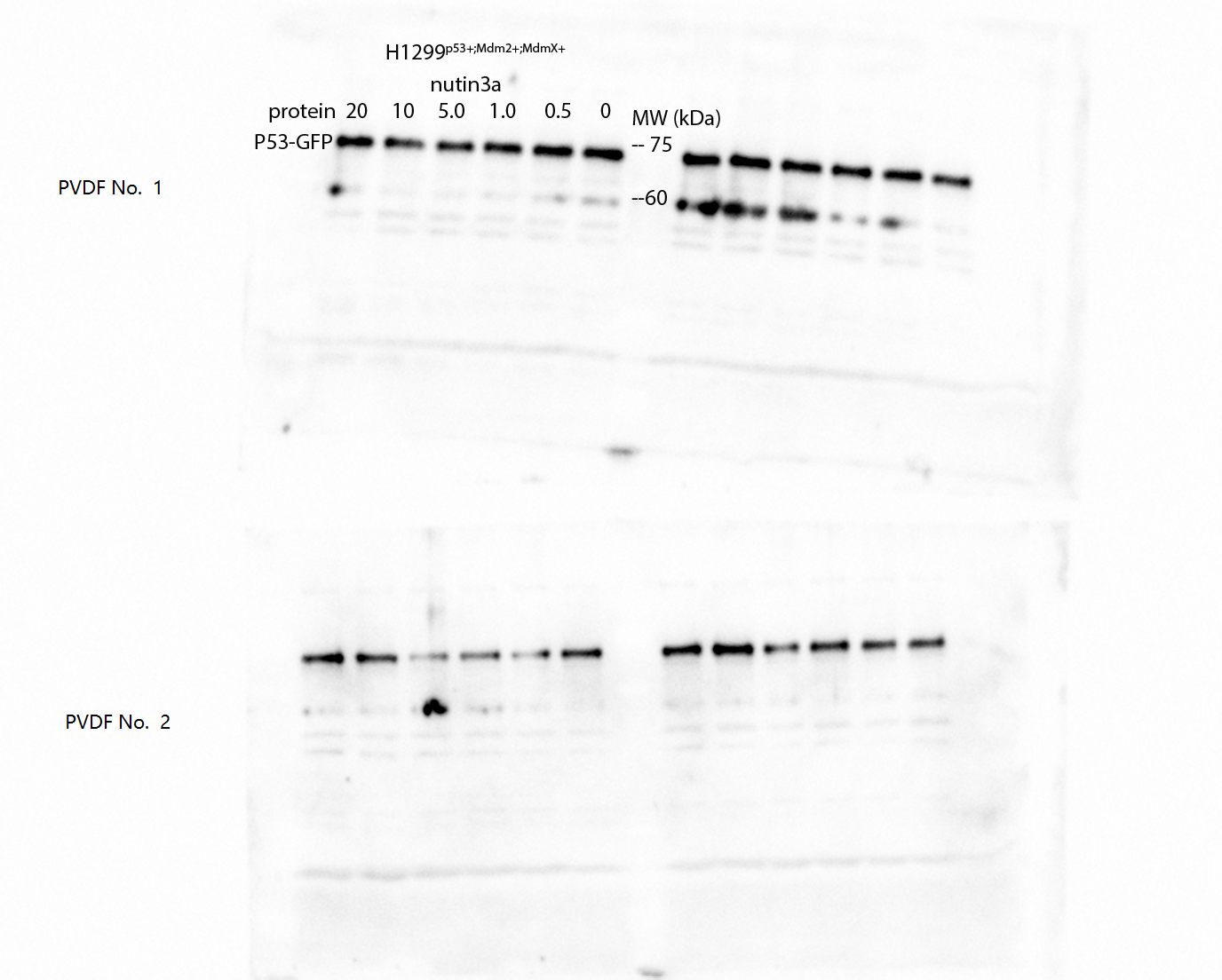

Supplement: Supplementary file 3 — Source Data [file 41467_2022_28721_MOESM3_ESM.zip › Source data/Source data for Supplementary Fig18/p53/Supplementary Fig18a_P53_nutlin3a.Tif]

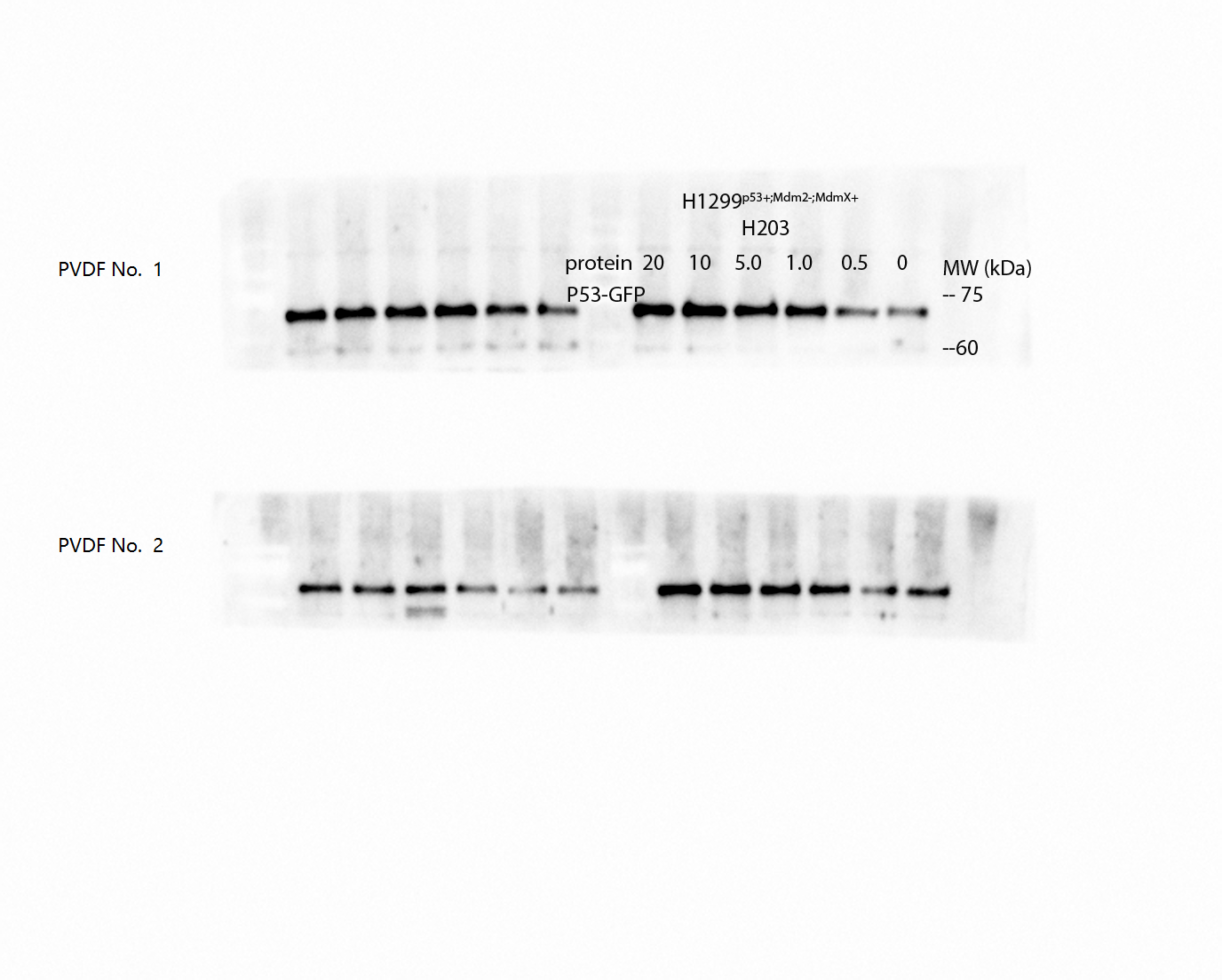

Supplement: Supplementary file 3 — Source Data [file 41467_2022_28721_MOESM3_ESM.zip › Source data/Source data for Supplementary Fig18/p53/Supplementary Fig18b_P53_H203.Tif]

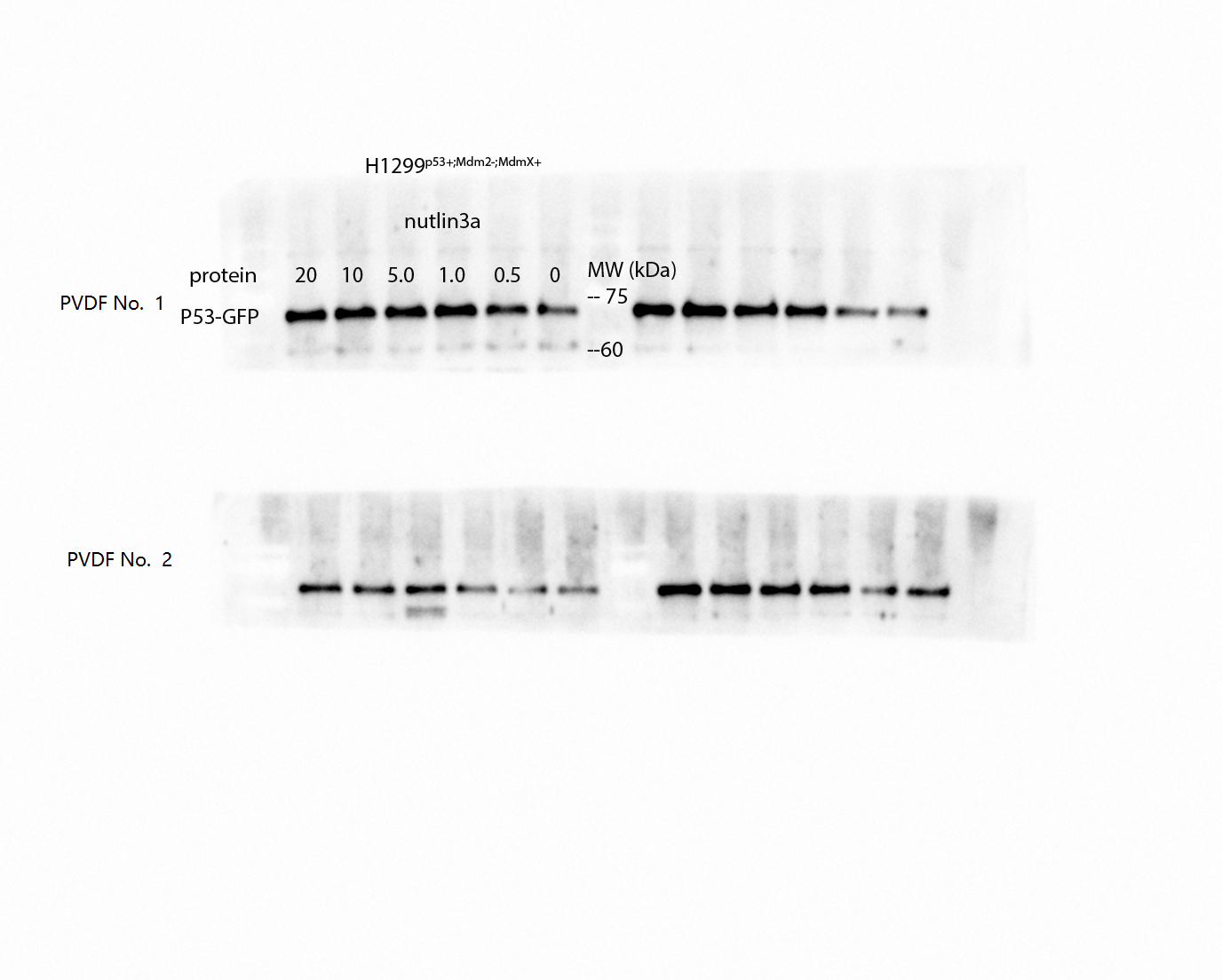

Supplement: Supplementary file 3 — Source Data [file 41467_2022_28721_MOESM3_ESM.zip › Source data/Source data for Supplementary Fig18/p53/Supplementary Fig18b_P53_nutlin3a.Tif]

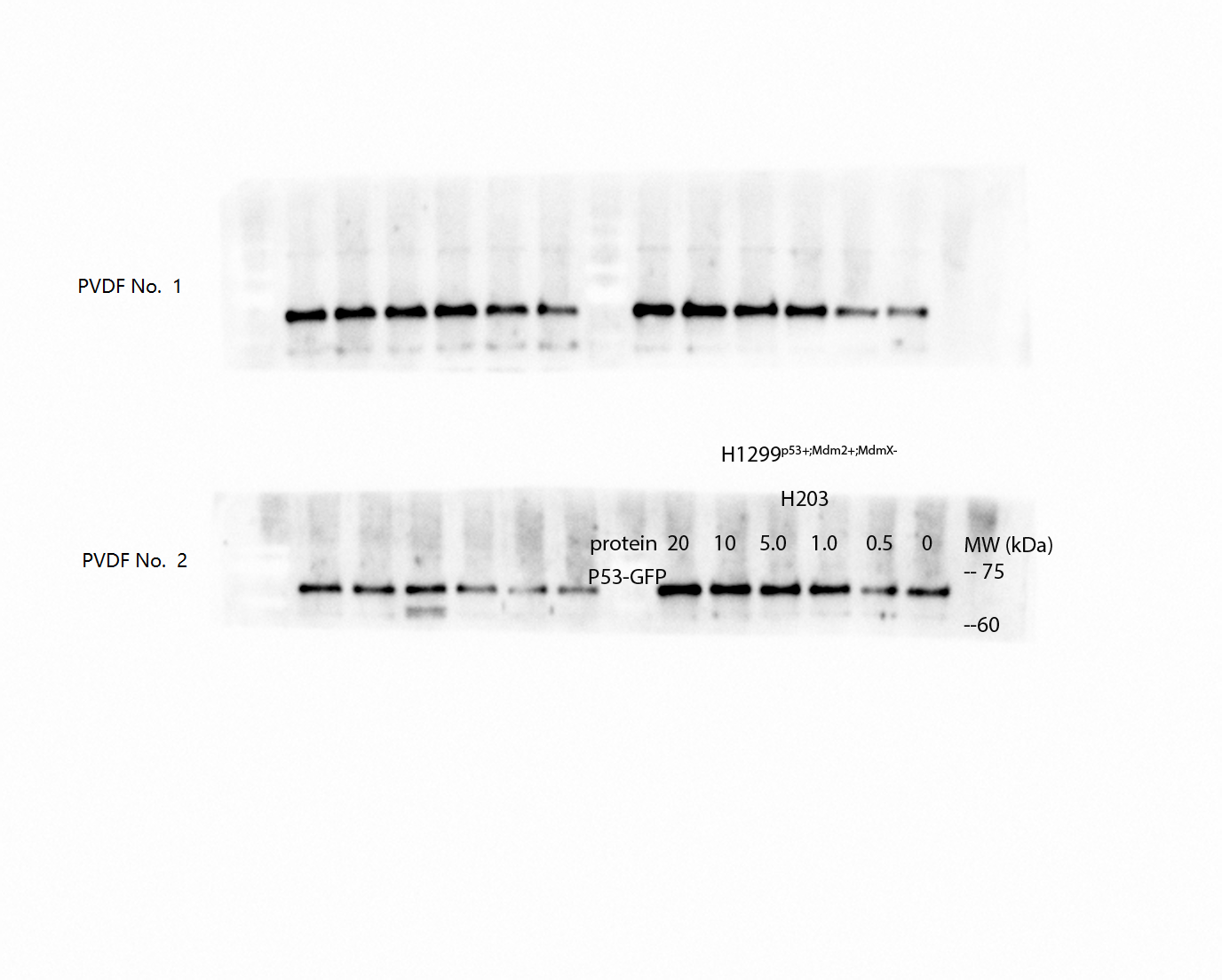

Supplement: Supplementary file 3 — Source Data [file 41467_2022_28721_MOESM3_ESM.zip › Source data/Source data for Supplementary Fig18/p53/Supplementary Fig18c_P53_H203.Tif]

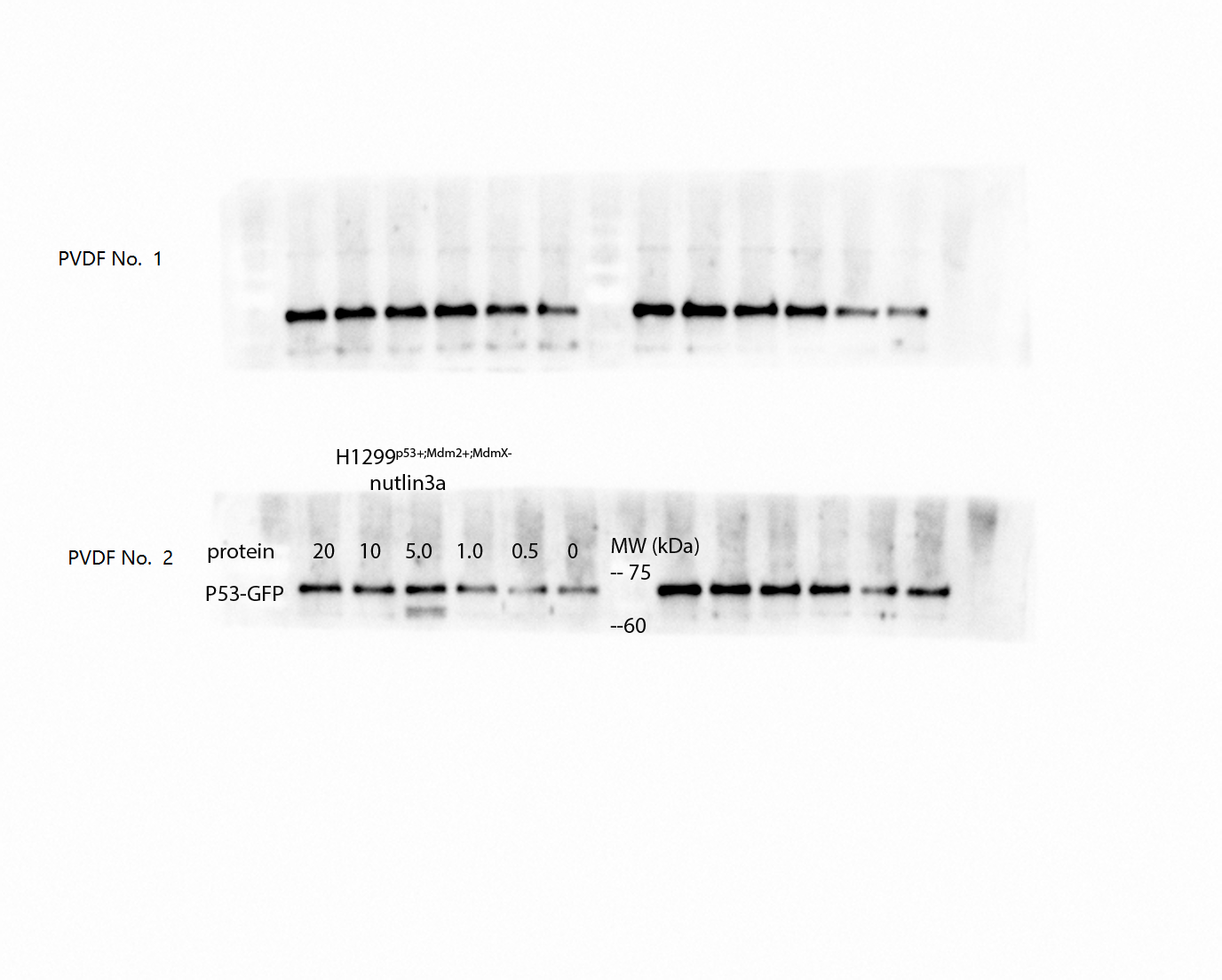

Supplement: Supplementary file 3 — Source Data [file 41467_2022_28721_MOESM3_ESM.zip › Source data/Source data for Supplementary Fig18/p53/Supplementary Fig18c_P53_nutlin3a.Tif]

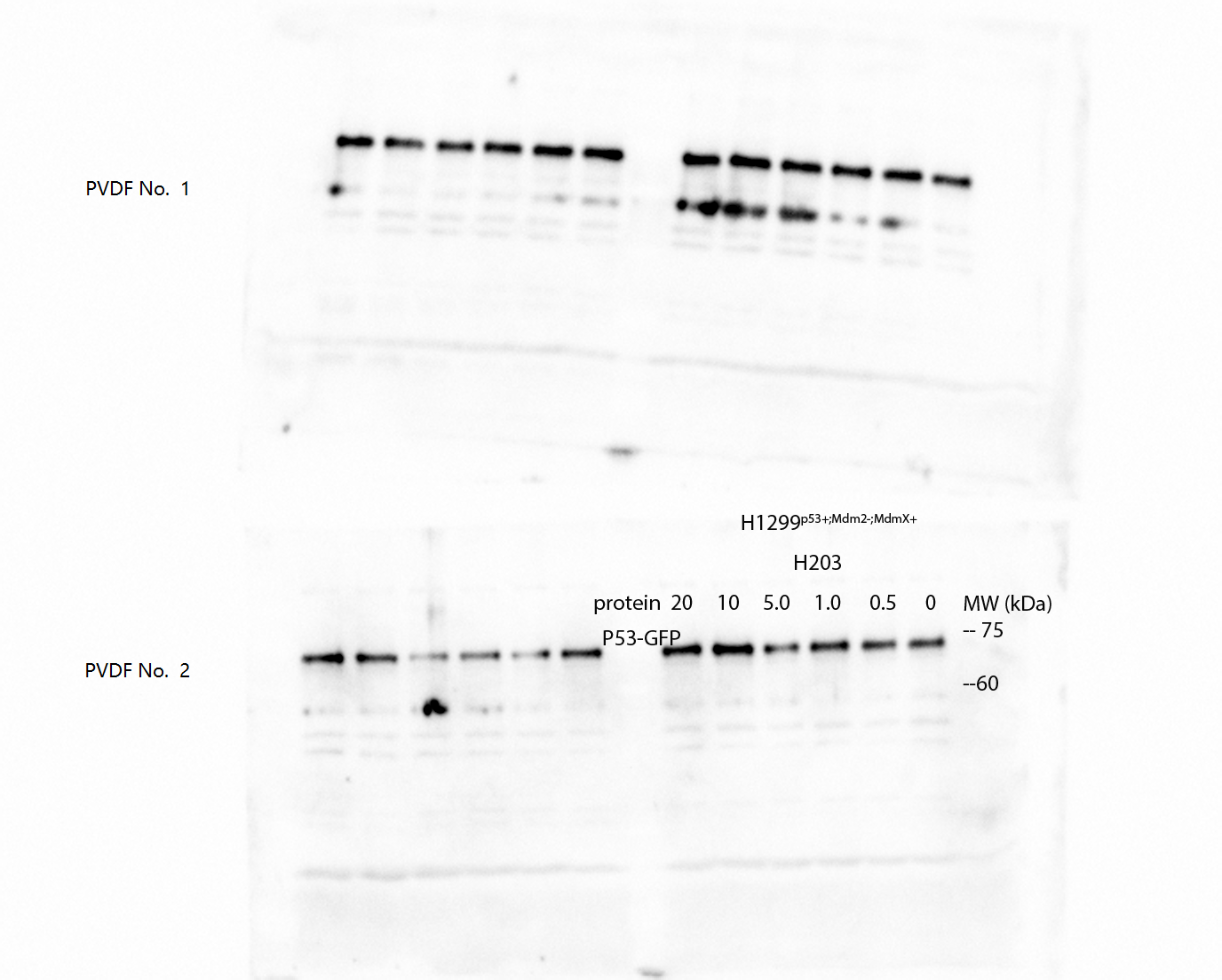

Supplement: Supplementary file 3 — Source Data [file 41467_2022_28721_MOESM3_ESM.zip › Source data/Source data for Supplementary Fig18/p53/Supplementary Fig18d_P53_H203.Tif]

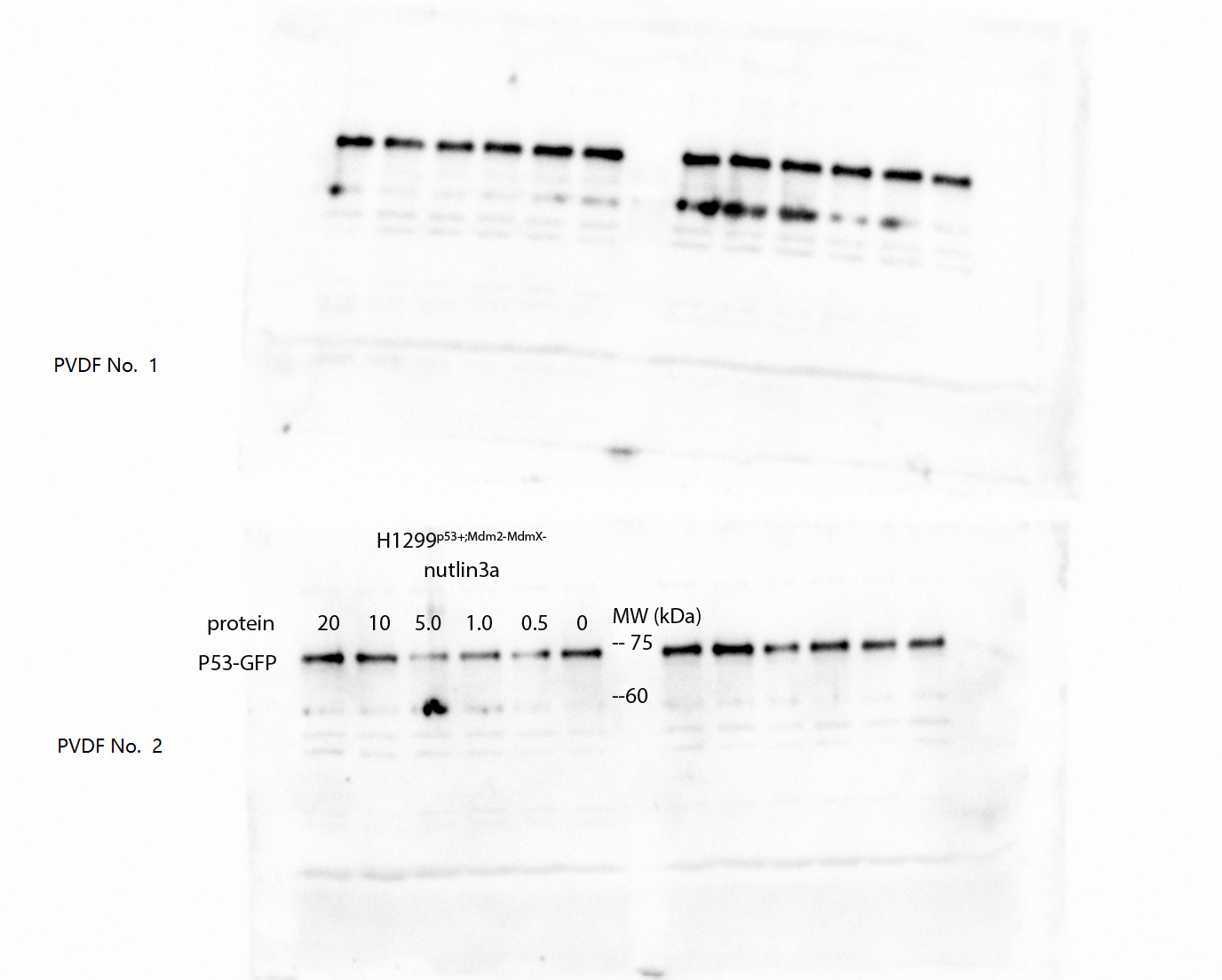

Supplement: Supplementary file 3 — Source Data [file 41467_2022_28721_MOESM3_ESM.zip › Source data/Source data for Supplementary Fig18/p53/Supplementary Fig18d_P53_nutlin3a.Tif]

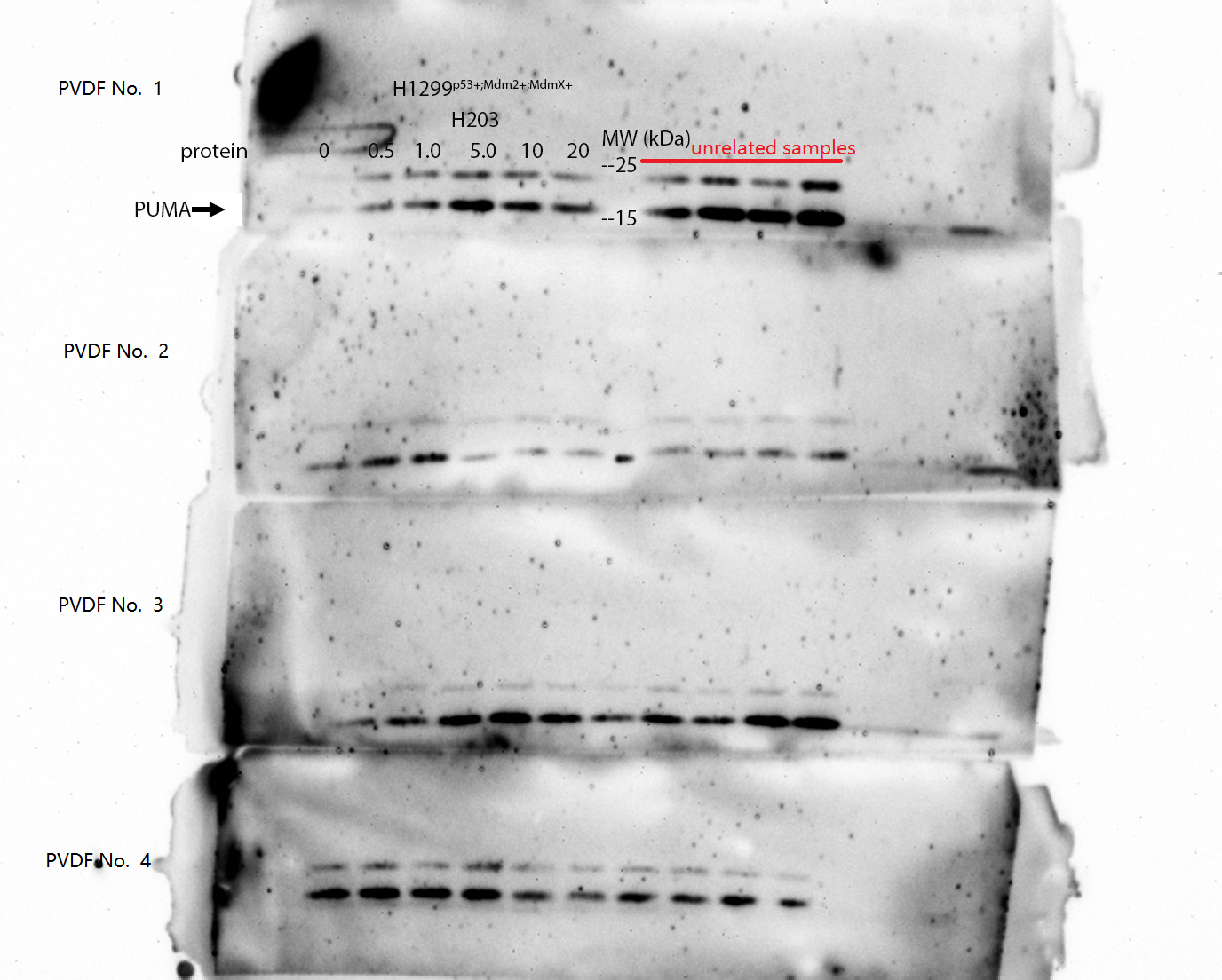

Supplement: Supplementary file 3 — Source Data [file 41467_2022_28721_MOESM3_ESM.zip › Source data/Source data for Supplementary Fig18/PUMA/Supplementary Fig18a_PUMA_H203.Tif]

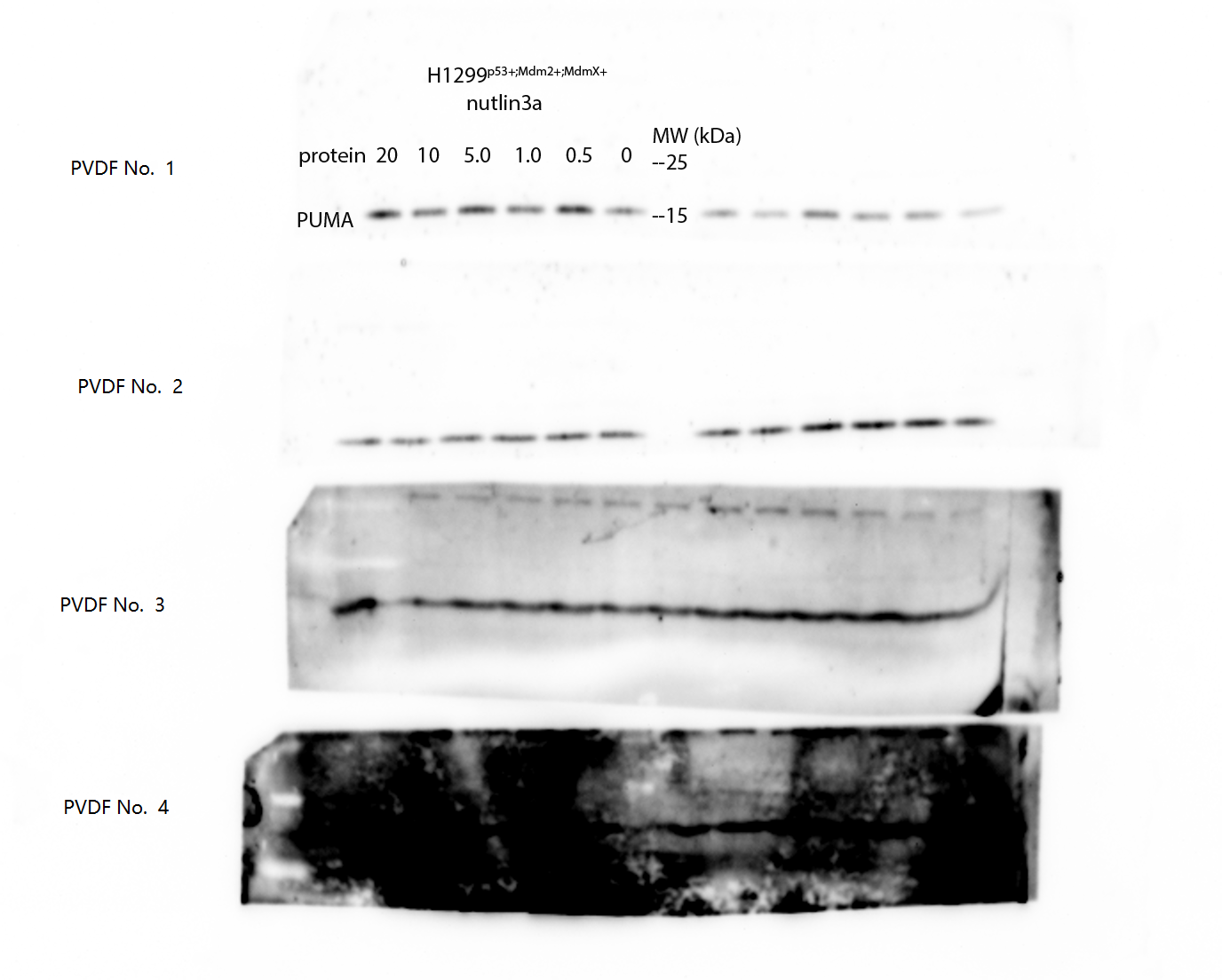

Supplement: Supplementary file 3 — Source Data [file 41467_2022_28721_MOESM3_ESM.zip › Source data/Source data for Supplementary Fig18/PUMA/Supplementary Fig18a_PUMA_Nutlin3a.Tif]

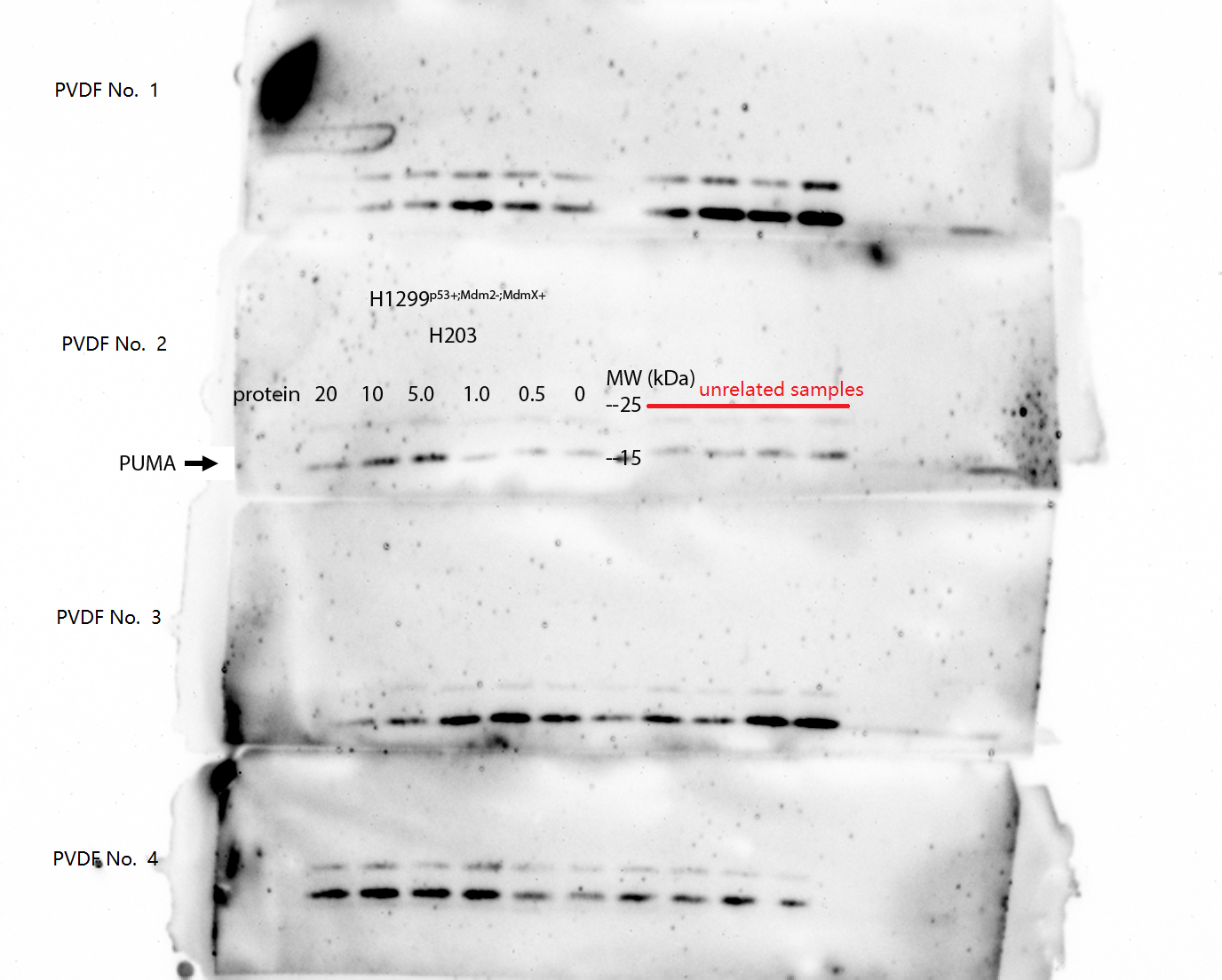

Supplement: Supplementary file 3 — Source Data [file 41467_2022_28721_MOESM3_ESM.zip › Source data/Source data for Supplementary Fig18/PUMA/Supplementary Fig18b_PUMA_H203.Tif]

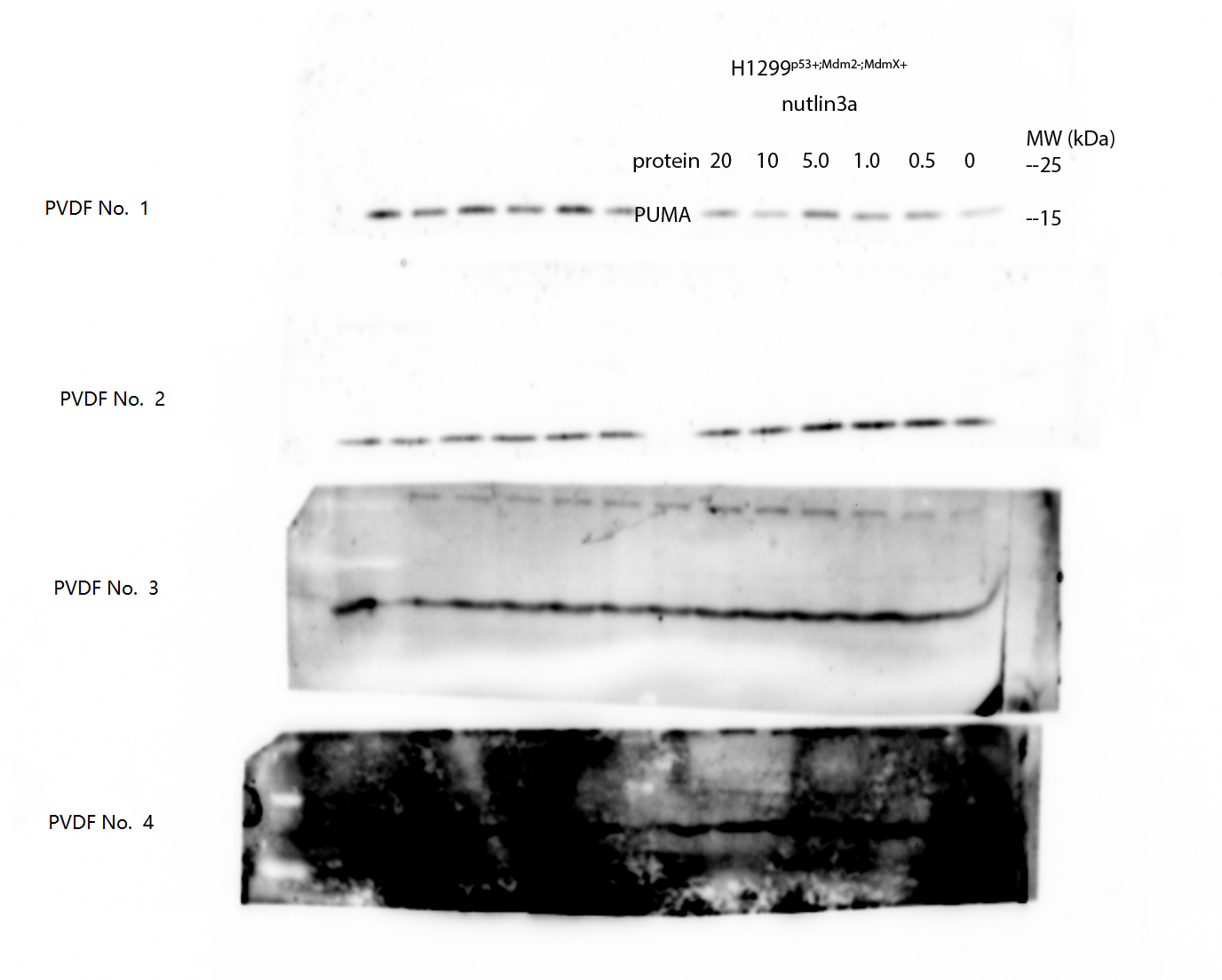

Supplement: Supplementary file 3 — Source Data [file 41467_2022_28721_MOESM3_ESM.zip › Source data/Source data for Supplementary Fig18/PUMA/Supplementary Fig18b_PUMA_nutlin3a.Tif]

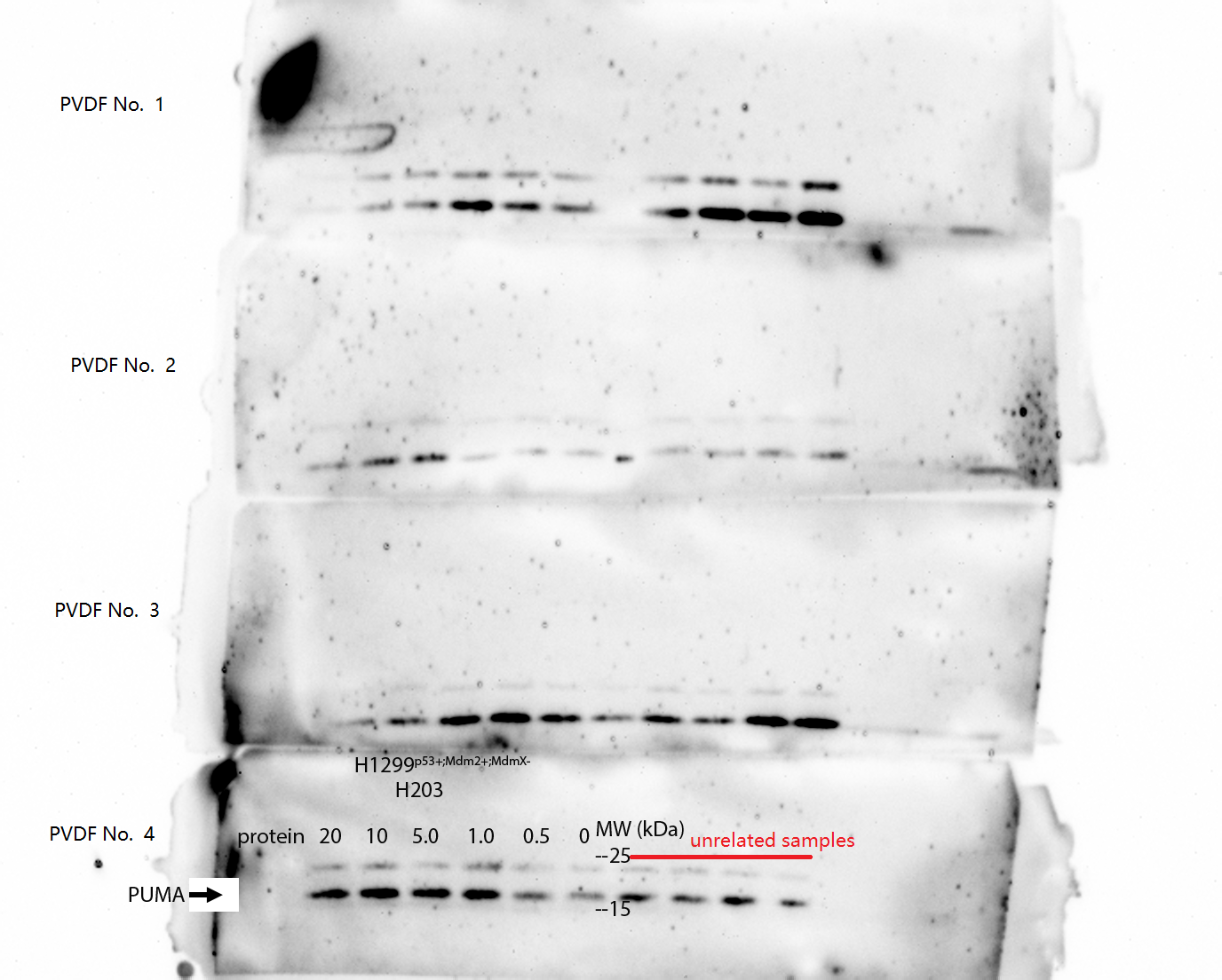

Supplement: Supplementary file 3 — Source Data [file 41467_2022_28721_MOESM3_ESM.zip › Source data/Source data for Supplementary Fig18/PUMA/Supplementary Fig18c_PUMA_H203.Tif]

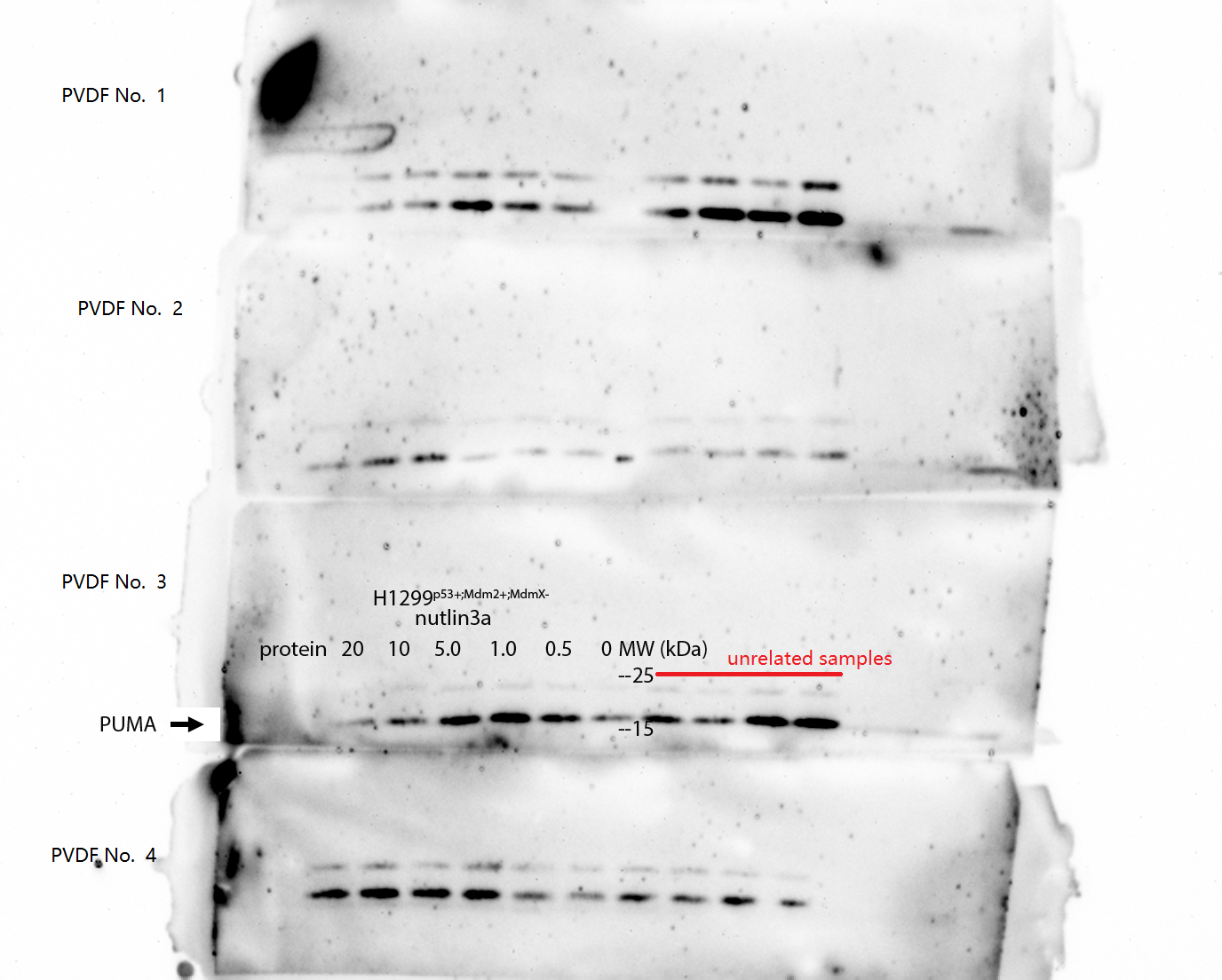

Supplement: Supplementary file 3 — Source Data [file 41467_2022_28721_MOESM3_ESM.zip › Source data/Source data for Supplementary Fig18/PUMA/Supplementary Fig18c_PUMA_Nutlin3a.Tif]

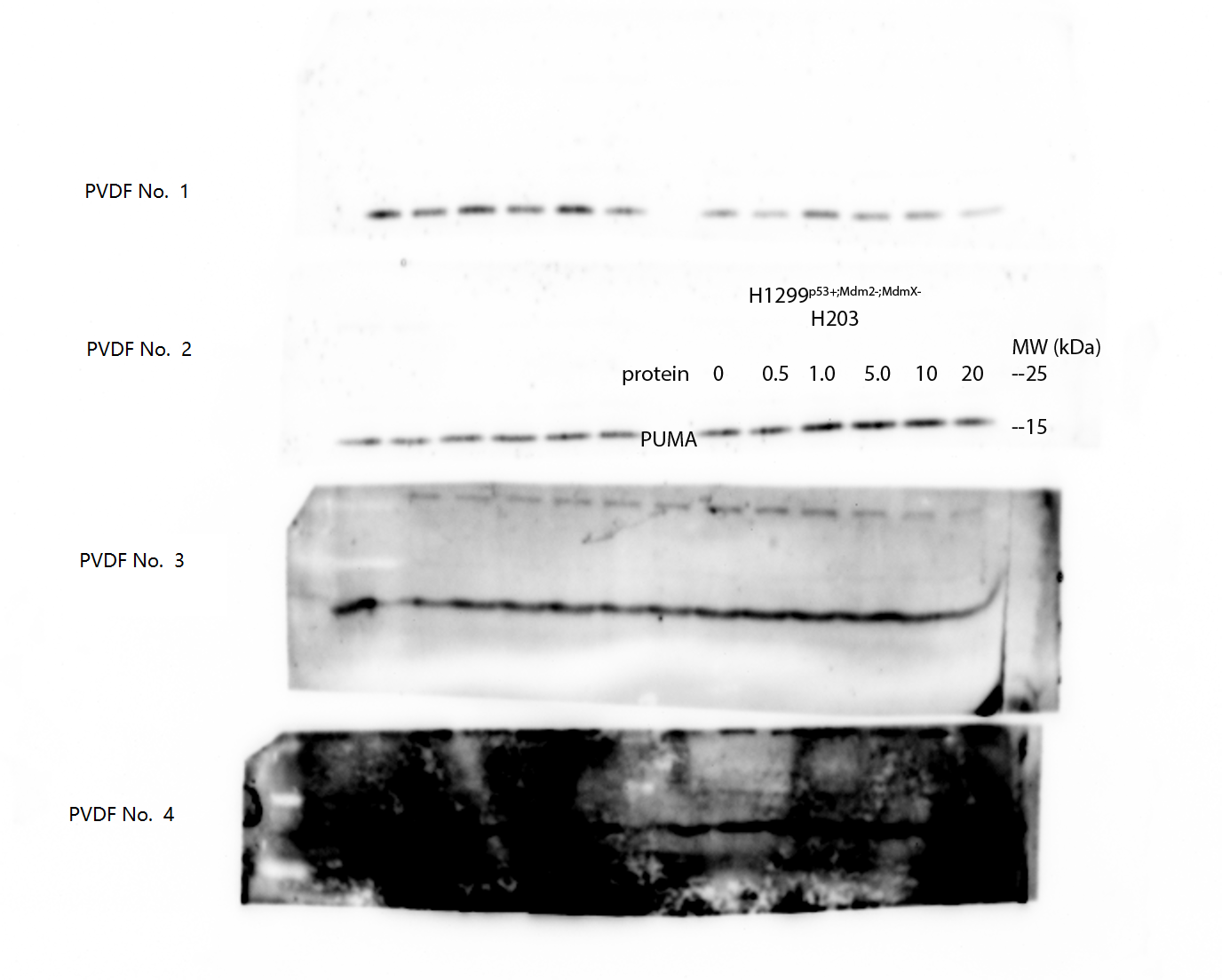

Supplement: Supplementary file 3 — Source Data [file 41467_2022_28721_MOESM3_ESM.zip › Source data/Source data for Supplementary Fig18/PUMA/Supplementary Fig18d_PUMA_H203.Tif]

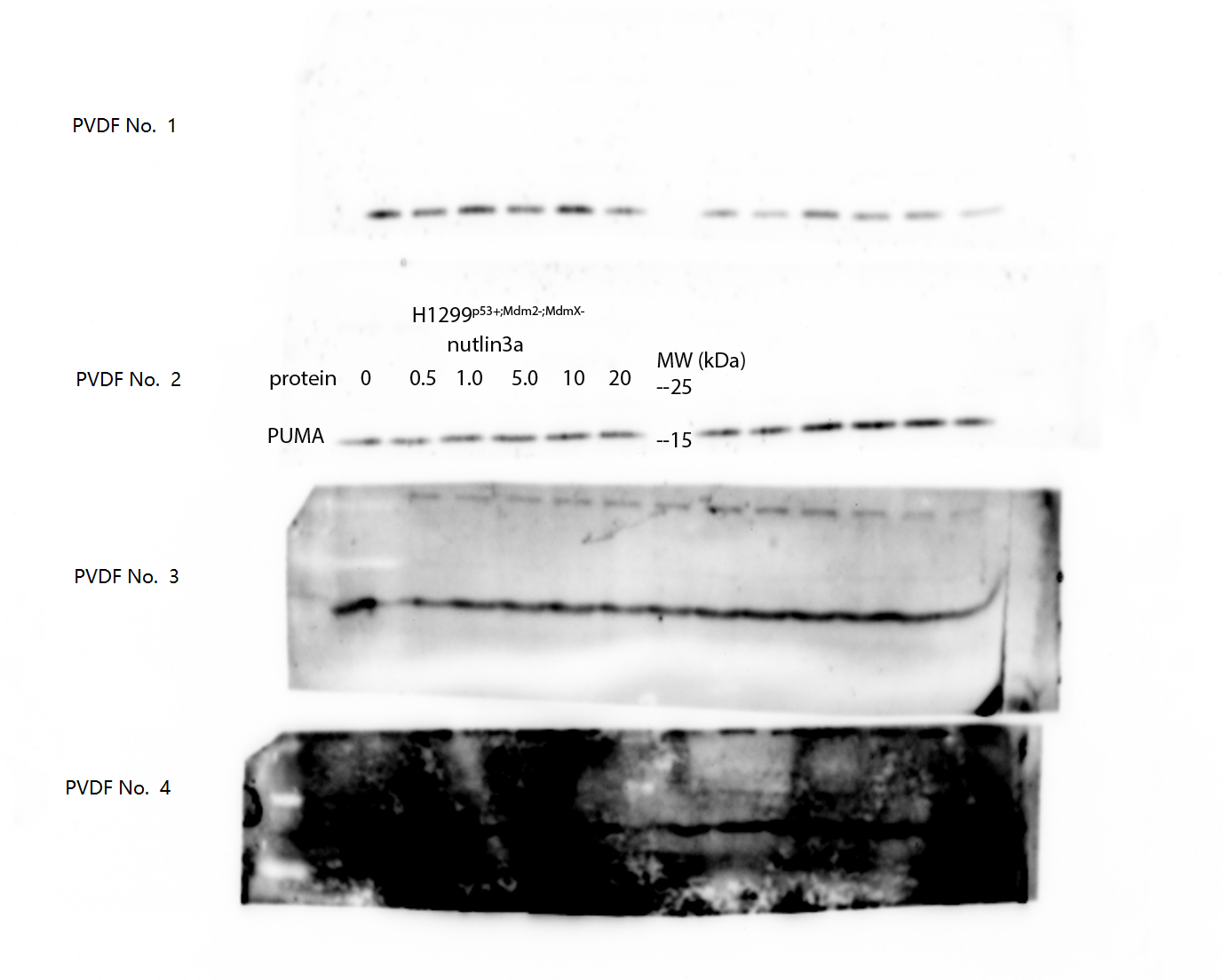

Supplement: Supplementary file 3 — Source Data [file 41467_2022_28721_MOESM3_ESM.zip › Source data/Source data for Supplementary Fig18/PUMA/Supplementary Fig18d_PUMA_nutlin3a.Tif]

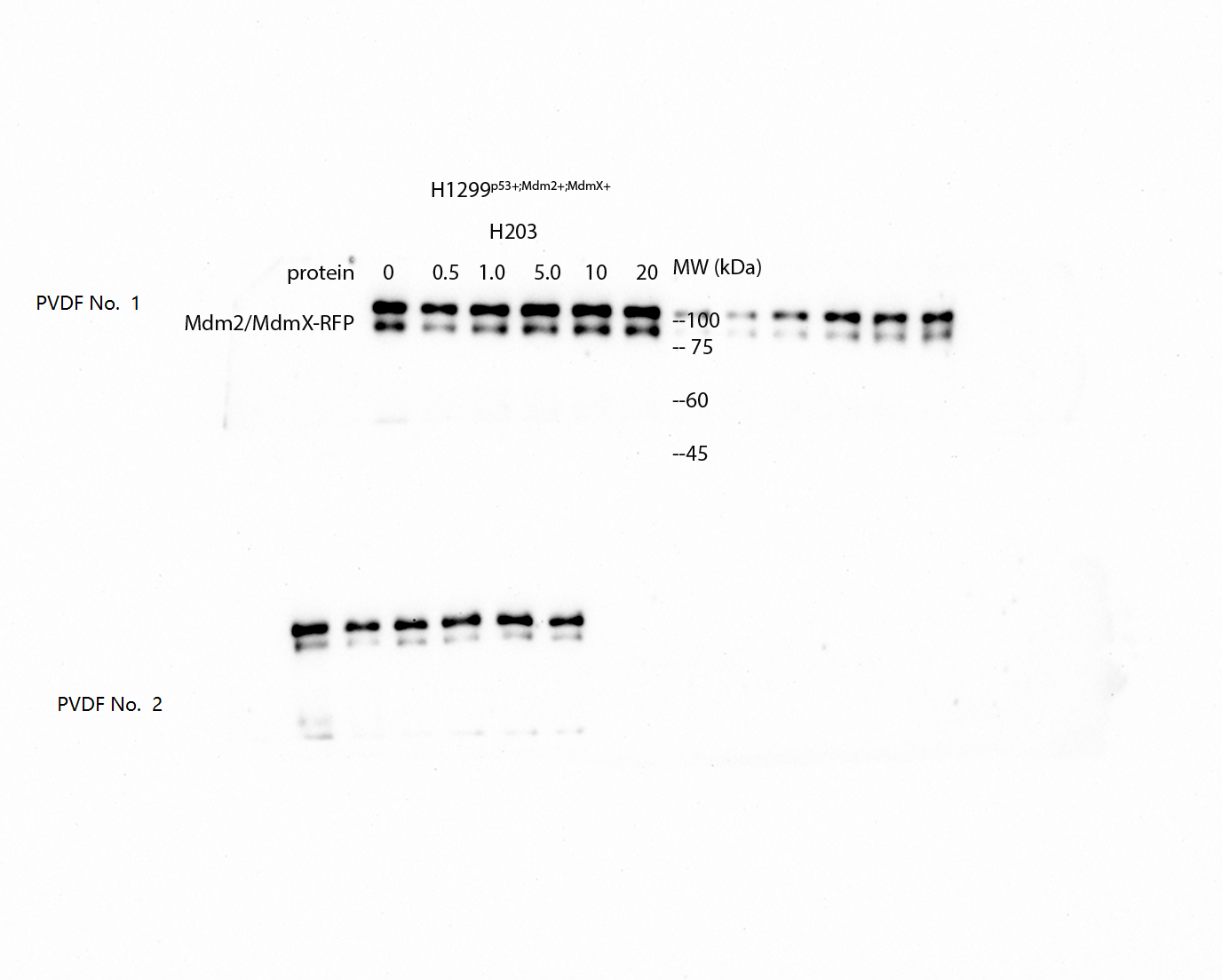

Supplement: Supplementary file 3 — Source Data [file 41467_2022_28721_MOESM3_ESM.zip › Source data/Source data for Supplementary Fig18/RFP/Supplementary Fig18a_Mdm2_MdmX_H203.Tif]

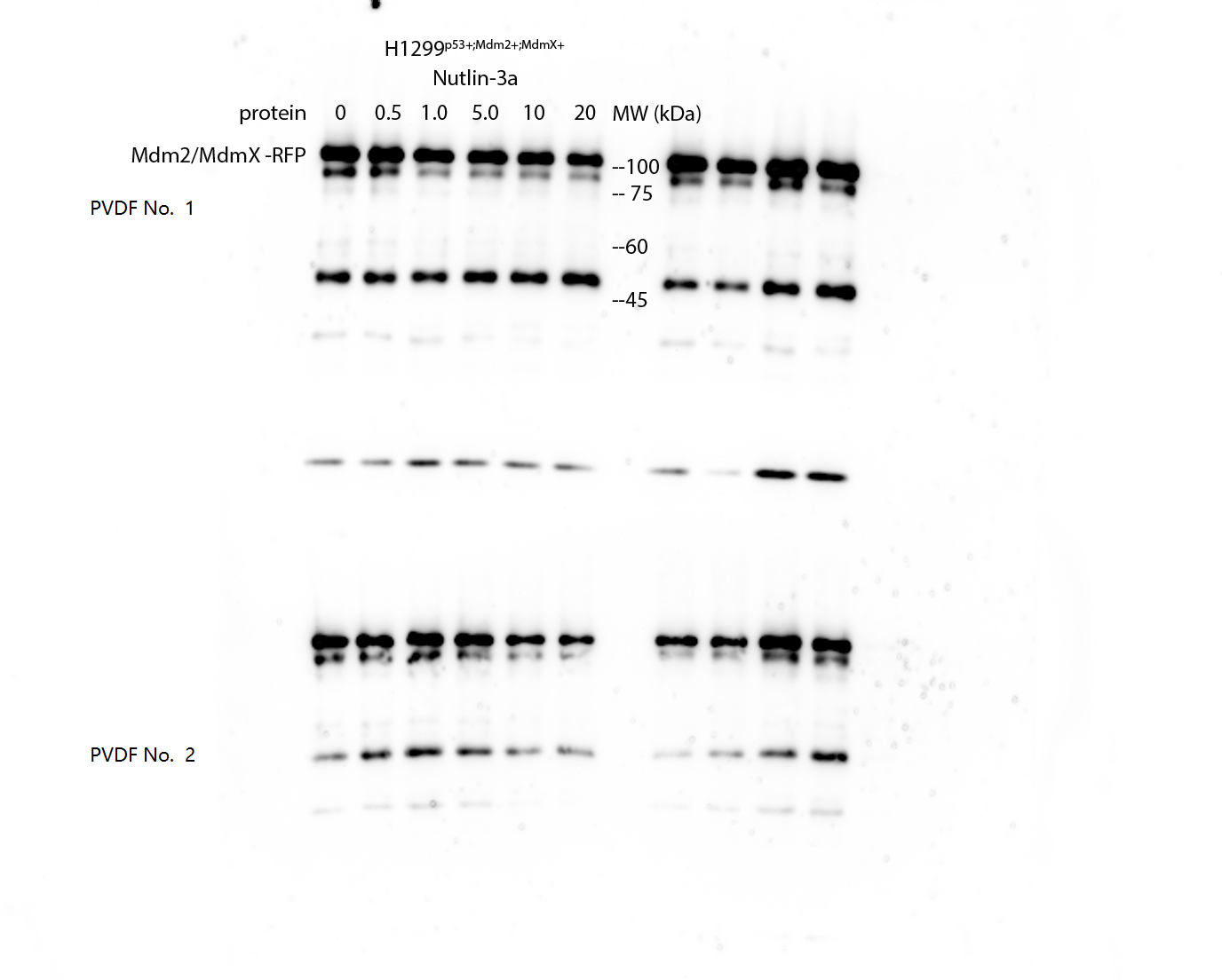

Supplement: Supplementary file 3 — Source Data [file 41467_2022_28721_MOESM3_ESM.zip › Source data/Source data for Supplementary Fig18/RFP/Supplementary Fig18a_Mdm2_MdmX_nutlin3a.Tif]

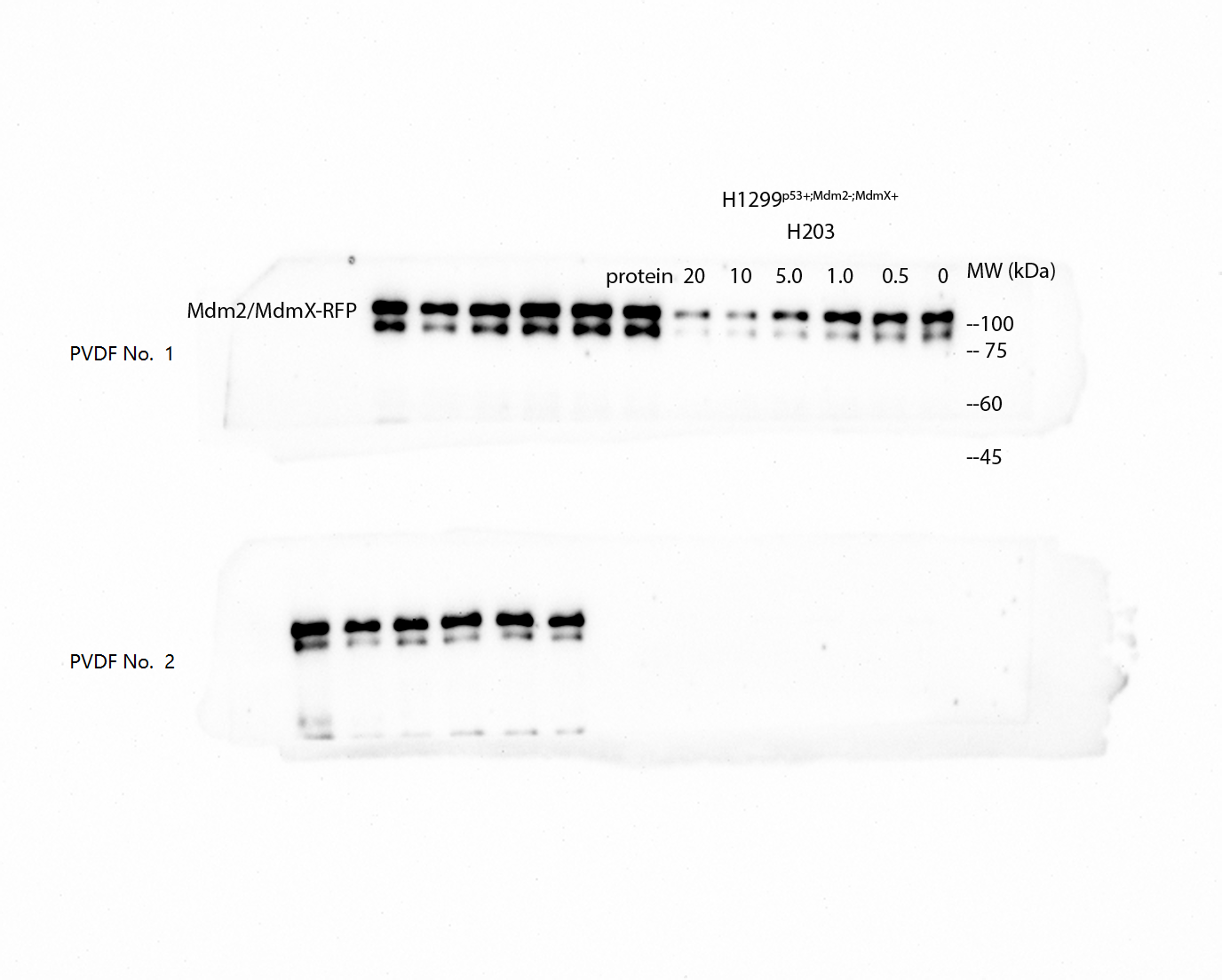

Supplement: Supplementary file 3 — Source Data [file 41467_2022_28721_MOESM3_ESM.zip › Source data/Source data for Supplementary Fig18/RFP/Supplementary Fig18b_Mdm2_MdmX_H203.Tif]

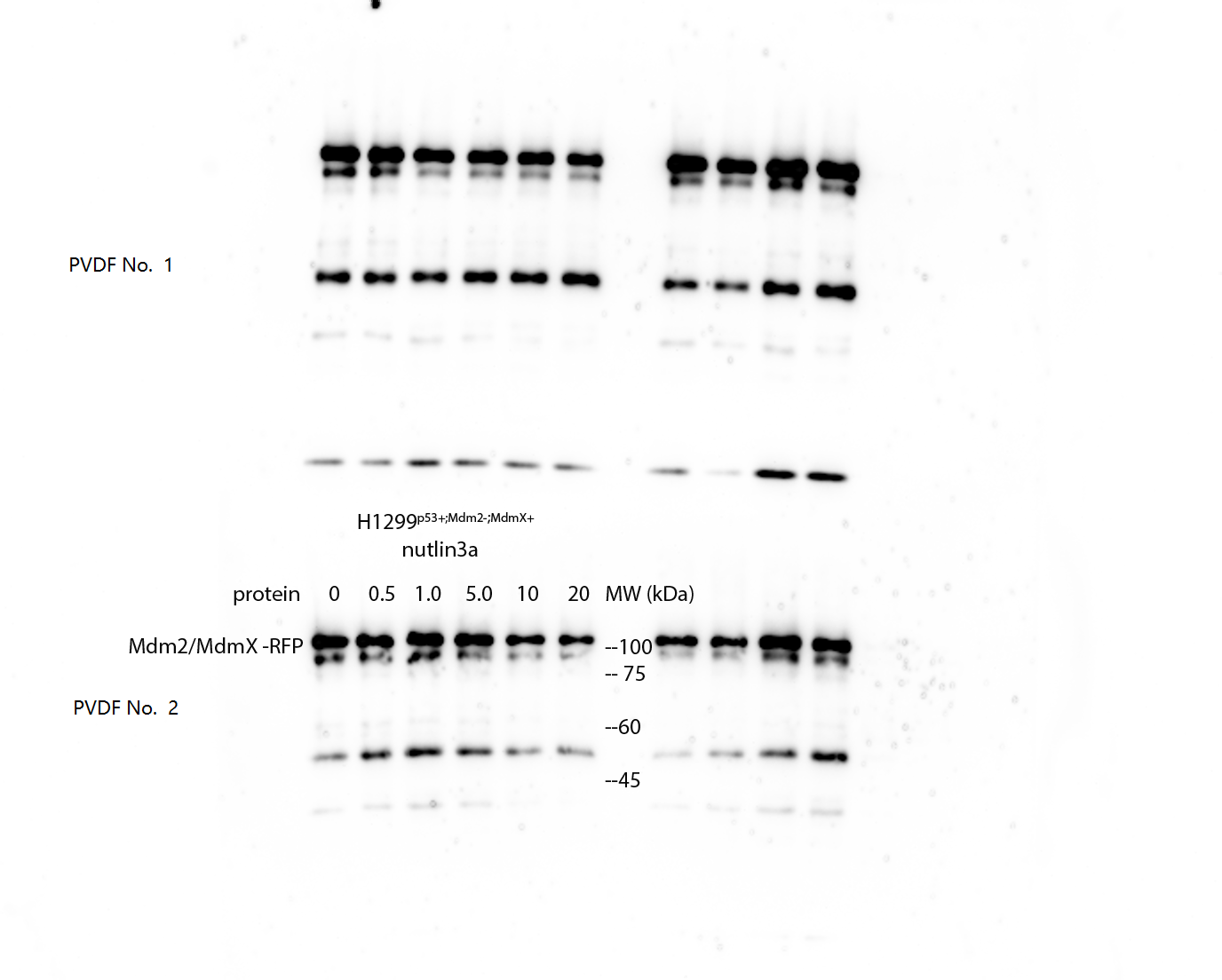

Supplement: Supplementary file 3 — Source Data [file 41467_2022_28721_MOESM3_ESM.zip › Source data/Source data for Supplementary Fig18/RFP/Supplementary Fig18b_Mdm2_MdmX_nutlin3a.Tif]

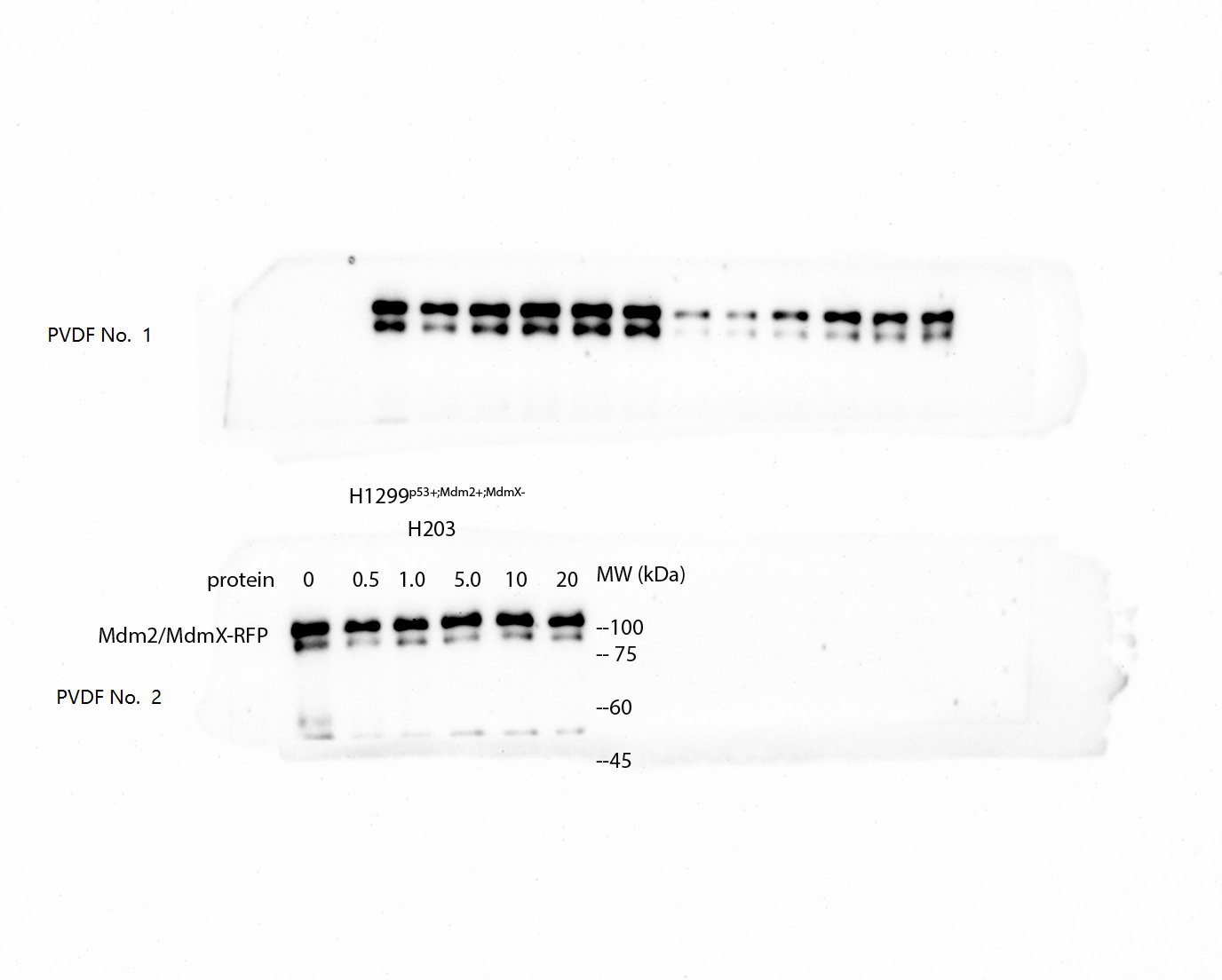

Supplement: Supplementary file 3 — Source Data [file 41467_2022_28721_MOESM3_ESM.zip › Source data/Source data for Supplementary Fig18/RFP/Supplementary Fig18c_Mdm2_MdmX_H203.Tif]

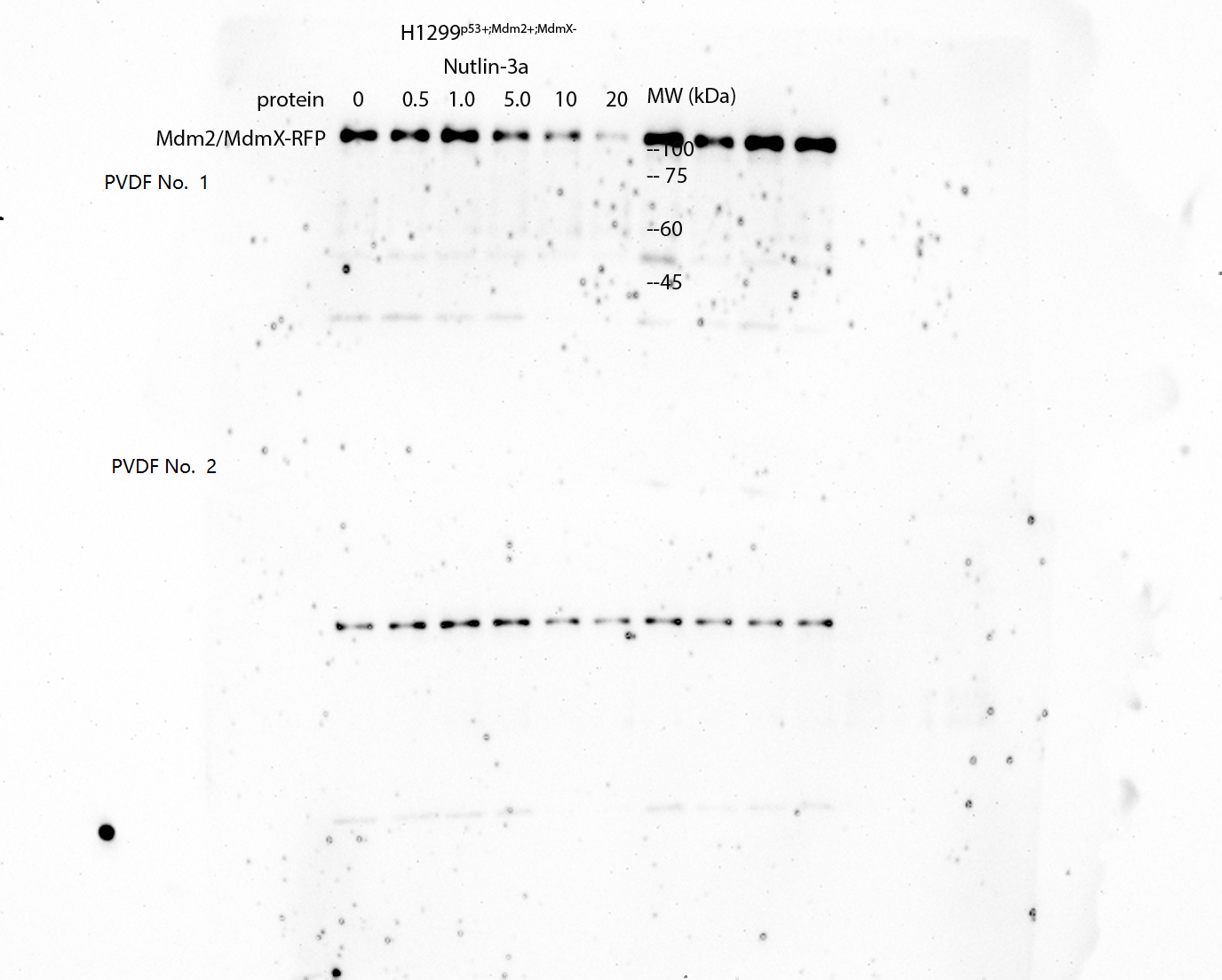

Supplement: Supplementary file 3 — Source Data [file 41467_2022_28721_MOESM3_ESM.zip › Source data/Source data for Supplementary Fig18/RFP/Supplementary Fig18c_Mdm2_MdmX_nutlin3a.Tif]

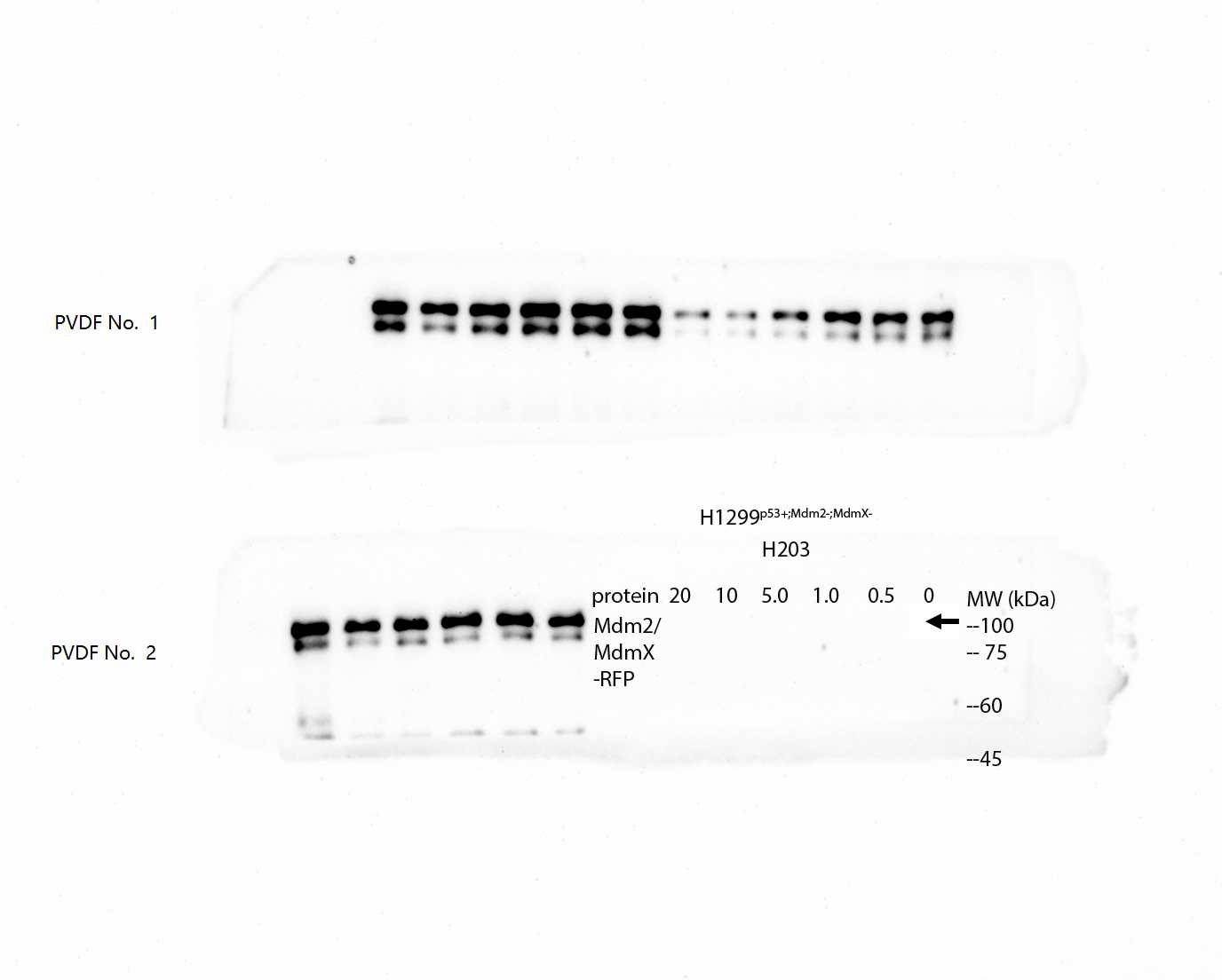

Supplement: Supplementary file 3 — Source Data [file 41467_2022_28721_MOESM3_ESM.zip › Source data/Source data for Supplementary Fig18/RFP/Supplementary Fig18d_Mdm2_MdmX_H203.Tif]

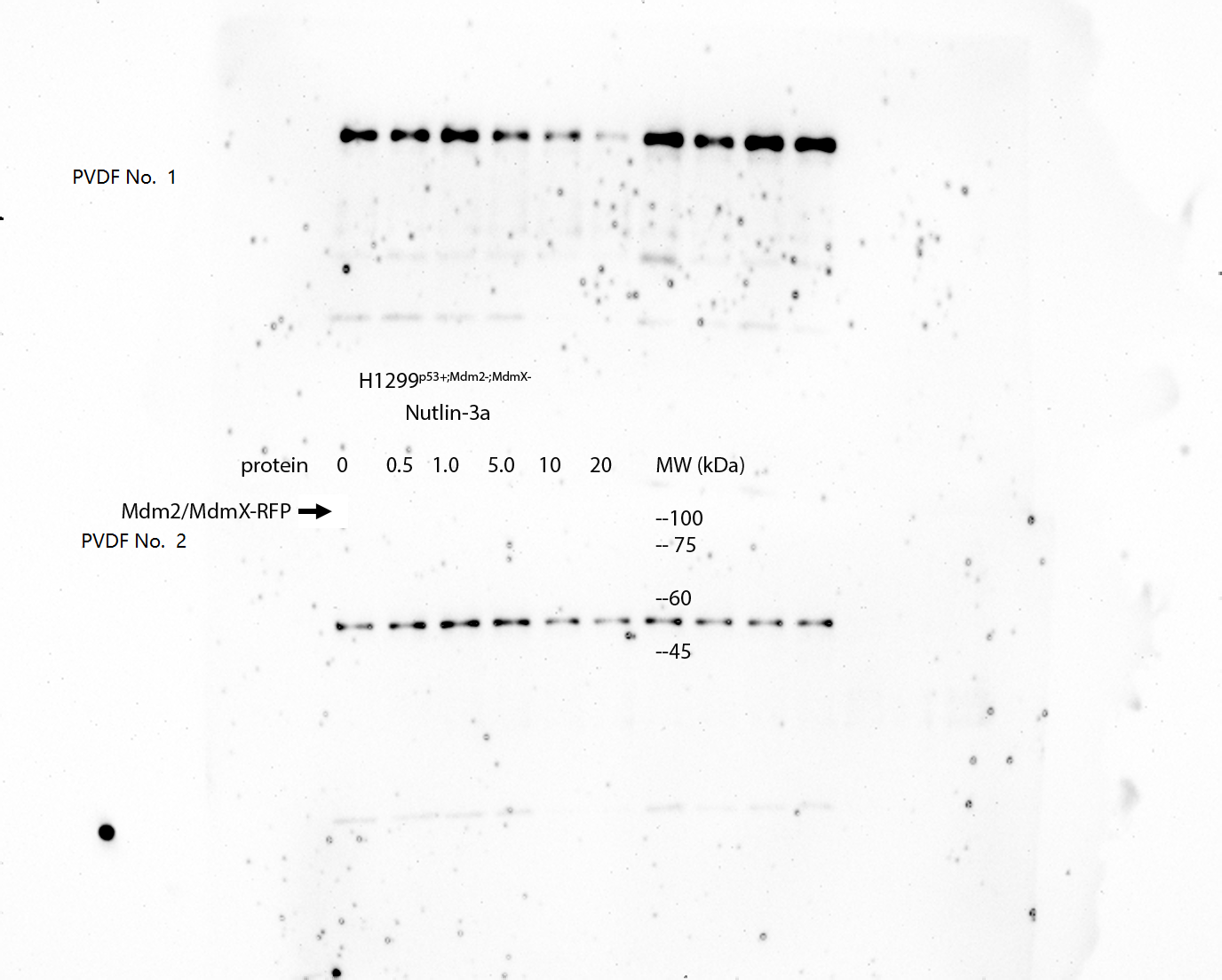

Supplement: Supplementary file 3 — Source Data [file 41467_2022_28721_MOESM3_ESM.zip › Source data/Source data for Supplementary Fig18/RFP/Supplementary Fig18d_Mdm2_MdmX_nutlin3a.Tif]
